# Human papillomavirus associated cervical lesion: pathogenesis and therapeutic interventions

**DOI:** 10.1002/mco2.368

**Published:** 2023-09-14

**Authors:** Jiatian Ye, Lan Zheng, Yuedong He, Xiaorong Qi

**Affiliations:** ^1^ Department of Gynecology and Obstetrics Key Laboratory of Birth Defects and Related Diseases of Women and Children (Sichuan University), Ministry of Education, West China Second Hospital, Sichuan University Chengdu China; ^2^ Department of Pathology and Lab Medicine University of Texas MD Anderson Cancer Center Houston Texas USA

**Keywords:** cervical cancer, cervical preneoplastic lesions, human papillomavirus, pathogenesis, therapy

## Abstract

Human papillomavirus (HPV) is the most prevalent sexually transmitted virus globally. Persistent high‐risk HPV infection can result in cervical precancerous lesions and cervical cancer, with 70% of cervical cancer cases associated with high‐risk types HPV16 and 18. HPV infection imposes a significant financial and psychological burden. Therefore, studying methods to eradicate HPV infection and halt the progression of precancerous lesions remains crucial. This review comprehensively explores the mechanisms underlying HPV‐related cervical lesions, including the viral life cycle, immune factors, epithelial cell malignant transformation, and host and environmental contributing factors. Additionally, we provide a comprehensive overview of treatment methods for HPV‐related cervical precancerous lesions and cervical cancer. Our focus is on immunotherapy, encompassing HPV therapeutic vaccines, immune checkpoint inhibitors, and advanced adoptive T cell therapy. Furthermore, we summarize the commonly employed drugs and other nonsurgical treatments currently utilized in clinical practice for managing HPV infection and associated cervical lesions. Gene editing technology is currently undergoing clinical research and, although not yet employed officially in clinical treatment of cervical lesions, numerous preclinical studies have substantiated its efficacy. Therefore, it holds promise as a precise treatment strategy for HPV‐related cervical lesions.

## Graphical abstract

This review comprehensively explores the mechanisms underlying HPV‐related cervical lesions, including the viral life cycle, immune factors, epithelial cell malignant transformation, and host and environmental contributing factors. Additionally, we provide a comprehensive overview of treatment methods for HPV‐related cervical precancerous lesions and cervical cancer currently and future.

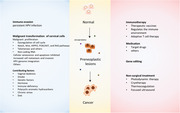

## 1 INTRODUCTION

Human papillomavirus (HPV) infection is highly prevalent among women of reproductive age.^1^ Based on its pathogenicity, HPV is classified into high‐risk and low‐risk types. High‐risk HPVs (HR‐HPVs) include types 16, 18, 31, 33, 35, 39, 45, 52, 56, 58, 59, 66, and 68.^2^ The majority of HPV infections are asymptomatic and self‐resolve within 12−24 months after infection. However, a small subset of infections persist or progress to preneoplastic lesions, ultimately resulting in cancer.^3^ The most prevalent types of carcinogenic HPV are HPV16 and 18, which are associated with approximately 70% of HPV‐related cervical cancers.^4^, ^5^

The HPV virion exhibits a spherical, unenveloped morphology characterized by a 20‐sided cubic symmetry. It comprises an approximately 8 kb circular double‐stranded DNA genome enclosed within a protein shell composed of L1 and L2. The virion's diameter ranges from 50 to 60 nm. The genome is organized into three functional sections: the early (E) region (E1–E8), the late (L) region (L1, L2), and the long control region (LCR).^6^ The E region encodes seven viral nonstructural proteins: E1, E2, E1ˆE4, E5, E6, E7, and E8ˆE2. These proteins are involved in HPV replication, transcription, translation, and transformation. The L region encodes two viral capsid proteins: L1 and L2. The LCR region, also referred to as the upstream regulatory region (URR), does not encode any proteins.^7^ Persistent HR‐HPV infection leads to cervical disease, ultimately progressing to cervical cancer.^8^ The transformation zone (TZ), located at the junction between the ectocervix and endocervix (squamocolumnar junction), serves as the origin of cervical lesions.^9^, ^10^ Cervical preneoplasia, also referred to as cervical intraepithelial neoplasia (CIN), is further categorized into CINI, CINII, and CINIII. CINI corresponding to low‐grade squamous intraepithelial lesions (LSIL) regresses spontaneously in approximately 80−90% of cases.^11^, ^12^ During this stage, the virus undergoes active replication,^13^ and mild changes occur in cervical epithelial cells. CINII‐III corresponds to high‐grade squamous intraepithelial lesions (HSIL).^14^ Despite the relatively low viral replication during this stage, the virus persists due to viral immune evasion, dysregulated vaginal microenvironment, and other cofactors.^15^, ^16^ Viral oncoproteins expression disrupts the cell cycle,^17^ and the viral genome can integrate into the host genome.^18^ Infected cells proliferate uncontrollably and undergo malignant transformation,^19^ ultimately resulting in cervical cancer.

The development of HPV preventive vaccines represents a significant milestone in the prevention and treatment of cervical cancer. These vaccines have demonstrated efficacy in reducing the incidence of cervical cancer and preneoplastic lesions.^20^, ^21^ However, due to factors such as cost, preventive vaccines have not gained widespread accessibility in economically underdeveloped areas. Furthermore, preventive vaccines have limited efficacy against existing HPV infections and cervical lesions.^22^ Currently, no specific treatment exists for HPV infection, emphasizing the clearance of infection and reversion of precancerous lesions as the main treatment focus for HPV‐related cervical lesions. The standard treatment for cervical cancer remains a combination of surgery and chemoradiotherapy.^23^ However, patients with advanced, metastatic cervical cancer still face a gloomy clinical prognosis despite undergoing standard therapy.^24^ In 2014, bevacizumab gained approval for treating metastatic cervical cancer.^25^ Later, in 2018, pembrolizumab was granted approval for recurrent and metastatic cervical cancer.^26^ The introduction of these targeted drugs has ignited extensive clinical research, with a particular focus on investigating combinations of targeted drugs, immunomodulatory drugs, and standard radiochemotherapy. The development of these clinical trials and the exploration of novel targeted drugs hold promise for future patients with advanced cervical cancer.

This review presents a comprehensive summary of the molecular and cellular pathogenesis of HPV‐related cervical lesions. It encompasses the viral life cycle, immune factors, epithelial cell malignant transformation, and contributions from the host and environment. The goal is to offer a detailed elucidation of the pathogenic mechanisms underlying HPV‐related cervical lesions. Additionally, we examine nonsurgical treatments for HPV‐associated cervical preneoplastic lesions and cervical cancer. Surgical interventions and radiation or chemotherapy fall outside the scope of this review and are not covered. Our focus lies on immunotherapy, encompassing HPV therapeutic vaccines, immune checkpoint inhibitors, and advanced adoptive T cell (ATC) therapy. These strategies hold potential for introducing novel treatment avenues when integrated with standard‐of‐care approaches. Furthermore, we outline frequently used drugs and other nonsurgical treatments currently employed in clinical practice for managing HPV infection and precancerous lesions. Moreover, emerging gene editing technology has exhibited effectiveness in numerous preclinical studies, showing promise as a precise treatment strategy for HPV‐related cervical lesions. This comprehensive review aspires to provide valuable references for clinical and preclinical research on HPV‐associated cervical lesions in the future.

## 2 HPV AND ITS PATHOGENESIS

### 2.1 Life cycle of HPV

**FIGURE 1:**
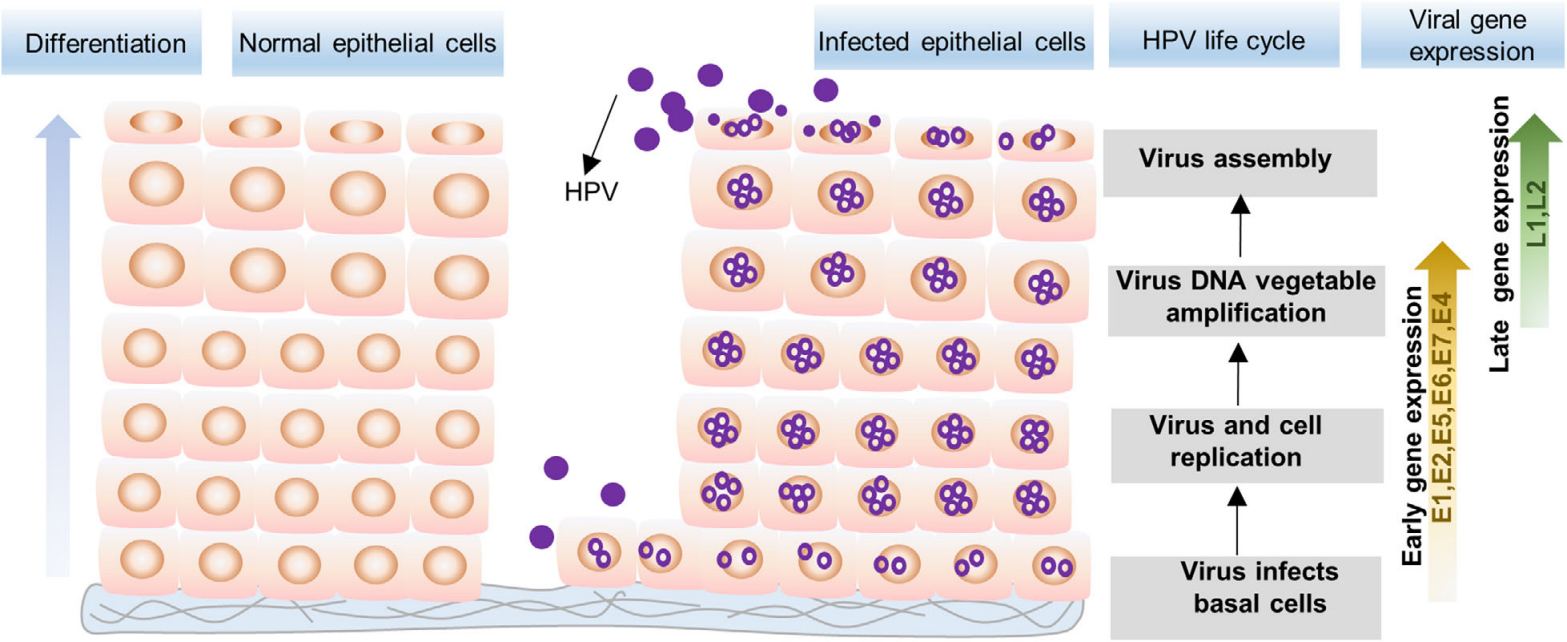
Life cycle of human papillomavirus. HPV entry to the basal cells, once it enters into the cells, HPV initiates its genome replication, which is mainly dependent on the E1 and E2 proteins; the expression of E6 and E7 contributes to promote host cell replication and prevent apoptosis. With the differentiation of epithelial cells, the HPV capsid proteins L1 and L2 express on the upper epithelial cells and complete the assembly and release of viral particles.

HPV targets the basal layer of the stratified squamous epithelium. After entering the host cell, it initiates initial replication and differentiation‐induced replication, culminating in the assembly and release of viral particles within the upper epithelial cells. Comprehending the virus's life cycle and the pertinent host factors is paramount for treating HPV infection (Figure 1).

The HPV life cycle commences with the infection of cells in the basal layer of the stratified squamous epithelium, which occurs due to disruptions in the epithelial barrier caused by minor injuries. In the normal epithelium, the basal cells represent the sole proliferating cells, in contrast to the differentiated cells in the upper layer have exited the cell cycle.^27^ In the initial infection stage, the virus traverses cellular barriers, including the plasma membrane and the nuclear envelope. This process is known as virus entry. For HPV16, the virus initiates its interaction by binding to heparan sulfate proteoglycans (HSPGs) on the epithelial cell surface and basement membrane (BM) via the L1 major capsid protein. Following HPV entry, a series of signaling processes ensues, along with interactions between the virus and secondary entry receptor molecules, including tetraspanin family members (CD63, CD9, and CD151), integrin complexes, growth factor receptors, and the phospholipid binding protein annexin.^28^, ^29^, ^30^ HPV enters the cytoplasm through independent endocytosis. Following cytoplasmic entry, the HPV capsid undergoes uncoating during acid endocytosis, a process that can be facilitated by host cell cyclophilin,^31^ heterotetrameric annexin A2/S100A10 (A2t),^32^ and other factors. HPV enters the endosome and subsequently traffics to the trans‐Golgi network and Golgi apparatus. The virus must traverse these compartments to reach the nucleus and initiate infection. Previous studies have indicated the crucial role of the minor L2 capsid in intracellular transport.^33^, ^34^, ^35^, ^36^ Cell‐penetrating peptides facilitate the insertion of L2 into the endosome membrane.^37^, ^38^ Subsequently, retromer recruitment ensures stable transient membrane insertion of the L2 capsid protein.^39^, ^40^ This step is pivotal for productive trafficking. γ‐Secretase can promote L2 membrane insertion.^39^ The GTPase‐activating protein folliculin safeguards incoming HPV virions from lysosomal degradation,^41^ similar to the role of coat protein complex I.^42^ The virus remains within in the Golgi until cell cycle progression into mitosis, when the breakdown of the nuclear membrane facilitates the accumulation of viral DNA at promyelocytic leukemia nuclear bodies, where is the site of viral transcription and replication.^31^, ^37^, ^43^ However, the process of intranuclear delivery and the cellular proteins involved in these steps remain uncertain. Several proteins, such as Ran‐binding protein 10, karyopherin alpha, and dynein light chain DYNLT3, can bind to L2 and assist in its transport toward mitotic chromatin.^36^ Rizzato's study demonstrated that the CDK1 and PLK1 sequentially phosphorylate the chromosome binding region of L2 to regulate the delivery of HPV viral DNA to mitotic chromatin during mitosis.^44^ Additionally, the autophagy adaptor p62 may also have involvement in the nuclear delivery process.^43^ Numerous studies have investigated the mechanism of HPV entry. However, most of these studies have concentrated on the function of individual or a limited number of proteins, and the precise process remains incompletely understood.

Following successful delivery, the viral genome undergoes amplification during the establishment phase. During the initial replication stage, the viral genome maintains at 20−50 copies per cell, and this count remains stable during cell proliferation in HPV‐infected cells. The viral oncoprotein E1 and E2 play a critical role in replication initiation.^45^, ^46^, ^47^ E2, functioning as a DNA binding factor, binds to the palindromic DNA sequences within the LCR that surrounds the viral origin of replication. This interaction facilitates the recruitment of the viral helicase E1, initiating replication of the viral genome.^48^, ^49^, ^50^ Nonetheless, E1 does not appear to be indispensable for the initial replication.^51^ E1 and E2 are the sole two virus‐encoded proteins required for DNA replication. The remaining replication proteins are derived from the host cellular DNA replication machinery. E1 and E2 are phosphorylated proteins, and their biological functions and replication of HPV genomes can be regulated by an array of cellular protein kinases, including CK2a, PYK2, MK2, and P38 mitogen‐activated protein kinase (MAPK).^52^, ^53^, ^54^, ^55^, ^56^ Phosphorylation of E2 by CK2 enhances its interaction with TOBP1, enabling the localization of the viral genome to chromatin sites that support viral replication during mitosis. Additionally, CK2 phosphorylation helps maintain the stabilization of E2 during cell differentiation.^57^, ^58^, ^59^ The E2 protein binds to both the viral genome and host chromatin simultaneously, ensuring the retention of viral genomes in daughter nuclei upon the completion of mitosis.^58^ This function is termed the plasmid segregation function of the E2 protein, which is pivotal for sustaining the episomal genome.^59^ In the initial stage, virus protein E6/E7 expression is restricted,^27^ a modest level of E6/E7 is enough to target P53 and PRb, fostering cell proliferation to support virus replication.

The viral life cycle correlates with the differentiation of infected cells, during which the virus migrates from the basal layer to the spinous layer, triggering the late stage of the viral life cycle. This stage involves vegetative genome replication and the expression of the capsid proteins L1 and L2. Though the mechanism underlying the elevation in HPV DNA synthesis remains unclear, it appears to be linked to the downregulation of the CCCTC‐binding factor‐associated Yin Yang1 transcription factor.^60^ Additionally, disruptions in viral genome looping and loss of epigenetic repression of viral enhancer activity^27^ may be contributing factors. The upregulation of E6/E7 expression and downregulation of E2^^^E8 expression also promote genome replication.^61^ Murakami et al.’s^62^ research demonstrated that nucleosome positioning and its chemical and compositional modifications may have essential roles in genome regulation.

Regarding the mechanism of virus genome replication, in the initial infection stage, the HPV genome undergoes E1‐ and E2‐dependent bidirectional replication, which is essential for maintaining genome integrity. This replication process can be regulated by the Werner helicase.^63^ As the viral genomes oligomerize, the virus genome starts replicating through break‐induced replication (BIR). BIR becomes activated under stress conditions to repair double‐strand breaks and collapsed classical replication forks.^64^ HPV oncoproteins trigger the activation ATR and ATM pathways by inducing a substantial count of DNA breaks.^65^ Furthermore, they recruit DNA damage factors, such as pATM and pCh2,^66^ to promote the viral genome replication. Additionally, topoisomerase 2β contributes to this critical process by inducing double‐strand DNA breaks.^67^, ^68^, ^69^

Enhanced read‐through at the early HPV16 polyadenylation signal into the late region of the HPV16 genome results in HPV late gene expression, leading to the production of HPV16 late L2 mRNAs.^70^ Following the activation of late promoters, the expression of L1 and L2 ensues, ultimately leading to the complete formation of virions. This process occurs in the upper epithelial layers, allowing the virus to complete its life cycle and consequently leading to the development of benign or malignant proliferative lesions of the cervix.

### 2.2 Immune evasion and persistent HPV infection

The majority of HPV infections are resolved within 1−2 years. However, persistent HPV infection can lead to cervical lesions and ultimately cervical cancer. Various mechanisms of immune evasion induced by HPV can lead to immune tolerance, enabling persistent HPV infection. Patients with HPV‐related cervical lesions exhibit an immunosuppressed microenvironment. Additionally, individuals with HIV are more prone to developing HPV infection and HPV‐related cervical lesions, providing further evidence of the immune factors in the development of HPV‐related cervical lesions.

In normal women, HPV infection can be eliminated through innate and adaptive immune responses, with only a small portion of HPV infections becoming persistent. Innate immune response, mounted by dendritic cells (DCs), macrophages, natural killer cells, and natural killer T cells, which serve as the first line of defense against HPV infection.^71^ The subsequent defense entails cytotoxic T lymphocytes (CTLs) targeting the HPV oncoprotein appears to eliminate HPV‐infected cells. However, HR‐HPV have developed various mechanisms to evade the host's immune response.

#### 2.2.1 Innate immune response

HPV can influence the pattern recognition receptors (PRRs) and downstream pathways, thus disrupting virus recognition and inhibiting interferon (IFN) responses. It can also impact the functionality of innate immune cells and modulate cytokine expression, which is not beneficial for virus clearance.

Both innate immune cells and keratinocytes possess the ability to express PRRs capable of identifying microbial pathogens and damage‐associated signals, including molecular patterns and damage‐associated molecular patterns. These PRRs encompass Toll‐like receptors (TLRs), nucleotide binding oligomerization domain‐like receptors (NLRs), retinoic acid‐inducible gene‐I‐like receptors (RLRs), and cytosolic DNA sensors.^15^ They constitute the first line of defense against foreign invaders. Suppression of the expression of PRRs and alteration of downstream cascades can both contribute to immune evasion. In the context of HPV16 infection, there is an alteration in the expression of TLRs. After infection, TLR2 and TLR7 are significantly downregulated, while the TLR4 is conversed regulated.^72^ Cytosolic DNA sensors IFI16 and STING are found to be upregulated after HPV infection. During the early stage of HPV life cycle, the virus can evade the cGAS/STING surveillance through vesicular trafficking.^73^ Additionally, HPV16 E6 can inhibit the dsRNA sensor RIG‐I by promoting the degradation of TRIM25.^74^ HPVE5 can inhibit both the cGAS–STING and the RIG‐I/MDA5 axis, effectively suppressing the virus recognition machinery.^75^

The IFN response constitutes a pivotal facet of host's innate immune. Most type I IFNs are induced through the binding of viral products to PRRs, activating IFN factors (IRFs) and NF‐κB, which subsequently stimulate the synthesis of IFN molecules.^76^, ^77^ Type I IFNs facilitate the phosphorylation and dimerization of signal transducer and activator of transcription1 (STAT1) and STAT2 upon binding to the type I receptor. This process induces the transcription of IFN‐stimulated genes (ISGs), thereby impeding viral replication and spread. HPV can hinder the IFN response by affecting PRRs. cGAS/STING can induce IRF3‐depedent antiviral IFN production upon sensing cytosolic DNA,^78^ while HPV16 can degrade STING through NLRX1 mediation, thereby impacting the IFN response.^79^ Virus oncoproteins can directly disrupt key modules and genes to evade the IFN response. The E1 protein can downregulate immune response genes, including IFNβ1 and IFNλ1 and ISG.^80^ E6 and E7 also appear to collaboratively repress the transcription of ISG.^81^, ^82^ Furthermore, both E6 and E7 employ distinct mechanisms to target the IFN signaling pathway. E7 can bind to and inhibit the transactivating function of IRF1 and can mediate transcription activation of chromatin repressor SUV39H1. This action promotes the epigenetic silencing of RIG‐1, cGAS, and STING, effectively halting IFN secretion.^83^ Meanwhile, E6 binds to IRF3, suppressing its transcriptional activity, and it can also disrupt JAK–STAT activation by binding to Tyk2.^81^, ^84^ HPV16 E5 has been observed to suppress the IFN signal in both infected cells and stromal microenvironment.^85^ This suppression potentially involves EGFR and TGFBR2 signaling pathways.^86^

HPV impacts the migration, maturation, and differentiation of innate immunes cells. DCs represent the pivotal mediators of innate immunity. HPV16 E2 can hinder the migrations of DCs by inducing overproduction of prostaglandin E2.^87^ HPV E6 can downregulate CD40 through E6/p53/AKNA (AT‐Hook Transcription Factor) axis, thereby influencing the function of DCs.^88^ In early cervical lesions, HPV16 and 18 can impede the increased natural killer cells, with the effect of HPV16 proving more pronounced.^89^ Researchers found that the inhibitory molecules TIGIT and KLRG1 on NK cells increased in HPV16‐associated cervical lesions, thereby impacting NK cell‐mediated immune responses.^90^

HPV viral oncoproteins can modulate the production of several cytokines, thus influencing both innate and adaptive immunity.^91^ HPV16 E6/E7 can obstruct the production of IL‐1β by inhibiting the binding of IRF6 on the IL‐1β promoter, which is critical in host defenses against injury and infection.^92^ The IL‐17 family cytokines play an important protective role in immunes response, while the level of IL‐17 was found to be low in HPV‐infected patients.^93^ HPVE6/E7 also downregulate the IL‐2 and impact HPV clearance.^94^ Additionally, both E6/E7 and E6* from HR‐HPV can elevate the expression of IL‐6 in keratinocytes, fostering a proinflammatory microenvironment that facilitates the progression of cervical lesions.^95^ Furthermore, it was found that E7 can interact with several core members (NEMO, CK1, and β‐TrCP) of both the NF‐κB and Wnt/β‐catenin signaling pathways, effectively inhibiting host defense peptide expression.^96^ Moreover, HPV can elevate the expression of human leukocyte antigen (HLA)‐G, favoring infected cells to escape detection by immune surveillance cells and promoting the persistence of HPV.^97^

#### 2.2.2 Adaptive immune response

Antigen‐presenting cells (APCs) play a crucial role in initiating adaptive immunity by presenting the antigen to CD4+ T help and CD8+ CTLs through major histocompatibility complex (MHC) II and MHC I molecules, respectively. In addition to their effects on DC differentiation and proliferation within the context of innate immunity, HPV oncoproteins can also impact adaptive immunity by influencing antigen presentation. Specifically, HPV16 E6 can impair the function of the transporter associated with antigen‐processing complex.^98^

The persistence of HPV infection largely depends on the composition of T cell subsets within the tumor microenvironment. Infiltration of T cells that suppress tumor growth can impede cancer progression, while the opposite outcome is also plausible. Effective CTL responses frequently rely on support from T helper cell. Impairment of the T helper cell response can lead to CD8+ T‐cell exhaustion and failure, impeding the resolution of persistent infection. Bashaw's study demonstrated that HPV16 E7‐driven epithelial hyperplasia can attenuate Th1 immunity and steer T‐cell differentiation toward a regulatory or anergic phenotype.^99^ Furthermore, E7‐mediated epithelial hyperplasia can augment the population of peripheral regulatory T cells and suppress antigen‐specific CD8+T‐cells.^100^, ^101^ Reports indicate that IFI16 can elevate PD‐L1 through STING–TBK1–NF–κB pathway, thus aiding cells in evading immune attack.^102^ HPV E6 and E7 can also increase PD‐L1 expression through miR‐142‐5p/PD‐L1 axis.^103^ Additionally, in HPV‐associated cervical cancer, the m6A regulator METTL3 was found to be upregulated and promote the expression of PD‐1,^104^ which can hinder the cytotoxic of CD8+T cells. The role of humoral immunity in viral clearance primarily depends on antibody production. In the life cycle of HPV, the expression of L1 and L2 is restricted to the upper epithelial layers, leading to delayed immune recognition.^105^ In the cases of mastomys natalensis papillomavirus, researchers found that virus express different L1 isoforms to escape adaptive immune responses. During the early stages of HPV infection, the virus employs an extended version of the L1 protein to elude immune system detection. This strategy capitalizes on the fact that antibodies recognizing the lengthier L1 protein lack neutralizing capacity and are incapable of hindering virus dissemination. Consequently, the virus is afforded ample time to establish infection.^106^

#### 2.2.3 Pyroptosis

Pyroptosis, a profoundly inflammatory mode of programmed cell death, can be incited by inflammasomes and serves as a defense mechanism against pathogenic infection. Researchers found that HPV E7 can inhibit cell pyroptosis by facilitating TRIM21‐mediated degradation and ubiquitination of the IFI16 inflammasome, which also contributes to an essential immune evasion strategy.^107^

### 2.3 Malignant transformation of HPV‐infected cells

**FIGURE 2:**
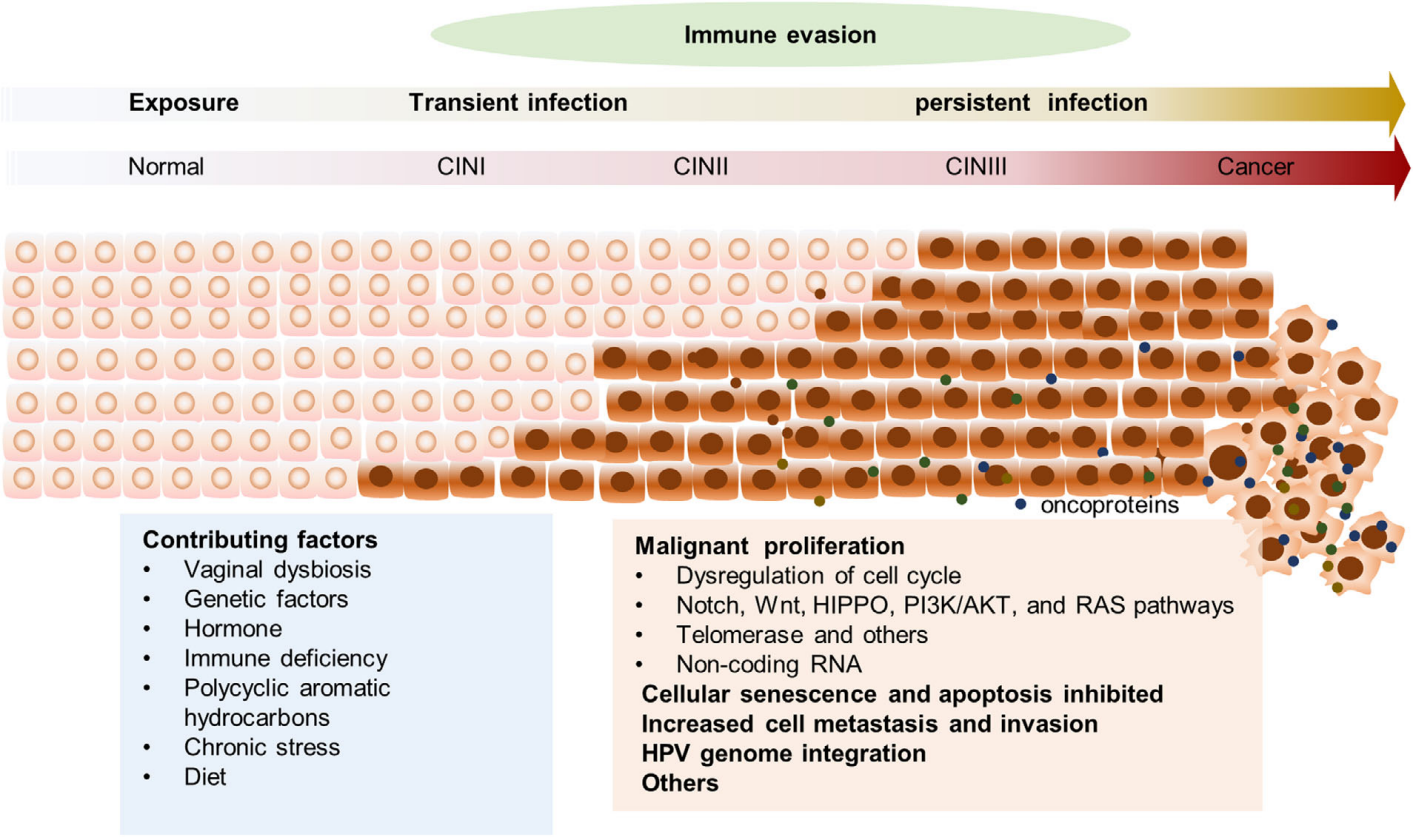
Pathogenesis of HPV infection‐related cervical lesion. Under persistent HPV infection, cervical lesion progress from preneoplastic lesions (CINII‐III) to cervical cancer. During this progress, besides immune factors, the cervical cells undergo a series of malignant transformation because of the virus oncoproteins and gene, including malignant proliferation, cellular senescence, apoptosis inhibited, increased cell metastasis and invasion, and HPV genome integration. The contributing factors, such as vaginal dysbiosis and others, work together, leading to the progression of cervical lesion and cervical cancer finally.

The development of cervical lesions entails molecular modifications beyond persistent HR‐HPVs infection. Changes in these molecules result in alterations in cell phenotypes, encompassing cell cycle regulation, proliferation, death, adhesion, and aggressiveness. Moreover, modifications in the host genome both form the basis of molecular changes and engage with them. Over time, these aberrant cell phenotypes gradually accumulate, propelled by abnormal cell metabolism and oxidative stress. Subsequently, cells undergo a progressive malignant transformation, eventually leading to the onset of cancer (Figure 2).

#### 2.3.1 Abnormal regulation of the cell cycle and malignant cell proliferation

Human cells undergo mitosis to ensure the preservation of chromosomal stability in progeny cells. The cell cycle encompasses the complete process from the completion of one division to the conclusion of the subsequent division. It is divided into interphase and division phase. Interphase is subdivided into three stages: G1 phase, S phase, and G2 phase. The M phase or mitosis, during which DNA replication occurs in the S phase. The cell cycle is governed by five checkpoints: G1/S phase checkpoint, S phase checkpoint, G2/M phase checkpoint, DNA replication checkpoints, and spindle assembly checkpoints. These checkpoints can temporarily arrest the cell cycle, enabling genetic information editing, repair, or induce apoptosis. HPV disrupts checkpoint integrity, granting cells multiple re‐entries into the cycle, fostering malignant cell proliferation. Cell cycle regulation occurs at the downstream level, while abnormal alterations in upstream signaling pathways and host proteins involved in cell proliferation can also contribute to abnormal cell proliferation and malignant transformation. Additionally, HPV can modulate cell proliferation by upregulating or downregulating specific noncoding RNAs. Malignant cell proliferation is a crucial characteristic in the malignant transformation of normal cells, which significantly enhances comprehension of HPV‐triggered cervical lesions and cervical cancer pathogenesis.

##### Dysregulation of cell cycle‐related proteins

Proper cell cycle regulation relies on diverse proteins, including cyclins and cyclin‐dependent kinases (CDKs), cyclin‐dependent kinase inhibitors (CKIs), the retinoblastoma protein (RB) family, the E2F transcription factor family, and the p53 family. Dysregulation of these proteins has been noted in HPV‐infected cells, leading to aberrant cell cycle entry and unrestrained proliferation.

*Cyclins and CDKs*: The proper progression of the cell cycle relies on the interplay between cyclins and CDKs, cyclins coordinate the progress of the cell cycle by binding to CDKs. Elevated Cyclin D1 expression has been observed in specific SILs arising from persistent HR‐HPV infection in cervical epithelial cells.^108^ However, the precise mechanism underlying this dysregulation remains unclear. Researchers have discovered that E6 can enhance AurA expression, and their interaction can influence the expression of cyclin E and phosphorylated Histone H3. These proteins play vital roles in the G1/S and mitotic phases, disrupting cell cycle regulation in HPV‐positive cells.^109^

*CKI family*: CKIs hold pivotal roles in regulating cell cycle progression. The Kip/Cip family, which includes three structurally related proteins (p21, p27, and p57), and the INK4 protein family, consisting of four related proteins (p16, p15, p18, and p19), constitute critical CKI families. HPV oncoproteins can directly or indirectly influence CKIs. HR‐HPV has the capacity to selectively recruit USP46, a cellular deubiquitinase that stabilizes Cdt2 that can regulate the S phase of the cell cycle by degrading p21, Set8, and Cdt.^110^ Although p16 generally functions as a tumor suppressor, in HPV‐transformed cervical cancer, E7 facilitates the degradation of Rb. As a result, p16 exhibits oncogenic activity, fostering cervical carcinoma proliferation through the CDK–HuR–IL1A axis.^111^

*Rb family and E2F transcription factors family*: The human family of E2F transcription factors primarily regulated by the RB protein family, comprising RB1 (pRb), RBL1 (p107), and RBL2 (p130). Phosphorylated retinoblastoma protein (pRb) functions as a pivotal rate‐limiting factor for cyclin–CDK complex‐mediated phosphorylation during the G1 phase, constituting a central element of the G1/S regulatory checkpoint. pRb liberate bound E2Fs, allowing unbound E2F to translocate to the nucleus, where it engages with gene promoter regions possessing specific sequences, such as c‐myc, the promoter region of the E2F‐1 gene. This engagement facilitates genes expression. HPV16/18 can disrupt nuclear localization of p130, contributing to cell cycle deregulation.^112^ RB‐like proteins p107 and p130 facilitate the formation of DREAMC, a multisubunit DNA binding complex that acts as an evolutionarily conserved transcriptional repressor, impeding cell‐cycle‐regulated genes transcription.^113^ E2F1 is pivotal in G1/S checkpoint. E7 can enhance the expression of general control nondepressible 5, which regulates the expression of E2F1 by acetylating H3K9 in the E2F1 promoter.^114^, ^115^ In cervical cancer cells, the E7 oncoprotein propels proliferation through E2F.^116^

*P53 family*: The tumor suppressor p53 stimulates the expression of p21, culminating in G1 phase cell cycle arrest. The degradation of p53 is a hallmark of α‐HPVs and HPV‐associated carcinogenesis. The E6 oncoprotein forms a ternary complex with the E3 ubiquitin ligase E6‐associated protein (E6AP) and tumor suppressor protein p53, leading to the ubiquitination of p53.^117^, ^118^ Abnormal nuclear export of HP1γ, mediated by exportin‐1, is responsible for E6‐mediated degradation of p53.^119^ In specific contexts, tumor suppressors can exhibit proproliferation functions. Researchers discovered that HPV38 E6 and E7 induce the accumulation of a wild‐type p53 form in human keratinocytes (HKs), promoting cellular proliferation.^120^

*Others*: Recent studies have unveiled the anaphase‐promoting complex/cyclosome (APC/C) coactivator, Cdh1, as a new target of E7. APC/C is responsible for targeting various cell cycle‐related proteins. The activity of APC/C‐Cdh1 spans from anaphase to cytokinesis and extends until the G1 phase. Inactivation of Cdh1 induced by HPV16 E7 disrupts the function of APC/C, leading to the abnormal accumulation of Cdh1 substrates, such as FoxM1.^121^ This accumulation can impede CKIs, which are frequently overexpressed in diverse malignancies and further augmenting cell proliferation.

##### Abnormal transduction of cell signaling and cell proliferation

The regulation of the cell occurs at the downstream level, and alterations in the upstream pathways can impact cell cycle regulation and cell proliferation, either directly or indirectly.

*Notch signaling pathway*: The Notch signaling pathway, critical for the growth of mammalian epithelial cells, constitutes a conserved cascade consisting of Notch receptors, Notch ligands, DSL proteins (Delta, Serrate, LAG), CSL (CBF‐1, suppressor of hairless, lag), DNA‐binding proteins, and other regulatory molecules.^122^ This pathway targets genes such as Cyclin D and CDK2. HPV16 selectively modulates delta‐like ligand (DLL)‐Notch1 signaling, propelling squamous cell proliferation. Additionally, HPV E6 induces sustained DLL4 expression in keratinocytes, promoting proliferation while limiting keratinocyte differentiation through downregulation of DLL1.^123^ Different HPV‐E6 genera appear to exhibit varying Notch signaling pathway activation. β HPV E6 proteins repress Notch reporter expression, with HPV38 E6 displaying notable repression potential. In contrast, α‐HPV E6, particularly HPV16, prominently activates reporter expression, while HPV18 E6 yields no discernible effect. This diversity in Notch pathway targeting might bear implications for potential HPV therapies.^124^

*Wnt signaling pathway*: The Wnt signaling encompasses three routes: Wnt /β‐catenin, Wnt /Ca^2+^, and Wnt /pcp pathways. The Wnt /β‐catenin pathway represents the classical pathway of Wnt signaling. Cyclin D and cyclin E are the downstream target genes of Wnt signaling. Activation of the Wnt pathway increases cytoplasmic β‐catenin, subsequently fueling the activation of cyclin genes, culminating in cellular division, growth, and uncontrolled proliferation. HPV18 E6 diminishes MAGI3 levels, contributing to Wnt /β‐catenin signaling activation.^125^ E6 proteins from HR‐HPV and LR‐HPV activate the Wnt/ β‐catenin pathway by degrading NHERF1 through E6AP.^126^ Besides, HPV‐18 E6 oncoprotein and its spliced isoform E6*I regulate the Wnt/β‐catenin cell signaling pathway through upregulating TCF‐4 transcriptional factors.^127^ In addition to activating the classical Wnt pathways, HPV oncoproteins also activate nonclassical pathways. Research demonstrates that E6 preferentially augments the translation of WNT4, JIP1, and JIP2, thereby engendering noncanonical WNT/PCP/JNK pathway activation, fostering in vitro cell proliferation.^128^ Wnt signaling interfaces with diverse signaling pathways, yet several facets of these interactions remain enigmatic and warrant further investigation. The formulation of efficacious anticancer agents grounded in pathway mechanisms stands as a promising avenue for future research.

*HIPPO signaling pathway*: Extensive investigations have demonstrated that the HIPPO signaling pathway is a tumor‐suppressive pathway that meticulously governs cell proliferation and plays a significant role across distinct cancer development stages. Yes‐associated protein (YAP), a pivotal effector molecule of HIPPO, phosphorylates the transcriptional coactivator YAP and its homologous protein TAZ, ushering their cytosolic sequestration and degradation. Conversely, nonphosphorylated YAP/TAZ enters the nucleus and binds to the TEAD family and other transcriptional activators, activating the expression of target genes and promoting cell proliferation while suppressing apoptosis. The roster of YAP target genes includes cyclins and E2Fs, governing the cell cycle. YAP and TAZ concurrently serve as coactivators of cell proliferation‐associated genes, including CCN1 and MYC. The upregulation of E6/E7 in human cells downregulates PTPN14,^129^ thereby catalyzing YAP1 activation through LATS2 upregulation.^130^, ^131^ This configuration establishes a negative feedback loop pivotal for sustaining cervical epithelial cell homeostasis. However, HR‐HPV targets LATS2, perturbing the feedback loop and fostering the malignant transformation of cervical epithelial cells1.^132^ The dimeric conformation of HPV E6 assumes a critical function in the elevation of YAP/TAZ.^133^

*PI3K/AKT signaling pathway*: The PI3K/AKT signaling pathway plays a crucial role in regulating cell proliferation, differentiation, and migration, substantiating its involvement across numerous HPV‐associated cancers.^134^ AKT regulates the cell cycle by acting on downstream substrates, including CDKs, cyclins, and CKIs, thereby collectively orchestrating the regulation of cell proliferation. Notably, HPV triggers the upregulation of nuclear receptor‐related‐1 protein, which autonomously triggers the PI3K/Akt/mTOR signaling cascade, precipitating cell proliferation.^135^ HPVE7 orchestrates the aberrant expression of Non‐SMC (Structural Maintenance of Chromosomes) condensin I complex subunit H, consequently activating the PI3K/Akt/SGK pathway. This activation culminates in the proliferation, EMT, and invasion of HPV‐infected cells, further promoting the emergence of cervical lesions.^136^ In HPVE7‐expressing cells, the activation of PI3K/AKT/mTOR promotes the serine 11 (S11) phosphorylation of chromosome condensation1, facilitating G1/S transition of HPV‐infected cells.^137^ Sustained expression of HPV16 E7 augments the expression of p‐AKT/P‐Src, propelling the progression of cervical precancerous lesions.^138^

*RAS signaling pathway*: The RAS pathway includes three classical conduits: RAF–MEK–ERK (MAPK), PI3K–Akt–mTOR, and RAL–GEF pathways, which play crucial roles in cell proliferation, survival, and differentiation. Abnormal regulation of the RAS pathway is intrinsically tied to the emergence of various tumors. Studies have shown that HPV oncoproteins can promote the malignant proliferation of HPV‐infected cells by modulating signaling pathways and the expression of upstream and downstream molecules. For instance, HPV8 E6 selectively targets protein–tyrosine–phosphatase H1, thereby instigating the sustained proliferation of keratinocytes by activating Ras.^139^

##### Host target proteins involved in cell proliferation

Telomerase, which is activated in all HPV‐associated cancers, can elongate telomeric DNA, hinder senscence and apoptosis, and bestow cell immortality. The rate‐determining factor for telomerase is the expression of its catalytic subunit, hTERT.^140^ HR‐HPVE6 relies on multiple cellular proteins, including NFX1‐123, which is upregulated in HPV16‐positive cervical cancer to regulate the activity of hTERT and telomerase.^141^, ^142^ HPV16 E6 binds to the promoter region of hTERT, inducing its expression and ultimatly activating the function of telomerase.^143^ Recently, researchers have found that HPVE6 interaction with AuyB, enhances hTERT expression and augments telomerase activity.^144^

In addition to telomerase, HPV oncoproteins engaget with other small molecule proteins to govern cell proliferation. Eukaryotic translation initiation factors facilitate protein synthesis, promoting cell proliferation by aiding the transfer of specific mRNAs and the expression of genes related to cell cycle regulation. Some of these genes are related to cell cycle‐related proteins. Studies have demonstrated that HPVE6 upregulates the expression of eIF5A‐1^145^ and modulates the activity of eIF4E protein through the MEK/ERK and AKT/PKB pathways.^146^ HPV16 E7 increases the expression of pyruvate kinaseM2 (PKM2) and trriggers its nonglycolytic function, fostering cervical cancer cell proliferation.^147^ HPV18 E7 enhances the transcription of ELK‐1, an activator that stimulates cell proliferation.^148^ HPV16 E6 upregulates silent information regulator1, promoting cervical cell proliferation.^149^ Knockdown of AIB7 expression in E1E6 immortalized human cervical cells significantly inhibits cell proliferation, underscoring AIB1 as a promising target for HPV E6 and a biomarker for cervical cancer progression.^150^

##### Noncoding RNA and cell proliferation

Noncoding RNAs such as microRNA (miRNA) and long noncoding RNA (lncRNA) also participate the regulation of cell proliferation. miRNA are a class of short regulatory noncoding RNAs, approximately 22 nucleotides long. They exert negative control over target gene expression and cellular processes, including differentiation, proliferation, and apoptosis, chiefly through the promotion of mRNA degradation or translational repression. lncRNAs are noncoding RNAs exceeding 200 nucleotides in length, undertaking a regulatory role in gene expression. Recent studies have revealed that lncRNAs assume either cancer‐promoting or cancer‐suppressing roles in tumor initiation and progression. They actively participate in the regulation of apoptosis, tumor invasion, and metastasis. In HPV‐infected cells, the HPV oncoproteins, especially E6 and E7, possess the capability to influence numerous noncoding RNAs, thus fostering processes such as promoting cell proliferation, migration, and invasion (Table 1).

**TABLE 1:** Noncoding RNA modulated by HPV oncoproteins related to cell proliferation.

Regulation by HPV oncoproteins	Types of noncoding RNA	Effects	Related signaling pathways	References
HPV16 E6	↓miR‐22	Promote cell proliferation and migration	ap53/miR‐22/HDAC6 pathway	^151^
HPV16/18 E6/E7	↓miR‐375	Promote cell proliferation, migration, and invasion	Not study	^152^
HPV16 E7	↓miR‐106a	Promote cell proliferation and autophagy	Not study	^153^
HPV16 E6/E7	↓miR‐34a	Promote cell proliferation and invasion	WNT/β‐catenin pathway	^154^
HPV E6/E7	↑miR‐18a	Promote cell proliferation	Hippo/YAP	^155^
HPVE6	↓miR‐148a‐3p	Promote cell proliferation, migration, and invasion	↑LIPG, activate PI3K/AKT/mTOR	^156^
HPV16 E6/E7	↑miR‐4454	Promote cell proliferation, invasion, and migration	Not study	^157^
HPV	↓miR‐218	Promote cell proliferation, invasion, and migration	Not study	^158^
HPV	↑LncRNA SNHG8	Promote cell proliferation and inhibit apoptosis	Not study	^159^
HPV16/18 E7	↑Lnc‐EBIC	Promote cell proliferation, migration, and inhibit apoptosis	Not study	^160^
Not study	↓lncRNA MCM3AP‐AS1	Promote cell proliferation and inhibit apoptosis	miR21/PTEN	^161^
HPV16 E6/E7	↑lncRNA SNHG12	Promote cell proliferation, migration, and invasion	ERK/Slug/E‐cadherin	^162^
HPV16 E7	↑lncRNA MALAT1	Promote cell proliferation, invasion, and migration	Not study	^163^, ^164^
HPV E6	↑lncRNA GABPB1‐AS1	Promote cell proliferation, migration	Not study	^165^
HPV16 E6	↑lncRNA FAM83H‐AS1	Promote cell proliferation, migration, and inhibit apoptosis	Not study	^166^

#### 2.3.2 Cellular senescence, apoptosis, and autophagy

Cellular senescence, apoptosis, and autophagy play a significant role in HPV‐related cervical lesions. HPV oncoproteins can disrupt the equilibrium of cell numbers by modulating molecules and signaling pathways associated with these processes. This leads to the buildup of aberrant cells and the facilitation of malignant transformation.

Cellular senescence is a common phenomenon in the biological realm. Aging can induce irreversible cell cycle arrest, acknowledged as an autonomous tumor suppression mechanism. Suppression of cell senescence can promote uncontrolled cell proliferation, ultimately contribute to malignant alterations. Activation of the DNA damage response triggers P53‐mediated cellular senescence. However, the E2 protein can bind to P53, attenuating its ability to induce cellular senescence in response to DNA damage.^167^ Notably, the HPV‐18 E2 protein can downregulate antisense noncoding mitochondrial RNA‐2, consequently delaying replicative senescence in HKs.^168^

Tissue cells maintain a quantitative balance through the interplay of proliferation and apoptosis. Disruption of this balance can contribute to the development of diseases, including cancer. Currently, apoptosis in eukaryotic cells is predominantly mediated by the death receptor‐mediated extrinsic pathway, the mitochondrial intrinsic pathway, the B‐granidase‐mediated pathway, and the near‐endoplasmic reticulum stress pathway. The composition of the apoptosis mechanism mainly involves four protein families: apoptosis proteases (caspases), adapter proteins, Bcl‐2 family proteins, and apoptosis inhibitory proteins. HPV E6 targets apoptosis‐inducing factor (AIF) for degradation, thereby obstructing AIF‐mediated apoptosis.^169^ Additionally, HPV16 E6 disrupts apoptosis by deregulating death domain‐associated protein and suppressing caspase‐8 activities.^170^ Furthermore, HPVE6 downregulates nuclear transport, impairing IFN‐γ‐dependent apoptosis.^171^ Moreover, HPVE1/E6 upregulates UHRF7, which suppresses the expression of tumor‐suppressor genes (TSGs), allowing cells to evade apoptosis and promoting cancer progression.^172^ The innate immune receptor NOD1 also participates in the apoptotic signaling pathway, but it can be downregulated by HPV E6/E7, inhibiting apoptosis of cancer cells.^173^ Recently, researchers have reported that lncRNA HIF1A‐AS2, regulated by HPV16 E6, restrains apoptosis in cervical cancer cells through the P53/Caspase 9/Caspase 3 axis.^174^

Autophagy involves the phagocytosis and degradation of damaged or redundant cell components, such as aging proteins and organelles, ultimately promoting cell survival. However, excessive autophagy can exhaust intracellular proteins and energy to an extent where cell viability is compromised, leading to apoptosis. Autophagy exhibits a dual role in cancer development. During the early stages of cell transformation, autophagy inhibits the generation of cancer cells. However, once cancer cells are established, autophagy can bolster cancer cell survival and suppress apoptosis. HPV E6/E7‐positive keratinocytes encounter pronounced replicative and oxidative stresses, which are counteracted by autophagy activity.^175^ Moreover, HPV16 E6/E7 activates autophagy through Atg9B and LAMP1.^176^ Additionally, HPV11 E6 was found to repress AKT/mTOR and Erk/mTOR, culminating in autophagy activation.^177^ HPV16 E7 can also triggers autography by inhibiting dual‐specificity phosphatase 5.^178^ The expression of HPV oncoproteins can activate autophagy through different pathways, which fosters the survival of HPV‐infected cells. However, excessively heightened autophagy can induce apoptosis. From this perspective, autophagy is a double‐edged sword. For future research, the regulation of autophagy presents a novel avenue for the treatment of HPV‐related cervical lesions.

#### 2.3.3 Cell metastasis and invasion

The malignant transformation of histiocytes is characterized by diminished adhesion, heightened metastasis, and increased aggressiveness. The aggressiveness and metastasis of cells hinge upon matrix metalloproteinases (MMPs), specifically MMP‐2 and MMP‐9, which degrade extracellular matrix (ECM) components, dismantling the histological barricade to tumor cell metastasis. Furthermore, metastasis constitutes a fundamental trait of tumor cells, with numerous studies have highlighting the pivotal role of epithelial–mesenchymal transition (EMT) in tumor metastasis. HPV oncoproteins can degrade the ECM and trigger EMT, thus facilitating the metastasis and invasion of HPV positive cervical cancer cells.

During the process of invasion and metastasis, tumor cells must dismantle the ECM barrier and BM consisting of the intercellular matrix and BM. HPV‐associated lesions and malignancies exhibit alterations in the composition and function of the ECM.^179^ HPVE6 promotes the expression of ADAM10, which regulates cell adhesion and degrades intercellular substances, thereby facilitating the invasion and metastasis of cancer cells.^180^ MMPs are the enzymes most closely associated with tumor invasion and metastasis. HPV oncoproteins E2 and E7 can upregulate MMP1, MMP9, and MMP12 through the AKT, MEK/ERK, and AP‐1 pathways, as well as through direct protein–DNA interactions (which is reviewed in Mendonça et al.^181^).

EMT denotes the process by which epithelial cells transition phenotypically to acquire an interstitial cell phenotype under specific physiological or pathological conditions. The main morphological features of EMT include the loss of intercellular junction structures in epithelial cells, cytoskeletal remodeling resulting in a shift from polygonal to spindle‐shaped fibroblast‐like morphology, heightened cellular motility, and resistance to apoptosis. The principal molecular characteristics of EMT encompass the altered expression and depletion of epithelial markers like E‐cadherin and occludin, alongside the overexpression of mesenchymal cell markers such as N‐cadherin and vimentin. HPV oncoproteins, specifically E6 and E7, are capable of promoting the EMT.^182^ The loss of E‐cadherin expression represents a significant milestone in EMT. Downregulation of E‐cadherin expression occurs due to gene mutations, epigenetic gene silencing, and the binding of negatively regulated transcription factors to the CDH1 promoter (which encodes E‐cadherin). Several inhibitory proteins, namely, Snail1, Snail2, ZEB1, ZEB2, Twist1, and Twist2, contribute to the suppression of E‐cadherin. In HPV‐induced cervical cancer, E‐cadherin expression is downregulated, while Snail and ZEB1 are markedly upregulated.^183^ HR‐HPV E5/E7 is also implicated in this regulation.^184^, ^185^ Furthermore, HPV16/18 E7 curtails CDH1 and SNAI1 via DNA methylation.^185^ The assembly of the actin cytoskeleton is associated with HPV16 E6, rather than HPV18 E6. The downregulation of NHERF1 by HPV16E6 promotes cytoskeleton assembly and cell invasion.^186^ HPV16 E7 physically interacts with the actin‐binding protein gelsolin to regulate EMT via the HIPPO/YAP pathway.^187^ Additionally, RAS‐associated protein Rab31 is upregulated by HPV, further promoting EMT.^188^ As previously mentioned, the HPV E6 oncoprotein promotes telomerase activity through multiple mechanisms involving epigenetic, transcriptional, and posttranscriptional processes. In HFK/E6E7+ hTERT/hTER cells, a notable reduction in keratin and E‐cadherin levels is coupled with a significant elevation in Vimentin and N‐cadherin levels, providing evidence for the potential induction of EMT by hTERT/hTERC.^189^

Moreover, HPV viral oncoproteins have been discovered to promote EMT through the regulation of noncoding RNAs. HPV16 E6/E7 upregulates miR‐23‐3p, thus promoting EMT.^190^ Moreover, HPV16 E6/E7 upregulates lncRNA SNHG12, which can facilitate EMT through the ERK/Slug/E‐cadherin pathway. It has been observed that CircRNA_PVT1 can induce EMT,^191^ although its connection with HPV oncoproteins requires further exploration.

#### 2.3.4 HPV genome integration

HPV genes can instigate genome instability through multiple mechanisms, including cell cycle modulation,^192^, ^193^, ^194^, ^195^ interaction with DNA damage repair pathways that redirect high‐fidelity repair mechanisms to viral episomes rather than the host genome,^196^, ^197^ induction of DNA‐damaging oxidative stress, and modification of telomere length (which is reviewed in Porter and Marra^198^). HPV integration into the host genome results from heightened genome instability in HPV‐infected cells.^199^ The frequency of HPV integrations progressively rises during the progression of cervical lesions,^200^, ^201^, ^202^ rendering it a plausible biomarker for the surveillance of cervical cancer and precancerous disease conditions.^203^, ^204^, ^205^

HPV integration events transpire across all human chromosomes; however, a Chinese study unveiled an excessive concentration of these events on chromosome 19.^206^ Although HPV integration might seem stochastic, recent studies have pinpointed a growing array of HPV integration hotspot genes, including FHIT, LRP1B, PP1R37, HECW2, EMBP1, ANKRD50, SPTBN4, LINC00895, LYRM4‐AS1, LINC00374, RBFOX1, CSMD1, CDH13, KLHL4, KLF12, KLF5, CCDC106.^207^, ^208^, ^209^, ^210^ These genes may hold potential as targets for carcinogenesis and intervention. The type of viral integration depends on the HPV genotype, with HPV18 consistently undergoing integration.^211^ Insertion breakpoints exist in all gene regions of the HPV genome, with E1 and E2 being the most prevalent integration sites. HPV genome integration can manifest as a solitary copy or as multiple tandem repeats of the viral genome within the host genome. Two established models elucidate the integration mechanism. The first is the “looping” model, suggesting HPV integration is mediated by DNA replication and recombination.^212^ The second model is the “micro‐homologies mediated integration model”,^208^ highlighting two integration mechanisms: FoSTeS (fork stalling and template switching) and MMBIR (microhomology‐mediated BIR).The FoSTeS mechanism involves viral genome integration during replication fork stalling. Here, HPV utilizes this pathway and replaces the host genome template to integrate its own. In MMBIR, replication is induced by microhomologies‐mediated breaks, enabling HPV genome integration into the host genome during host DNA replication.^18^

HPV integration augments the expression of viral oncogenes. Initially, HPV integration disrupts the viral E2 gene, which encodes a transcriptional inhibitor of E6 and E7.^213^ Subsequently, productive integration culminates in the generation of stable viral‐cellular chimeric transcripts, which exhibit greater stability compared with those originating from episomal viral DNA. This depression of transcription and stabilization of fusion transcripts containing E6 and E7 synergistically intensify HPV oncogene expression.^214^ Integrated tumors frequently manifest elevated levels of E6 splicing, such as E6*I.^215^ Highly expressed HPV‐human fusion transcripts, such as HPV16 E6*I‐E7‐E1(SD880)‐human gene, drive cervical carcinogenesis, culminating in the overexpression of E6*I and E7.^216^ Furthermore, integration frequently arises in common fragile sites susceptible to tandem repeat formation, with flanking or interspersed host DNA often harboring transcriptional enhancer elements. When coamplified with the viral genome, these enhancers can form super enhancer‐like elements that drive robust viral oncogene expression. Even inadvertent integration events can foster viral oncogene expression.^217^, ^218^ As what we concluded before, HPV E6/E7 partakes in multiple processes, including cell cycle regulation, immune evasion, cell proliferation, and apoptosis. Its high expression confers selective growth advantages for cells,^219^ thereby sustaining cell transformation and promoting carcinogenic progression.

HPV integration can impact the host genome, leading to genome rearrangements, copy number variations, and gene mutations that can yield aberrant gene expression.^210^, ^220^, ^221^ These effects encompass amplified expression of cancer‐related proto‐oncogenes, such as oncogene amplification, and cis‐regulatory activation of genes through viral LCR,^222^ along with attenuated expression of TSGs, Additionally, HPV integration influences an array of genes linked to cancer progression, as indicated in Table 2.^223^ In addition to direct insertions between genes, HPV integration can also affect gene expression via long‐range or short‐range cis‐interactions, involving the expression of various tumor suppressors.^224^ Moreover, HPV integration can modify gene expression by altering DNA methylation and advancing carcinogenesis progression.^225^ While most studies suggest that HPV integration activates nearby oncogenes in cervical cancer, a recent study demonstrated that effective virus‐cell fusion transcripts typically do not produce endogenous human proteins or alter the expression of nearby genes.^214^ Subsequent research is essential for investigating the impact of HPV integration on genome structure and expression.

**TABLE 2:** Major genes related to cancer progression affected by HPV integration.

Author	Method	HPV type	HPV segment(s) involved	Human genes related	References
Brant, 2019	RNA‐sequencing	HPV16	E1	MCL1, PXMP4	^226^
			E2	NA	
			L2	NA	
		HPV18	E1	ALDH1A2, TP63, SLC16A14, PRKCH, HRH1, RAD51B, NRXN1	
			E2	PRKCH	
			E7	MMP12	
			L2	RAD51B	
Nkili‐Meyong, 2019	Whole genome sequencing	HPV16	NA	LINC01330, CACAT3, GANAB, AL162759.1, PDSS2, LINC00393, STK32A	^201^
		HPV18	NA	KLF12, RP4‐715N11.2, C20orf196, EP300	
		HPV58	NA	AC144450.1	
		HPV51	NA	NCKAP5	
		HPV33	NA	ADK	
Iden, 2021	Whole genome sequencing	HPV16	E1, L2	ERBB2	^227^
			E1, E2, E5, E7, L1, L2	RAD51B	
			E1	PVT1	
			E1, E2, L2	BNC1	
			E1	RSBN1	
			E2	USP36	
			E1	TAOK3	
Kamal, 2021	Polymerase chain reaction	HPV16, 52, 33	NA	MACROD2	^211^
		HPV16	NA	MIPOL1/TTC6	
		HPV16, 45, 58, 59, 73	NA	TP63	
Xiong, 2021	Fluorescence in situ hybridization	HPV16/18	NA	FHIT	^228^
			NA	TERT	
			NA	KLF5/KLF12	
			NA	MYC	
Wang, 2022	Whole genome sequencing	HPV16/18	NA	MYC	^222^
			NA	SOX	
			NA	NR4A	
			NA	ANKRD	
			NA	CEA	
Zhou, 2022	Long‐read sequencing	HPV16	L2, E2, E4	LINC00290/LINC02500	^229^
			E1, E6, L1	LENG9	
Karimzadeh, 2023	RNA sequencing	HPV16	NA	FOXA1, CUL2, KLF12, CD274	^220^
		HPV33	NA	SOX2	
		HPV58	NA	PBX1	
Li, 2023	Polymerase chain reaction	HPV16	NA	PIBF1	^230^
Zhao, 2023	RNA sequencing	HPV16	L1/L2	MACROD2	^231^

#### 2.3.5 Others

Besides the aforementioned cellular and molecular alterations that drive the malignant transformation of cervical cells following HPV infection, other elements including cellular metabolic reprogramming, oxidative stress, and angiogenesis in the tumor microenvironment significantly contribute to the process.

##### Cellular metabolism dysregulation

Throughout tumor growth, HPV‐infected cells undergo metabolic changes that support their malignant expansion.^232^ Researcher have demonstrated that HPV E6/E7 can elevate glycolysis.^233^ Interestingly, Kirschberg's study revealed that HPV8/16 E7 directly and the beta subunit of the mitochondrial ATP‐synthase (ATP5B), leading to decreased glycolytic activity but a significant increase in spare mitochondrial respiratory capacity.^234^ In HPV‐induced condylomata acuminata, HPV infection triggers the accumulation of glycogen and escalated glycogen metabolism through hypoxia‐inducible factor 1a, crucial for keratinocytes survival and proliferation.^235^, ^236^ HPV E6/E7 promotes aerobic glycolysis in cervical cancer by regulating IGF2BP2 to stabilize m6A‐MYC expression,^237^ inducing the expression of PKM2,^238^ and reducing poly‐ubiquitination to stabilize PGK1 protein.^239^ These processes yield additional energy sources for tumor proliferation and metastasis. Furthermore, during carcinogenesis or cancer persistence, other metabolic pathways, like glutamine metabolism, undergo modification. Researchers have discerned that E6/E7 exacerbates cell proliferation in a glutamine‐dependent manner,^240^ providing energy for cancer‐related processes.

##### Oxidative stress

Oxidative stress contributes to carcinoma, as an excess reactive oxygen species can induce oxidative harm to cellular structures and biomolecules, including proteins, lipids, and DNA break, fostering the integration of HPV virus DNA into host genome.^241^ Cells infected with HR‐HPVs can acclimate to oxidative stress by elevating the synthesis of endogenous antioxidants, including catalase, glutathione, and peroxiredoxin, which support the operation of viral oncoproteins.^242^

##### Angiogenesis

Angiogenesis profoundly influences the transition from dysplasia to aggressive cervical cancer. However, the link between HPV infection and angiogenesis remains uncertain. Qiu and colleagues^243^ proposed a novel mechanism for HPV16/18 E6‐induced cervical cancer progression, implicating “extracellular vesicles‐shuttled Wnt7b activating β‐catenin signaling,” meriting further exploration. In vitro experiments, E6 was found to activate vascular endothelial growth factor (VEGF)‐induced endothelial cell migration and exhibit potent proangiogenic activity.^244^

### 2.4 Contributing factors of cervical lesions

Not all HPV infections result in cervical cancer, and the progression of cervical lesions is influenced by various contributing factors, including the vaginal microenvironment and other factors.

#### 2.4.1 Vaginal microenvironment

The cervix resides within the vaginal microenvironment, and modifications in the vaginal microenvironment inevitably impact HPV‐related cervical lesions. The vaginal microenvironment encompasses the vaginal microbiome (VMB), the host endocrine system, vaginal anatomy, and local mucosal immunity. Cervicovaginal fluid is a crucial component of the vaginal microecology, containing mucins, antibodies, and various metabolites like lactic acid and sialidase (sld). The VMB, acting as the central element of the vaginal microenvironment, can be categorized into five distinct community state types (CSTs): CST I–III and CST V, predominantly comprising *Lactobacillus crispatus*, *Lactobacillus gasseri*, *Lactobacillus iners*, and *Lactobacillus jensenii*, respectively. CST IV delineates a heterogeneous group characterized by reduced abundance of *Lactobacillu* *s* species.^245^, ^246^ Normative vaginal microbial communities in females typically exhibit low biodiversity and are dominated by *Lactobacillus*. Elevated vaginal microbial diversity often closely correlates with female reproductive system disorders.^247^

Recently, a close relationship between vaginal microbiota composition and HPV infection has also been extensively documented.^16^, ^248^, ^249^ The VMB has been found correlated with the severity of cervical lesions. As cervical lesions advance, VMB diversity escalates,^250^, ^251^ accompanied by increased abundance of *Lactobacillus iners* and dysbiosis‐related bacteria such as *Porphyromonas*, *Prevotella*, *Bacteroides*, and *Anaerococcus* species predominating.^252^, ^253^, ^254^, ^255^ HPV infection can also adversely affect the VMB,^96^, ^256^ and these two factors synergistically drive cervical lesions progression. The potential mechanisms are summarized below. First, vaginal dysbiosis (BV) is identified as an independent risk factors for HR‐HPV infection.^257^ Proinflammatory response induced by vaginal dysbiosis‐associated bacteria in the vagina and several metabolites such as sld,^258^ vaginolysin,^259^ and the biogenic amines^260^ rupturing the epithelial mucus barrier, making it easier to invade the basal cells. Second, vaginal dysbiosis can influence the clearance and associated with persistent HPV infection. Dysbiosis is characterized by a decrease in *Lactobacillus* abundance and an overgrowth of species such as *Prevotella*, *Enterococcus durans*, *Dialister*, *Lachnospiraceae*, and *Porphyromonas uenonis*, which may be associated with persistent HPV infection.^261^, ^262^ *Lactobacillus crispatus* dominated VBM is beneficial for the clearance of HPV, while the effect of CST IV–BV with *Lactobacillus*. Spp. depletion and other anaerobic bacteria increased is conversed.^263^ Vaginal dysbiosis influences local mucosal immunity,^264^ induced the local immunosuppressive microenvironment,^265^, ^266^ and aiding the formation of biofilms,^267^, ^268^ hampering virus clearance and leading to persistent HPV infection.^269^ Among women with persistent HR‐HPV infection, an increase in the abundance of Prevotella may influence the development of persistent HPV infection and cervical lesions through host NFκB and C‐myc signaling.^270^ Last, vaginal dysbiosis might propel cervical lesions progression and malignant transformation through chronic inflammation induction and directly activating molecular pathways. Certain pathogenic bacteria associated with vaginal dysbiosis, such as *Gardnerella vaginalis* and *Megasphaera micronuciformis*, can increase the expression of E6 and E7, and coculture of SiHa cells with *Megasphaera micronuciformis* has been shown to decrease p53 and pRb levels and increase the percentage of cells in the S‐phase of the cell cycle.^271^ Phenylactic acid, a phenolic acid phytochemical predominantly produced by *L* *actobacillus*, represses E6/E7 expression while promoting cervical cancer cell migration and via IKK/NF‐κB‐mediated MMP‐9 activation.^272^ Additionally, coinfections in the vaginal microenvironment, such as Chlamydia trachomatis,^273^, ^274^ herpes simplex virus2, Ureaplasma urealyticum,^275^ can increase the risk of HPV‐associated cervical lesion and cancer; some of these coinfections influence the expression HPV oncogene.^276^ Researchers have found that the presence of specific anaerobic taxa, including *Megasphaera*, *P* *revotella timonensis*, and *Gardnerella vaginalis*, is associated with CIN2 persistence and slower regression,^277^ suggesting that VMB composition may serve as a useful biomarker for predicting disease outcome and tailoring surveillance in the future.

#### 2.4.2 Other risk factors

Other endogenous and exogenous contributing factors contribute to the progression of HPV‐associated cervical lesions and cervical cancer.^278^ Genetic factors determine the host's susceptibility to HPV,^279^, ^280^, ^281^ and immunogenetic variants assume a vital role in HR‐HPV infection and persistence.^282^, ^283^, ^284^ Smoking is a common risk factors of cervical cancer, the cigarette smoke components can enhance the expression of HPV16 E6/E7 via EGFR/PI3K/Akt/c‐Jun signaling pathway.^285^ Nicotine can promote HPV‐immortalized cervical epithelial proliferation by activating AKT/mTOR pathway, 4EBP1/eIF4E axis,^286^ and RPS27a/MDM2/P53 pathway.^287^ The cigarette smoke condensate can increase the YAP1 activity in human cervical epithelial cells.^130^ Hormone use has been reported involved in the physiopathology; the ERα, ERβ, PRLR expression levels are increased during the progression of cancer. In vitro, the 60 kDa PRL alone can significantly increase the proliferation of SiHa cells,^288^ 7β‑estradiol, prolactin interacts with HPV and can induce E6/E7 transcript.^289^ Estrogen also found can induce the genome instability in HR‐HPV‐infected cervix.^290^ Contraceptives like Depo‐medroxyprogesterone (DMPA) is also found increase the HPV infection, while it seems not accelerate disease progression.^291^ Immune deficiency, such as the human immunodeficiency virus‐infected, seems more likely to have HR‐HPV infection than uninfected, attribute to the long‐standing immune deficiency.^292^, ^293^ Polycyclic aromatic hydrocarbons are also considered as a cofactor in HPV‐mediated carcinogenesis, while the mechanism is worthy further study.^294^ Interestingly, researchers also found that the chronic stress and diurnal cortisol also play an important role in HR‐HPV infection and HPV‐associated cervical carcinogenesis.^295^ Furthermore, dietary factors also appear to play an important role in the animal experiment and human clinical research and both show that diets with low antioxidants (vitamin A, B2, E, and folate) are more likely to develop HPV infections.^296^, ^297^

## 3 THERAPEUTIC INTERVENTIONS

**FIGURE 3:**
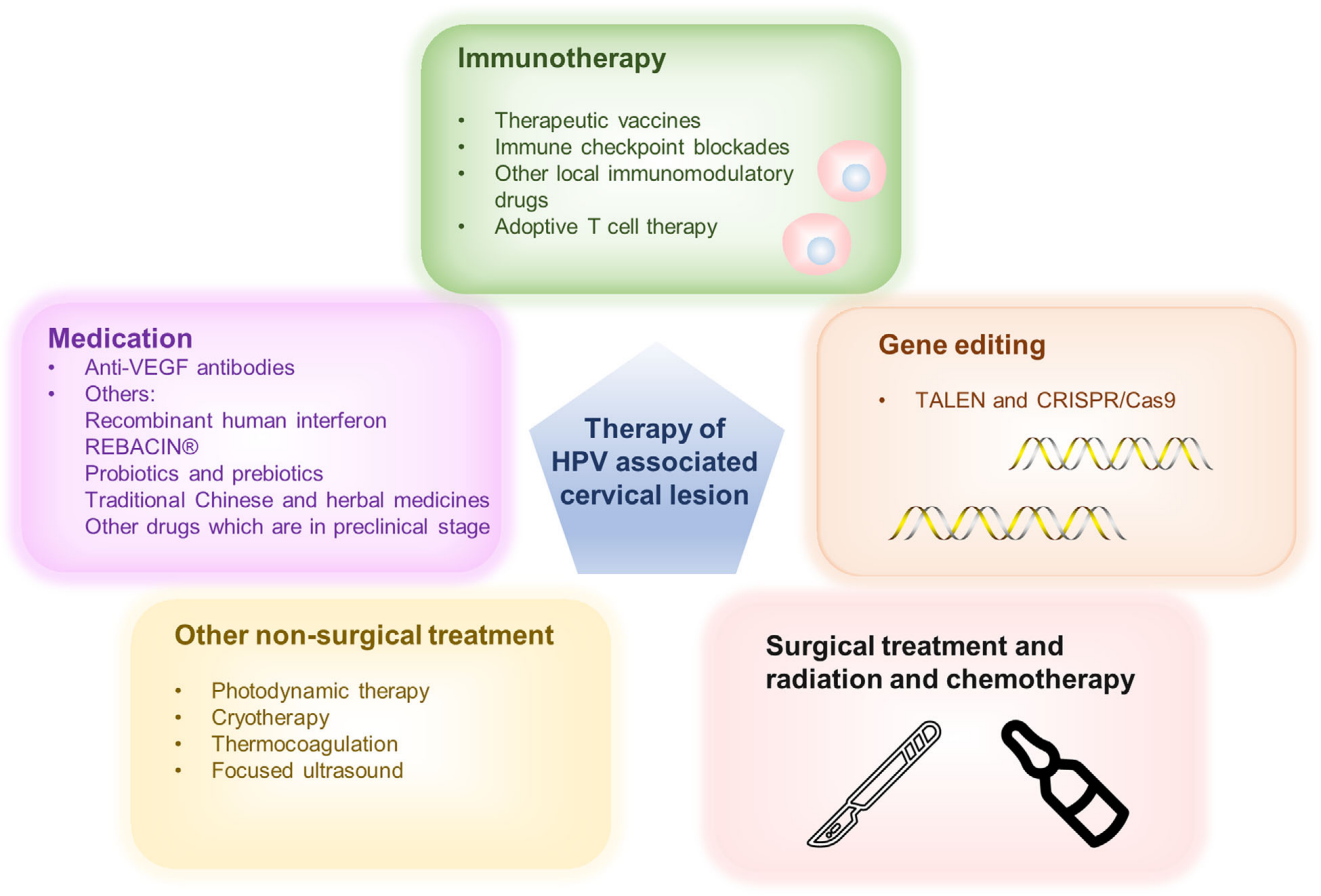
Therapeutic interventions of HPV‐associated cervical lesion. Traditional therapy of HPV‐associated cervical lesions is surgical treatment combined with radiation or chemotherapy. Up to now, there is no specific drug available for HPV infection. Immunotherapy and gene editing technology is holding the potential to enable precise therapy for cervical cancer in the future. Other nonsurgical treatments, such as photodynamic therapy, cryotherapy, thermocoagulation, and focused ultrasound, are commonly used for cervical precancerous lesions.

Currently, the management of precancerous lesions caused by carcinogenic HPV primarily involves surgical interventions, including laser treatment, loop electrosurgical excision procedure, and cold knife conization. Treatment selection hinges upon lesion severity, HPV type, and the patient's condition. However, some patients continue to experience persistent HPV infection postsurgery, heightening the risk of subsequent recurrence.^298^ Unfortunately, effective treatment drugs for persistent HPV infection are still absent. The primary treatment for cervical cancer entails surgery combined with radiation and chemotherapy. However, women afflicted with positive lymph nodes, recurrent cancer, or metastatic cervical cancer still have a grim prognosis, accentuating the demand for innovative treatment strategies. The following section provides a summary of current treatments and emerging approaches for the management of HPV infection, HPV‐related precancerous lesions, and cervical cancer. This includes HPV therapeutic vaccines and genome editing techniques. The primary objectives of this review centers on treatment, with an emphasis on enhancing clinical management for affected patients (Figure 3).

### 3.1 Immunotherapy

The widespread implementation of the HPV vaccine has resulted in a reduction in the prevalence of HPV‐associated cervical lesions. However, the vaccine seems to have limited efficacy in treating established HPV infections and cervical lesions. To combat persistent HPV infections and preexisting HPV‐related cervical lesions, researchers are developing therapeutic vaccines. Additionally, ongoing clinical trials are exploring immunotherapy drugs such as anti‐PD‐1/LI antibodies and novel approaches like adoptive cell therapy, which aim to clear HPV infections and inhibit cervical lesions by enhancing the innate and adaptive immune responses. These approaches, when combined with standard‐of‐care treatments, may offer new treatment options.

#### 3.1.1 Therapeutic vaccines

Unlike preventive vaccines, therapeutic vaccines aim to stimulate the production of CD8+ cytotoxic and CD4+ helper T cell responses. These vaccines mostly target E6/E7 proteins, although some vaccines used in cancer research attempt to target other proteins such as p16 and telomerase (NCT 01462838 and NCT 03946358). The various types of therapeutic vaccines are as follows.

##### Vectors‐based vaccines

*Viral vectors‐based vaccines*: Viral vector‐based vaccines utilize adenovirus, adeno‐associated virus, alphavirus, lentiviruses, vesicular stomatitis virus, and vaccinia viruses to deliver HPVE6 and E7.^299^, ^300^, ^301^, ^302^ The first vaccinia‐based vaccine used in early cervical cancer is TA‐HPV, which demonstrated potent antigen‐directed antibody and cytotoxic responses in a completed phase II clinical trial (NCT00002916). Another vaccine, Tipapkinogen Sovacivec, based on modified vaccinia virus Ankara, exhibited efficacy in patients with CIN/III (NCT01022346). Vvax001, an alphavirus‐based vaccine,^303^ elicited a vigorous immune response in CIN patients, supporting further clinical evaluation.^304^ Ongoing clinical trials encompass virous virus‐based vaccines, as summarized in Table 3. Additionally, other viral vectors such as lymphocytic choriomeningitis virus^305^ and cytomegalovirus^306^, ^307^ are still undergoing preclinical studies and hold promise as potential therapeutic vaccines in the future. However, the generation of antibacterial or antiviral immune responses, as well as the presence of neutralizing antibodies, can pose challenges to the efficacy of viral vector‐based vaccines and must be addressed in future research.

**TABLE 3:** Therapeutic vaccines for the treatment of HPV‐related cervical precancerous lesions and cervical cancer in clinical trials.

Disease	Start date	Number	Intervention	Route	Vaccine type	Ag	status	phase	Outcome (OR), mPFS (mon.), mOS (mon.), CR, PR	Sources
Bacterium‐based vaccines										
HPV+ cervical cancer	2015.1	25	ADXS11‐001	NA	Listeria monocytogenes vaccine	E7	Completed	I/II	NA	NCT02164461
Platinum‐refractory cervical cancer	2018.10	50	ADXS‐HPV	Intravenous injection	Listeria monocytogenes vaccine	E7	Completed	II	ORR: 6%; mPFS: 2.8; mOS: 6.1; CR: 2%; PR: 4%	NCT01266460^340^
CINII	2014.2	40	1. GLBL101c 2. Placebo	Oral	Lactobacillus‐based vaccine	E7	Completed	IIB	CR:11%, PR:11% CR:0, PR:37%	NCT02195089^311^
CINIII	2014.3	19	BLS‐M07	Oral	Lactobacillus casei‐based vaccine	E7	Completed	I/2a	NA	^341^
Healthy volunteer	2016.10	55	NZ8123‐HPV16‐optiE7	Oral	Lactococcus lactis‐based vaccine	E7	Completed	I	NA	^342^
Healthy volunteer	2018.6	69	NZ8123HPV16‐optiE6	Oral	Lactococcus lactis‐based vaccine	E6	Completed	I	NA	^310^
Virus‐based vaccines										
Early cervical cancer	1996.11	44	TA‐HPV	NA	Vaccinia‐based vaccine	E6/E7	Completed	II	NA	NCT00002916
CINII/III	2009.8	192	1. Tipapkinogen Sovacivec 2. placebo	Subcutaneous injection	Modified vaccinia virus Ankara (MVA)‐based vaccine	E6/E7	Completed	II	1. CR: 24%, PR: 11.6% 2. CR: 9.5, PR: 11.1%	NCT01022346^343^
HPV+ epithelial cancer	2019.11	200	HB‐201/HB202 + pembrolizumab	Intravenous injection	Arenavirus‐based vaccine	E6/E7	Recruiting	I/II	NA	NCT04180215
HPV‐associated cancer	2020.8	20	PRGN‐2009 alone + anti‐PDL1/TGF‐Beta Trap (M7824)	NA	Adenovirus	E6/E7	Active, not recruiting	I/II	NA	NCT04432597
CIN	NA	12	Vvax001	Intramuscular	Alphavirus‐based vaccines	E6/E7	Completed	I	NA	^304^
Peptide vaccines										
Advanced HPV and P16INK4a positive cancers	2011.8	26	P16_37‐63 peptide with Montanide ISA‐51	Subcutaneous injection	Peptide vaccine	P16	Completed	I/II	NA	NCT01462838^344^
HPV 16+ advanced or recurrent cervical cancer	2013.9	93	ISA101/ISA101b + chemotherapy(C+P)	NA	Peptide vaccine	E6/E7	Completed	II	NA	NCT02128126
HSIL	2015.11	125	PepCan	Intradermal injection	Peptide vaccine	E6	Completed	II	NA	NCT02481414
HPV16+ recurrent cervical cancer	2015.12	24	ISA101 + ICI (nivolumab)	NA	Peptide vaccine	E6/E7	Completed	II	mPFS:2.66; mOS:15.3	^345^
HPV16+ (pre‐)malignant lesion	2016,7	25	Hespecta	Intradermal	Peptide vaccine	E6	Completed	I	NA	^346^
Cervical, anal, and oropharyngeal cancer (HLA‐A2 +)	2016.12	11	DPX‐E7	NA	Peptide vaccine	E7	Active, not recruiting	I/II	NA	NCT02865135
HPV+‐related cancer(including cervical cancer)	2020.2	47	UCPVax + anti‐PD‐1 (Atezolizumab)	Intradermal	Peptide vaccine	telomerase	Active, not recruiting	II	NA	NCT03946358
Advanced HPV‐associated cancer	2020.6	51	PDS0101 + immune modulators (M7824+NHS‐IL12)	NA	Peptide vaccine	NA	Active, not recruiting	I/II	NA	NCT04287868
Advancer solid tumors (including cervical cancer)	2022.3	36	Neoantigen peptide vaccine + pembrolizumab	NA	Peptide vaccine	NA	Recruiting	I	NA	NCT05269381
Protein‐based vaccines										
HSIL	2015.11	10	TVGV‐1 vs. GPI‐0100	NA	Protein‐based vaccine	E7	Unknow	II	NA	NCT02576561
HPV16+ CINIII	2004.6	84	HSP‐E7	Subcutaneous injection	Protein‐based vaccine	E7	Completed	II	NA	NCT00054041
HPV16/18‐associated infections	2013.11	239	ProCervix (GTL001) + imiquimod	NA	Protein‐based vaccine	E7	Completed	II	NA	NCT01957878
HSIL	2015.11	10	TVGV‐1 vs. GPI‐0100	NA	Protein‐based vaccine	E7	Unknow	II	NA	NCT02576561
HPV 16‐associated cervical cancer	2019.4	14	TA‐CIN	Intramuscular	Protein‐based vaccine	L2/E6/E7	Active, not recruiting	I	NA	NCT02405221
Stage IB3‐IVA cervical cancer	2020.10	35	PDS0101 + chemoradiotherapy	NA	Protein‐based vaccine	E6/E7	Recruiting	II	NA	NCT04580771
DNA vaccines										
Recurrent/metastatic, treatment‐refractory HPV‐16/18 cervical cancer, or rare HPV‐associated (anal and penile) cancers	NA	21	MEDI0457 + durvalumab	NA	DNA vaccine	NA	Completed	II	ORR: 10%, mPFS: 4.6, mOS: 7.6	^312^
HPV16+‐related Cervical cancer and Precancer	2005,7	16	pNGVL4a‐Sig/E7(detox)/HSP70	Intramuscular	DNA vaccine	E7	Completed	I/II	NA	NCT00121173^347^
HPV‐related cervical cancer and precancer	2008.11	75	1. TA‐HPV 2. pNGVL4a‐Sig/E7 (detox)/HSP70DNA vaccine 3. imiquimod	Intramuscular	DNA vaccine	E7	Recruiting	I	NA	NCT00788164
CINII‐III	2011.4	167	VGX‐3100	EP	DNA vaccine	E6/E7	Completed	II	NA	NCT01304524
Cervical cancer after Chemoradiation	2014.6	10	MEDI0457 (INO‐3112)	intramuscularly by EP	DNA vaccine	E6/E7	Completed	I	ORR:10%	^348^
CINIII	2014.7	72	GX‐188E	Intramuscularly by EP	DNA vaccine	E6/E7	Completed	II	NA	NCT02139267
CIN	2015.8	134	GX‐188E	Intramuscularly by EP	DNA vaccine	E6/E7	Unknown	II	NA	NCT02596243
HPV16+ HSIL, CIN2/3	2015.8	34	VB10.16	Intramuscular	DNA vaccine	NA	NA	NA	NA	NCT02529930
HPV‐related cancer	2017.4	44	BNT113	Intradermal	RNA vaccine	N	Suspended	I/II	NA	NCT03418480
HPV16/18+ CIN	2017.6	201	VGX‐3100	Intramuscularly by EP	DNA vaccine	E6/E7	Completed	III	NA	NCT03185013
HPV16/18+ advanced cervical cancer	2018.6	36	GX‐188E + anti‐PD‐1 (pembrolizumab)	Intramuscular	DNA vaccine	E6/E7	Completed	II	ORR: 42%; CR: 15%	NCT03444376^349^
ASC‐US, LSIL, HSIL	2019.3	134	pNGVL4a‐Sig/E7(detox)/HSP70 DNA+TA‐CIN	Intramuscular	DNA vaccine	E7	Recruiting	II	NA	NCT03911076
CIN	2019.4	203	VGX‐3100	Intramuscularly by EP	DNA vaccine	E6/E7	Completed	III	NA	NCT03721978
HPV16/18+ HSIL	2022.3	12	NWRD08	Intramuscularly by EP	DNA vaccine	NA	Recruiting	NA	NA	NCT05905354
Whole cell‐based vaccines										
HPV16/18+ recurrent cervical cancer	2016.10	11	BVAC‐C	Intravenous injection	B cell and monocyte‐based vaccine	E6/E7	Completed	I/II	mPFS:6.8	NCT02866006^324^
HPV 16 /18+ cervical cancer failure to first‐line platinum‐based chemotherapy	2021,6	37	BVAC‐C + durvalumab	Intravenous injection	B cell and monocyte‐based vaccine	E6/E7	Not yet recruiting	II	NA	NCT04800978
CINI‐II	2019.4	80	DC vaccine	NA	DC vaccine	E6/E7	Unknown	I	NA	NCT03870113
Advanced cervical cancer	2021.10	12	RT201	Intravenous administration	Macrophage‐based vaccine	Tumor antigen	Recruiting	NA	NA	NCT05930301
HPV16+ recurrent, locally advanced or metastatic solid tumors (including cervical cancer)	2022.3	60	SQZ‐eAPC‐HPV + pembrolizumab	NA	Engineered mononuclear cells‐based vaccine	NA	Recruiting	I/II	NA	NCT05357898
HPV16+ recurrent, locally advanced or metastatic solid tumors (including cervical cancer)	2021.8	72	SQZ‐AAC‐HPV + ipilimumab/nivolumab	NA	Engineered red cells‐based vaccine	N	Recruiting	I	NA	NCT04892043

*Data sources*: clinical registration website (https://clinicaltrials.gov), excluding the withdrawal and terminal trials, as of July 2023. NA, data are missing or not publicly available or applicable; Ag, antigen; ORR, objective response rate; mPFS, median progression‐free survival; mOS, median overall survival; CR, complete response; PR, partial response; EP, electroporation; C+P, carboplatin and paclitaxel.

*Bacterial vectors‐based vaccines*: Listeria and Lactobacillus are the most widely studied vectors.^308^, ^309^, ^310^ Listeria‐based vaccines ADXS11‐001 have undergone phase I/II clinical to assess their efficacy and safety in HPV+ cervical cancer (NCT02164461 and NCT01266460). A phase III clinical trial for advanced cervical cancer (NCT02853604) was initiated but unfortunately had to be terminated for commercial reasons. Another widely investigated type of vaccine is the lactic acid flora‐based vaccine, mostly administered orally for ease of use. Phase I and II clinical studies have been conducted in CINII^311^ and CINIII (NCT02195089), but no phase III studies have been carried out.

##### Peptide/protein‐based vaccines

*Peptide‐based vaccines*: Peptide‐based vaccines are considered safe, stable, and easy to produce. ISA101, consists of HPV‐16 E6/E7 synthetic long peptide, has completed phase II clinical trials in recurrent cervical cancer (NCT02128126 and NCT02426892). Most therapeutic vaccines target E6/E7 proteins. UCPVax, a therapeutic cancer vaccine formulated from distinct peptides originating from telomerase (hTERT, human telomerase reverse transcriptase), has shown safety and potent immunogenic in non‐small cell lung cancer and is currently being evaluated in ongoing clinical trials for HPV‐related cancer, including cervical cancer (NCT03946358).^312^ Other ongoing clinical trials for peptide‐based vaccines are summarized in Table 3. A limitation of peptide‐based vaccines is their suboptimal immunogenicity, prompting the requirement for adjuvants to augment their efficacy. Adjuvants such as CpG oligodeoxynucleotides,^313^, ^314^ cobalt‐porphyrin‐phospholipid,^315^ and manganese (Mn4+)‐doped silica nanoparticles (Mn4+‐SNPs)^316^ have been found to enhance immune responses.

Protein‐based vaccines primarily utilize E6/E7 proteins as the main source of antigens. One advantage is that these vaccines contain all epitopes of HLA. However, a potential drawback is their tendency to induce antibody responses rather than CTL responses. Enhancing MHC I presentation is an important area of research, and the utilization of adjuvants and immunostimulant compound such as CpG and GPI0100 could contribute to accomplishing this objective.^317^ Clinical trials assessing GTL001 (ProCervix) in HPV‐infected patients have yielded unsatisfactory results (NCT01957878), while other studies are still ongoing, as summarized in Table 3.

##### Nucleic acid‐based vaccines

*DNA‐based vaccines*: Nucleic acid‐based vaccines are safe and easy to manufacture. The DNA‐based vaccines have been extensively studied in clinical trials.^318^ These vaccines entail the introduction of plasmid DNA encoding a desired protein into the host's tissue, enabling its expression and synthesis. However, nucleic acid‐based vaccines have limited immunoprototypes, which curtails their utility. Nonetheless, preclinical studies have demonstrated that the incorporation of adjuvants such as MDA‐7/IL‐24/IL‐7/GM‐CSF, immune checkpoint blockers, and chitosan nanoparticles enhances the functionality of DNA vaccines.^319^, ^320^, ^321^, ^322^ A noteworthy example of therapeutic DNA‐based vaccines is VGX3100, which comprises two DNA plasmids encoding HPV16/18 E6/E7 proteins. Phase III clinical trials of VGX3100 in CIN II‐III have been completed (NCT03185013 and NCT03721978), although the results have not been published yet. These findings offer hope for patients seeking conservative treatments. Other DNA‐based vaccines, including GX‐188E, NWRD08, and VB10.16,^323^ are currently undergoing clinical trials, as summarized in Table 3, with the goal of future clinical implementation.

*RNA‐based vaccines*: RNA‐based vaccines have a higher probability of successful transfection compared with DNA‐based vaccines because they only need to cross the plasma membrane. However, due to their inherent instability, their application in HPV‐related conditions is restricted. Currently, the only clinical trial evaluating an RNA‐based vaccine in HPV‐related cancer is BNT113 (NCT03418480).

##### Whole cell‐based vaccines

Whole cell‐based vaccines entail the loading of HPV antigens into the patient's APCs and reintroducing them into the patient's body. Currently, various types of cells, including B cells, monocytes, DCs, macrophages, engineered mononuclear cells, and engineered red cells, have been used as vectors (NCT02866006, NCT04800978, NCT03870113, NCT05930301, NCT05357898, and NCT04892043). Completed phase I clinical trials have demonstrated the safety and effectiveness of BVAC‐C, a vaccine based on B cells and monocytes.^324^ Nonetheless, the personalized nature and the obstacles related to large‐scale manufacturing restrict the applicability of whole cell‐based vaccines in the realm of HPV vaccine advancement.

#### 3.1.2 Regulates the immune environment

As previously discussed, HPV infection hampers innate and adaptive immunity through various mechanisms, leading to an immunosuppressive environment that impairs the activity of immune cells. HPV‐infected cells can evade immune surveillance and contribute to the progression of cervical lesions and cervical cancer. Thus, reversing the immunosuppressive status has emerged as a crucial therapeutic goal. (Clinical trials about immunotherapy drugs for cervical cancer is summarized in Table 4.)

**TABLE 4:** Clinical trials related to immunotherapy drugs for cervical cancer.

Disease	Start date	Number	Intervention	Mechanism	Status	Phase	Sources
Ovarian, colorectal, breast, renal, or cervical cancer patients	2013.11	104	Durvalumab	Anti‐PD‐1 antibody	Completed	I	NCT01975831
			Tremelimumab	Anti‐CTLA‐4 antibody			
Solid tumor (including cervical cancer)	2014.2	477	Pembrolizumab	Anti‐PD‐1 antibody	Completed	I	NCT02054806^350^
Cervical adenocarcinoma	2015.5	26	Nivolumab	Anti‐PD‐1 antibody	Active, not recruiting	II	NCT02257528^351^
Cervical adenosquamous carcinoma							
Recurrent cervical carcinoma							
Advanced cancer patients (including cervical cancer)	2015.10	578	Nivolumab	Anti‐PD‐1 antibody	Completed	I/II	NCT02488759^352^
			Ipilimumab	Anti‐CTLA‐4 antibody			
			Relatlimab	Anti‐LAG3 antibody			
			Daratumumab	Anti‐CD38 antibody			
Advanced solid tumor patients (including cervical cancer)	2015.11	1609	Pembrolizumab	Anti‐PD‐1 antibody	Recruiting		NA
Advanced/metastatic solid tumors (including cervical cancer)	2016.9	260	INCB001158	Arginase inhibitor	Completed	I/II	NCT02903914
			Pembrolizumab	Anti‐PD‐1 antibody			
Advanced, measurable, biopsy‐accessible cancers, including cervical	2016.11	58	Durvalumab	Anti‐PD‐L1 antibody	Completed	I/II	NCT02643303
			Tremelimumab	Anti‐CTLA‐4 antibody			
			PolyICLC	TLR3 agonist			
Solid tumor patients (including HPV + cervical cancer patients)	2017.3	137	Atezolizumab	Anti‐PD‐L1 antibody	Active, not recruiting	II	NCT03074513
			Bevacizumab	Anti‐VEGF antibody			
Advanced malignancies	2017.4	145	INCAGN10876	GITR agonist	Completed	I/II	NCT03126110
Metastatic cancer, including cervical cancer			Ipilimumab	Aanti‐CTLA‐4/			
			Nivolumab	Anti‐PD‐1 antibody			
Recurrent, persistent, or metastatic cervical cancer patients	2017.3	11	Atezolizumab	Anti‐PD‐L1 antibody	Completed	II	NCT02921269^353^
			Bevacizumab	Anti‐VEGF antibody			
Refractory or persistent endometrial, cervical, or uterine cancer patients	2017.7	43	Pembrolizumab + immune modulatory cocktail	Anti‐PD‐1 antibody	Completed	II	NCT03192059
Advanced malignancies (including cervical cancer)	2017.10	52	INCAGN01949	OX40 agonist	Completed	I/II	NCT03241173
			Ipilimumab	Anti‐CTLA‐4/			
			Nivolumab	Anti‐PD‐1 antibody			
Advanced cancer (including cervical cancer)	2018.4	378	Ciforadenant	Anti‐adenosine 2A receptor antibody	Active, not recruiting	I	NCT03454451
			CPI‐006	CD73 inhibitor			
			Pembrolizumab	Anti‐PD‐1 antibody			
HPV‐associated cancer	2018.2	57	M7824	Anti‐PD‐L1/TGF antibody	Completed	II	NCT03427411^354^
Advanced cancers patients (including cervical cancer)	2018.4	378	CPI‐006	CD73 inhibitor	Active, not recruiting	I	NCT03454451
			Ciforadenant	Antiadenosine 2A receptor antibody			
			Pembrolizumab	Anti‐PD‐1 antibody			
Advanced cancer patients, including cervical	2018.6	22	INCAGN02385	Anti‐LAG3 antibody	Completed	I	NCT03538028
Recurrent, high grade or metastatic female reproductive cancer	2018.9	48	Ipilimumab	Anti‐CTLA‐4	Active, not recruiting	I	NCT03508570
			Ipilimumab	Anti‐PD‐1 antibody			
Advanced cancer patients, including cervical	2018.9	40	INCAGN02390	Anti‐TIM3 antibody	Completed	I	NCT03652077
Solid tumor patients (including cervical cancer)	2018.11	264	mRNA‐2752	OX40 agonist	Recruiting	I	NCT03739931
			Durvalumab	Anti‐PD‐1 antibody			
Advanced solid tumors	2019.1	120	ASP1951	GITR agonist	Active, not recruiting	I	NCT03799003
			Pembrolizumab	Anti‐PD‐1 antibody			
Advanced solid tumor or lymphoma patients (including cervical cancer)	2019.3	87	SL‐279252	Bispecific anti‐PD‐1 Antibody and OX40 agonist	Active, not recruiting	I	NCT03894618
Advanced solid tumors patients (including cervical cancer)	2019.5	78	XmAb22841	Bifunctional CTLA‐4 inhibitor and LAG3 inhibitor	Completed	I	NCT03849469
			Pembrolizumab (PD‐1 inhibitor)	PD‐1 inhibitor			
Locally advanced cervical cancer	2019.6	132	TSR‐042	Anti‐PD‐1 antibody	Recruiting	II	NCT03833479
Solid tumor (including cervical cancer)	2019.11	333	INBRX‐106 Pembrolizumab	OX40 agonist Anti‐PD‐1 antibody	Recruiting	I	NCT04198766
Advanced and/or metastatic solid tumors (including cervical cancer)	2019.11	320	RO7247669	Anti‐PD‐1 and anti‐LAG3 bispecific inhibitory antibody	Recruiting	I/II	NCT04140500
Solid tumor, including cervical cancer	2020.3	185	TTX‐030 Budigalimab	CD39 enzymatic inhibitor Anti‐PD‐1 antibody	Active, not recruiting	I	NCT04306900
Cervical cancer	2020.7	40	Ipilimumab nivolumab	Anti‐CTLA‐4/ Anti‐PD‐1 antibody	Active, not recruiting	NA	NCT04256213
Recurrent, metastatic cervical cancer patients	2020.7	30	AK104	Bispecific anti‐PD‐1/CTLA‐4 antibody	Completed	II	NCT04380805
Cervical, vaginal, and vulval inflammatory diseases	2020.10	45	Pembrolizumab	Anti‐PD‐1 antibody	Recruiting	II	NCT04211103
Cervical cancer patients	2020.10	25	M7824 Bevacizumab	Drug that binds PD‐L1 and neutralizes TGFβ anti‐VEGF antibody	Completed	I	NCT04551950
Locally advanced cervical cancer	2021.1	45	Pembrolizumab	Anti‐PD‐1 antibody	Recruiting	II	NCT04238988
Recurrent cervical cancer	2021.1	28	Pembrolizumab Olaparib	Anti‐PD‐1 antibody PARP inhibitor	Active, not recruiting	II	NCT04641728
PD1‐high mRNA Expressing Tumors (including cervical cancer)	2021.4	184	Spartalizumab Tislelizumab	Anti‐PD‐1 antibody	Recruiting	II	NCT04802876
HPV 16/18+ cervical cancer failure to first‐line platinum‐based chemotherapy	2021.6	37	BVAC‐C + Durvalumab	B cell and monocyte‐based vaccine Anti‐PD‐1 antibody	Not yet recruiting	II	NCT04800978
Cervical intraepithelial neoplasia (including carcinoma in situ)	2021.6	25	Pembrolizumab	Anti‐PD‐1 antibody	Recruiting	II	NCT04712851
Solid tumors	2021.6	561	XB002 Nivolumab Bevacizumab	TF‐ADC Anti‐PD‐1 antibody	Recruiting	I	NCT04925284
Advanced cervical cancer	2021.7	172	Camrelizumab Apatinib Bevacizumab	Anti‐PD‐1 antibody Anti‐VEGF antibody	Recruiting	II	NCT04974944
Advanced solid tumor (including cervical cancer)	2021.8	115	MDNA11 Pembrolizumab	IL‐2 Super Factor	Recruiting	I/II	NCT05086692
Advanced solid tumors (including cervical cancer)	2021.11	100	Ipilimumab Pembrolizumab Durvalumab	Anti‐CTLA‐4 antibody Anti‐PD‐1 antibody	Recruiting	I/II	NCT05187338
Recurrent cervical cancer	2022.3	20	NA	Anti‐PD‐1 antibody	Recruiting	NA	NCT05310305
Recurrent cervical cancer	2022.3	58	Tislelizumab plus Radiotherapy	Anti‐PD‐1 antibody	Recruiting	II	NCT05310383
Advanced unresectable or metastatic solid tumors (including cervical cancer)	2022.10	131	NC410 Pembrolizumab	LAIR‐1 agonist Anti‐PD‐1 antibody	Recruiting	I/II	NCT05572684
Locally advanced cervical cancer	2022.11	30	Tislelizumab	Anti‐PD‐1 antibody	Recruiting	II	NCT05588219
Cervical cancer	2023.3	118	Serplulimab	Anti‐PD‐1 antibody	Recruiting	NA	NCT05883670
Recurrent or metastatic cervical cancer	2023.6	58	Cadonilimab Nab paclitaxel	Anti‐CTLA‐4 and PD‐1 antibody Chemotherapy drugs	Not yet recruiting	II	NCT05824494
Advanced cervical cancer	2023.6	33	Zimberelimab lenvatinib	Anti‐PD‐1 antibody Anti‐VEGF antibody	Not yet recruiting	II	NCT05824468
Locally advanced cervical cancer	2023.5	36	Envafolimab	Anti‐PD‐1 antibody	Not yet recruiting	II	NCT05799469
Advanced solid tumors (including cervical cancer)	2022.3	36	Neoantigen peptide vaccine Pembrolizumab	Therapy vaccine Anti‐PD‐1 antibody	Recruiting	I	NCT05269381
IB2‐IIIB cervical cancer	2022.3	92	Camrelizumab + chemoradiotherapy	Anti‐PD‐1 antibody	Recruiting	II	NCT05311566
Advanced or metastatic solid tumors	2022.3	79	NGM831 pembrolizumab	Anti ILT3 antibody Anti‐PD‐1 antibody	Recruiting	I	NCT05215574
Advanced or metastatic solid tumors (including cervical cancer)	2022.5	71	NGM438 Pembrolizumab	LAIR1 antagonist antibody Anti‐PD‐1 antibody	Recruiting	I	NCT05311618
Cervical cancer	2022.8	112	Nivolumab Ipilimumab Chemoradiation	Anti‐PD‐1 antibody Anti‐CTLA‐4 antibody	Recruiting	II	NCT05492123
Recurrent cervical cancer	2022.3	122	Camrelizumab Albumin‐bound Paclitaxel	Anti‐PD‐1 antibody	Recruiting	II	NCT05290935
Advanced cervical cancer	2022.10	35	Pembrolizumab Lenvatinib	Anti‐PD‐1 antibody Anti‐VEGF antibody	Recruiting	II	NCT04865887
Advanced solid tumors (including cervical cancer)	2023.1	120	Q702 Pembrolizumab	Axl/Mer/CSF1R Selective Tyrosine Kinase Inhibitor Anti‐PD‐1 antibody	Recruiting	I/II	NCT05438420
Advanced solid tumors (including cervical cancer)	2022.3	170	HFB200301 Tislelizumab	TNFR2 agonist antibody Anti‐PD‐1 antibody	Recruiting	I	NCT05238883
Advanced or metastatic cancers	2023.3	90	CyPep‐1 Pembrolizumab	Tumor specific, cytotoxic peptide Anti‐PD‐1 antibody	Recruiting	I/II	NCT05383170

*Data sources*: clinical registration website (https://clinicaltrials.gov), excluding the withdrawal and terminal trials, as of July 2023. NA, data are missing or not publicly available or applicable.

##### Immune checkpoint blockades

The programmed death‐ligand 1 (PD‐L1)/PD‐1 axis is crucial for maintaining autoimmune tolerance. PD‐L1 functions as the ligand for PD‐1, and their interaction leads to the deactivation of T cells. Frequently, tumor cells exhibit elevated PD‐L1 expression levels. As previously explained, HPV E6/E7 oncoproteins can upregulate PD‐1 expression. Blocking PD‐1 has shown promise in HPV‐induced cervical cancer.^325^ In 2018, the United States Food and Drug Administration (US FDA) approved pembrolizumab, an anti‐PD‐1 antibody, for the treatment of cervical cancer. The clinical trial NCT04712851 is currently assessing the use of pembrolizumab in CIN. Other anti‐PD‐1 antibodies, including Durvalumab and Nivolumab, remain under evaluation in clinical trials. Another important immune target is CTLA‐4, which is expressed on the surface of T cells, which diminishes T cell cytotoxicity upon binding to CD80 or CD86 on APCs. Inhibitors of CTLA‐4, such as ipilimumab and tremelimumab, have been extensively investigation in clinical trials, and completed phase I/II trials have shown potent immune activation in metastatic/recurrent cervical cancer. Additionally, other immune checkpoints, including OX40, GITR, TLR3, TIGIT, LAG3, TIM3, CD39, and A2AR,^326^, ^327^ as well as relevant agonist and antagonist antibodies, have been investigated in cervical cancer. However, none of them have been approved for use in the context of cervical cancer. Recent preclinical studies have identified CD96 as a potential therapeutic target. Blocking CD96 enhances the efficacy of PD‐1 blockade and improves the function of CD8+ T cells, providing a new avenue for research.^328^ Combining immune inhibitors that target TIGIT, PD‐L1, and TGF‐β has demonstrated significant antitumor efficacy in preclinical models,^329^ and clinical trials investigating this combination are ongoing, indicating a promising direction for research.

##### Other local immunomodulatory drugs

In addition to the aforementioned targeted drugs, other medications can modulate the immune response, aiding in the clearance of HPV infection and cervical lesion. The Nr‐CWS is an effective and safe drugs for treatment of HSIL in Chinese women,^330^ and recent studies found it may through stimulating FPR3 to enhance DCs‐mediated differentiation, activate the immune response of cervical tissue.^331^ Additionally, other pertinent studies demonstrate that it can upregulate T cells and inhibit PD/PD‐1 pathway, ameliorating the local immune status in HPV infection and CIN patients.^332^ Imiquimod can bind to Toll‐like receptors 7 and 8 of macrophages, producing IFNs, activate the CD8+ T cells. A completed phase II clinical trial has shown that topical treatment with imiquimod is effective of the regression of HSIL^333^ (NCT03233412). And the clinical trials have explored the combination of imiquimod and HPV therapeutic vaccines (NCT01957878 and NCT00788164).

#### 3.1.3 Adoptive T cell therapy

ATC involves the ex vivo expansion and manipulation of autologous immune cells to enhance their antitumor activity, followed by their reinfusion into the patient, so that the T cells will target tumor antigens to promote tumor regression.^334^ ACT have three major categories, tumor‐infiltrating lymphocytes (TILs),^335^ engineered T‐cell receptor T cells (TCR‐T),^336^ and chimeric antigen receptor (CAR) T cells.^337^ CAR‐T have been approved by US FDA for the therapy of acute lymphocytic leukemia. In the cervical cancer, NCT02280811 and NCT01585428 are the already completed studies about TCR‐T and TILs in HPV‐related cervical cancer, which showed potent validity.^338^ (Further pertinent studies are summarized in Table 5.) However, ACT for solid cancers is challenging because solid cancers are less sensitive to T cell‐mediated disruption in the tumor microenvironment. In addition, T cells are usually depleted when continuously exposed to their target antigen, despite the ongoing anticervical cancer effect. Induced pluripotent stem cell (iPSC) technology enhances the cytotoxicity of virus‐specific CTLs,^339^ which can survive long in vivo like young memory T cells, provide sustained tumor suppression, and constitute promising immunotherapy for cervical cancer.

**TABLE 5:** Clinical trials of adoptive T cell therapy for HPV‐related cancers.

Disease	Start date	Number	Intervention	Mechanism	Status	Phase	Sources
Metastatic HPV‐associated cancer (including cervical cancer)	2014.10	12	E6 TCR cells	TCR‐T	Completed	I/II	NCT02280811^338^
Human papillomavirus‐associated cancers	2017.1	180	E7 TCR cells	TCR‐T	Recruiting	I/II	NCT02858310
Cervical cancer head and neck squamous cell carcinoma	2018.9	20	HPV E6 specific TCR‐T cells	TCR‐T	Unknown	I	NCT03578406
Gastric, breast, cervical, lung and other KK‐LC‐1 positive epithelial cancers	2022.3	100	KK‐LC‐1 TCR	TCR‐T	Recruiting	I	NCT05035407
HPV‐16 positive advanced cervical, anal, or head and neck cancers	2022.7	12	CRTE7A2‐01 TCR‐T cell	TCR‐T	Recruiting	I	NCT05122221
Cervical cancer	2022.8	18	HPV16 E6 TCR T Cells (TC‐E202 cells)	TCR‐T	Recruiting	I/II	NCT05357027
Gastric, breast, cervical, and lung cancer	2022.9	42	KK‐LC‐1 TCR‐T cells	TCR‐T	Recruiting	I	NCT05483491
Human papillomavirus (HPV)‐associated cancers	2023.3	20	E7 TCR‐T cells	TCR‐T	Recruiting	II	NCT05686226
HPV‐18 positive advanced solid tumor	2023.3	17	HRYZ‐T101 TCR‐T Cell	TCR‐T	Not yet recruiting	I	NCT05787535
Advanced HPV‐associated cancers	2023.4	15	E7 TCR‐T cells	TCR‐T	Suspended	I/II	NCT05639972
Cervical cancer	2017.11	29	Cervical cancer‐specific CAR‐T cells	CAR‐T	Unknown	I/II	NCT03356795
Solid tumor (including cervical cancer)	2020.8	30	Autologous aPD‐L1 armored anti‐CD22 CAR T cells	CAR‐T	Recruiting	I	NCT04556669
CD70‐positive advanced/metastatic solid tumors (including cervical cancer)	2021.11	36	CD70 CAR‐T cells	CAR‐T	Recruiting	I	NCT05420545
CD70‐positive advanced/metastatic solid tumors (including cervical cancer)	2022.5	36	CD70 CAR‐T cells	CAR‐T	Recruiting	I	NCT05518253
CD70‐positive advanced/metastatic solid tumors (including cervical cancer)	2022.7	48	CD70 CAR‐T cells	CAR‐T	Recruiting	I	NCT05468190
Metastatic human papillomavirus‐associated cancers (including cervical cancer)	2012.4	29	Young TIL	TILs	Completed	II	NCT01585428^355^
Cervical carcinoma	2017.6	189	LN‐145 + pembrolizumab	TILs	Recruiting	I	NCT03108495
Cervical carcinoma	2019.10	10	CCRT + TIL	TILs	Active, not recruiting	I	NCT04443296
Advanced malignancies (including cervical cancer)	2021.11	50	Drug product De‐TIL‐0255	TILs	Active, not recruiting	I	NCT05107739
Gynecological malignancies	2022.4	30	Oripalimab + ScTIL	TILs	Not yet recruiting	II	NCT05342506
Advanced solid tumors (including cervical cancer)	2022.5	15	Autologous tumor infiltrating lymphocytes (TILs)	TILs	Recruiting	I	NCT05366478
Advanced cervical cancer	2022.7	20	Autologous tumor infiltrating lymphocytes (C‐TIL052A) injection	TILs	Recruiting	I	NCT05475847

*Data sources*: clinical registration website (https://clinicaltrials.gov), excluding the withdrawal and terminal trials, as of July 2023.

### 3.2 Medication

HPVE6/E7 is the primary pathogenic factor, and there is ongoing investigation into specific antibodies targeting E6/E7. However, the intracellular location of E6/E7 poses a challenge to conventional therapeutic antibodies, hindering their penetration into cells. Recent studies focusing on nanobodies targeting E6/E7 have shown promise in circumventing this limitation. Nonetheless, further research is needed since these studies remain in the preclinical stage. Consequently, there is currently no specific drug available for HPV infection. The ongoing in‐depth study of HPV pathogenesis has led to the identification of various pathogenic pathways as potential therapeutic targets for HPV‐related cervical lesions. These encompass inhibiting the cell cycle inhibition, suppressing cell proliferation, promoting apoptosis, as well as modulating cellular pathways and regulating the vaginal microenvironment.

In the context of targeted therapy for cervical cancer, in addition to targeted immune checkpoint inhibitors employed in immunotherapy, anti‐VEGF antibodies are widely employed in the treatment of cervical cancer. Currently, the standard first‐line therapy for recurrent or metastatic cervical cancer involves the combination of chemotherapy and bevacizumab.^356^, ^357^ Ongoing research is exploring the use of other antiangiogenic drugs and their combinations with different agents, including immune checkpoint inhibitors, in clinical trials (NCT02921269, NCT03074513, NCT03074513, and NCT04551950), with the aim of offering novel clinical options for patients with advanced cervical cancer.

Additional drugs have been utilized in the treatment of HPV infection and cervical precancerous lesions. Recombinant human IFN α−2b, an antiviral drug, has gained approval for HPV infection therapy. Its topical application aids in the clearance of HPV infection by enhancing mucosal immunity.^358^, ^359^ However, its efficacy in treating cervical lesions appears to be less satisfactory, leading to its usage primarily as an adjuvant therapy.^360^ REBACIN® is a recently discovered drug that has demonstrated a potent effect on clearing HPV infection. Enriched with the bioactive factor AVF, clinical trials and retrospective studies have confirmed its efficacy in treating HPV infection.^361^, ^362^ Recent research has also shown that REBACIN® can clear HPV infection by inhibiting the E6/E7 oncogenes.^363^ Considering the influence of vaginal microbiota on HPV infection and cervical lesions, modulating the VMB appears to be a practical approach for assisting in the clearance of HPV infection.^364^ A clinical trial (NCT03372395) found that sustained use of vaginal Lactobacillus rhamnosus BMX 54 can restore the vaginal ecosystem and benefit HPV clearance,^365^ However, the results seem inconsistent with another study (NCT01599416),^366^ highlighting the need for larger randomized studies to confirm these findings. Furthermore, a recent preclinical study demonstrated that the supernatants of lysates and heat‐inactivated Lacticaseibacillus casei LH23 can inhibit the expression of HPV E6/E7, thus illustrating the potential anticancer effects of probiotics.^367^ Traditional Chinese and herbal medicines, such as paiteling,^368^ myrtle,^369^ and curcumin,^370^ have also been investigated in clinical trials for the treatment of HPV infection. Additionally, in preclinical experiments, other Chinese traditional and herbal medicines as well as some natural phytochemicals, such as realgar,^371^ securidaca–saponins,^372^ ficus carica,^373^ juglone,^374^ can also play the same role. Certain drugs used for treatment of other disease, such as anti‐inflammatory drugs,^375^ metformin,^376^ artesunate,^377^ and antifungal agent ciclopirox,^378^ may also have broad applications in the clinical management of HPV infection.

### 3.3 Gene editing

As previously stated, the overexpression of E6 and E7 oncogenes closely correlates with the progression of cervical lesions. Consequently, directly knockout of these target genes can effectively inhibit lesion progression. Recent advancements in gene editing technology have realized this prospect. Zinc‐finger nucleases (ZFNs), transcription activator‐like endonucleases (TALENs), and CRISPR‐associated Cas9 endonucleases are three major generations of genome editing tools. Among them, CRISPR/Cas9 is more specific, efficient, and associated with fewer off‐target effects compared with the others.^379^ Studies have shown that knocking out the E6 and E7 oncogenes using the CRISPR/Cas system can inhibit tumor growth. Targeting the E6 oncogene leads to reactivation of the p53 tumor suppressor pathway,^380^ while targeting the E7 oncogene results in the restoration of Rb tumor suppressor pathway.^381^ Moreover, recent studies have demonstrated that CRISPR can directly hyperactivate p53 and eliminate HPV‐driven cervical cancer cells.^382^ A plethora of preclinical studies have shown the feasibility of gene editing technology for the treatment of HPV‐associated cervical lesions.^383^, ^384^, ^385^ Currently, there are only two phase I clinical trials investigating the use of TALEN and CRISPR/Cas9 in the treatment of cervical precancerous lesions (NCT03057912 and NCT02800369).

### 3.4 Other nonsurgical treatment

Radical surgery is the preferred treatment option for most patients with early‐stage cervical cancer and will not be further discussed here. In cases of precancerous conditions, the excisional technique, including large loop excision of the TZ or cold knife conization, is recommended for patients with CINII‐III.^386^ However, many women may hesitate to undergo these procedures due to concerns about potential preterm labor resulting from cervical incompetence and the risk of cervical adhesions leading to secondary amenorrhea.^387^ Therefore, this section will primarily focus on other nonsurgical alternatives for the treatment of cervical precancerous lesions.

Photodynamic therapy (PDT) is a modern and noninvasive treatment modality employed in the management of both oncological and nononcological conditions. It relies on the local or systemic administration of a photosensitive compound called a photosensitizer (PS). The photocytotoxic reactions induced by PDT occur exclusively within the pathological tissues in the vicinity of PS distribution, enabling selective destruction.^388^ In the case of HPV‐related cervical lesions, PDT finds wide application in the treatment of precancerous lesions and early‐stage cervical cancer.^389^ Researchers have also discovered that PDT can promote the regression of HPV infection in patients after conization or postmenopause.^390^ Since the effectiveness of PDT treatment depends on the PS, ongoing research and development of efficient PS can enhance the therapeutic outcomes of PDT, representing a significant area of investigation for future studies. In addition to PDT, other treatments such as cryotherapy, thermocoagulation,^391^, ^392^ and focused ultrasound^393^, ^394^ are commonly used for cervical precancerous lesions. The selection of specific treatment modality should be predicated upon the extent and scope of cervical lesions.

## 4 CONCLUSION

HPV infection imposes significant economic and psychological burdens on patients. The pathogenicity of HPV is closely linked to viral oncoproteins. Microscopic damage to the cervical mucosal epithelium facilitates HPV infection and the invasion of basal cells. Following invasion, the virus synthesizes oncoproteins, utilizes host components for replication, and releases infectious virus particles during basal cell differentiation, thereby completing its life cycle. Viruses can evade the immune response by affecting both innate and adaptive immunity. Failure to promptly clear HPV infection leads to persistent infection and the development of cervical lesions. The expression of viral oncoproteins in host cells disrupts the normal cell division cycle, inducing dysregulating several signaling pathways (such as Notch, Wnt, HIPPO, PI3K/AKT, and RAS pathways) and targeting host proteins involved in cell proliferation, ultimately resulting in uncontrolled cell proliferation. Moreover, HPV can inhibit cell apoptosis and enhance cell metastasis and invasiveness, which are crucial hallmarks of cancer cells. Integration of the HPV virus genome induces alterations in the host genome, representing a significant component of the carcinogenesis process. This integration can result from host chromosome instability due to excessive cell proliferation or the loss of cell proliferation regulation. Host and environmental factors, including the vaginal microenvironment and genetic factors, collectively contribute as significant elements in cervical lesion development. Numerous molecules and signaling pathways involved in the pathogenic mechanism of HPV have potential as therapeutic targets, and there remain several unexplored avenues for future basic research. Commonly used clinical drugs for HPV infection include recombinant IFN and REBACIN® among others. However, the clinical efficacy of these drugs is limited, underscoring the urgent need for research and development of more specific treatments. For cervical precancerous lesions, observation treatment may be appropriate for CINI, whereas CIN II and higher grades necessitate clinical intervention. Common clinical interventions include PDT, cryotherapy, thermal ablation, and ultrasound focusing, among others. The choice of treatment should be determined based on the extent and severity of the lesion. The primary treatment for cervical cancer consists of surgery combined with radiotherapy and chemotherapy. However, patients afflicted with positive lymph nodes, locally advanced disease, or metastatic cervical cancer still face a grim clinical prognosis. The development of therapeutic HPV vaccines holds promise for expanding treatment options for these patients. While HPV DNA vaccines have advanced to later stages of development, most other vaccine types are currently undergoing phase I/II clinical trials. Preliminary clinical trials have demonstrated the effectiveness and safety of the majority of vaccines, warranting large‐scale phase III clinical trials and resolution of technical challenges associated with vaccine mass production. Furthermore, immune checkpoint inhibitors represent a significant component of cervical cancer immunotherapy, with PD‐L1/PD‐1 checkpoint inhibitors being the most extensively studied. Currently, pembrolizumab is approved for advanced cervical cancer, and clinical trials for other similar drugs are also underway. The amalgamation of diverse medications, including their coadministration with vaccines, constitutes a burgeoning sphere of inquiry in present clinical research, demonstrating potential as an innovative treatment approach for cervical cancer in the future. ATC therapy, an emerging form of immunotherapy, is currently undergoing clinical trials related to cervical lesions. Encouraging results from its application in the treatment of other diseases have engendered optimism among researchers. Ongoing advancements in gene editing technology, aimed at enhancing its effectiveness, safety, and specificity, hold the potential to enable precise gene therapy for cervical cancer in the future.

## AUTHOR CONTRIBUTIONS

Jiatian Ye contributed with the manuscript drafting and drawing the graphics. Lan Zheng performed manuscript revision and editing. Yuedong He contributed with the manuscript revision and financial support. Xiaorong Qi performed the manuscript review and editing. All authors have agreed to the final submitted version.

## CONFLICT OF INTEREST STATEMENT

The authors declare that they have no competing interests.

## ETHICS STATEMENT

Not applicable.

## Data Availability

Not applicable.

## References

[1] Brüggmann D , Kayser L , Jaque J , Bundschuh M , Klingelhöfer D , Groneberg DA . Human papilloma virus: global research architecture assessed by density‐equalizing mapping. Oncotarget. 2018;9(31):21965‐21977. 29774116 10.18632/oncotarget.25136 PMC5955169

[2] Campos‐Romero A , Anderson KS , Longatto‐Filho A , et al. The burden of 14 hr‐HPV genotypes in women attending routine cervical cancer screening in 20 states of Mexico: a cross‐sectional study. Sci Rep. 2019;9(1):10094. 31300693 10.1038/s41598-019-46543-8 PMC6626130

[3] de Sanjosé S , Brotons M , Pavón MA . The natural history of human papillomavirus infection. Best Pract Res Clin Obstet Gynaecol. 2018;47:2‐13. 28964706 10.1016/j.bpobgyn.2017.08.015

[4] Faridi R , Zahra A , Khan K , Idrees M . Oncogenic potential of Human Papillomavirus (HPV) and its relation with cervical cancer. Virol J. 2011;8:269. 21635792 10.1186/1743-422X-8-269 PMC3118362

[5] Forman D , de Martel C , Lacey CJ , et al. Global burden of human papillomavirus and related diseases. Vaccine. 2012;30(5):F12‐F23. Suppl. 23199955 10.1016/j.vaccine.2012.07.055

[6] Vashisht S , Mishra H , Mishra PK , Ekielski A , TalegaonkarS . Structure, genome, infection cycle and clinical manifestations associated with human papillomavirus. Curr Pharm Biotechnol. 2019;20(15):1260‐1280. 31376818 10.2174/1389201020666190802115722

[7] Yu L , Majerciak V , Zheng ZM . HPV16 and HPV18 genome structure, expression, and post‐transcriptional regulation. Int J Mol Sci. 2022;23(9):4943. 35563334 10.3390/ijms23094943 PMC9105396

[8] Graham SV . The human papillomavirus replication cycle, and its links to cancer progression: a comprehensive review. Clin Sci Lond Engl 1979. 2017;131(17):2201‐2221. 10.1042/CS20160786 28798073

[9] Regauer S , Reich O . The origin of Human Papillomavirus (HPV)‐induced cervical squamous cancer. Curr Opin Virol. 2021;51:111‐118. 34655910 10.1016/j.coviro.2021.09.012

[10] Deng H , Hillpot E , Mondal S , Khurana KK , Woodworth CD . HPV16‐immortalized cells from human transformation zone and endocervix are more dysplastic than ectocervical cells in organotypic culture. Sci Rep. 2018;8(1):15402. 30337615 10.1038/s41598-018-33865-2 PMC6194146

[11] Loopik DL , Bentley HA , Eijgenraam MN , IntHout J , Bekkers RLM , Bentley JR . The natural history of cervical intraepithelial neoplasia grades 1, 2, and 3: a systematic review and meta‐analysis. J Low Genit Tract Dis. 2021;25(3):221‐231. 34176914 10.1097/LGT.0000000000000604

[12] Srisuttayasathien M , Manchana T . Adherence to follow‐up in women with cervical intraepithelial neoplasia grade 1. Taiwan J Obstet Gynecol. 2021;60(1):56‐59. 33495009 10.1016/j.tjog.2020.11.008

[13] Li TY , Wu ZN , Jiang MY , et al. Association between high risk human papillomavirus DNA load and cervical lesions in different infection status. Zhonghua Zhong Liu Za Zhi. 2018;40(6):475‐480. 29936777 10.3760/cma.j.issn.0253-3766.2018.06.015

[14] Kamal M . Cervical pre‐cancers: biopsy and immunohistochemistry. CytoJournal. 2022;19:38. 35928531 10.25259/CMAS_03_13_2021 PMC9345137

[15] Nunes RAL , Morale MG , Silva GÁF , Villa LL , Termini L . Innate immunity and HPV: friends or foes. Clin Sao Paulo. 2018;73(1):e549s. suppl. 10.6061/clinics/2018/e549s PMC6157093 30328949

[16] Sharifian K , Shoja Z , Jalilvand S . The interplay between human papillomavirus and vaginal microbiota in cervical cancer development. Virol J. 2023;20(1):73. 37076931 10.1186/s12985-023-02037-8 PMC10114331

[17] Okunade KS . Human papillomavirus and cervical cancer. J Obstet Gynaecol J Inst Obstet Gynaecol. 2020;40(5):602‐608. 10.1080/01443615.2019.1634030 PMC7062568 31500479

[18] Oyervides‐Muñoz MA , Pérez‐Maya AA , Rodríguez‐Gutiérrez HF , et al. Understanding the HPV integration and its progression to cervical cancer. Infect Genet Evol. 2018;61:134‐144. 29518579 10.1016/j.meegid.2018.03.003

[19] Kang SD , Chatterjee S , Alam S , et al. Effect of productive human papillomavirus 16 infection on global gene expression in cervical epithelium. J Virol. 2018;92(20). 10.1128/JVI.01261-18 PMC6158420 30045992

[20] St Laurent J , Luckett R , Feldman S . HPV vaccination and the effects on rates of HPV‐related cancers. Curr Probl Cancer. 2018;42(5):493‐506. 30041818 10.1016/j.currproblcancer.2018.06.004

[21] Arbyn M , Xu L , Simoens C , PP Martin‐Hirsch . Prophylactic vaccination against human papillomaviruses to prevent cervical cancer and its precursors. Cochrane Database Syst Rev. 2018;5(5):CD009069. 29740819 10.1002/14651858.CD009069.pub3 PMC6494566

[22] Eriksen DO , Jensen PT , Schroll JB , Hammer A . Human papillomavirus vaccination in women undergoing excisional treatment for cervical intraepithelial neoplasia and subsequent risk of recurrence: a systematic review and meta‐analysis. Acta Obstet Gynecol Scand. 2022;101(6):597‐607. 35470865 10.1111/aogs.14359 PMC9564558

[23] Poddar P , Maheshwari A . Surgery for cervical cancer: consensus & controversies. Indian J Med Res. 2021;154(2):284‐292. 34854431 10.4103/ijmr.IJMR_4240_20 PMC9131770

[24] Cohen PA , Jhingran A , Oaknin A , DennyL . Cervical cancer. Lancet Lond Engl. 2019;393(10167):169‐182. 10.1016/S0140-6736(18)32470-X 30638582

[25] Tewari KS , Sill MW , Long HJ , et al. Improved survival with bevacizumab in advanced cervical cancer. N Engl J Med. 2014;370(8):734‐743. 24552320 10.1056/NEJMoa1309748 PMC4010094

[26] Colombo N , Dubot C , Lorusso D , et al. Pembrolizumab for persistent, recurrent, or metastatic cervical cancer. N Engl J Med. 2021;385(20):1856‐1867. 34534429 10.1056/NEJMoa2112435

[27] Pentland I , Campos‐León K , Cotic M , et al. Disruption of CTCF‐YY1‐dependent looping of the human papillomavirus genome activates differentiation‐induced viral oncogene transcription. PLoS Biol. 2018;16(10):e2005752. 30359362 10.1371/journal.pbio.2005752 PMC6219814

[28] Raff AB , Woodham AW , Raff LM , et al. The evolving field of human papillomavirus receptor research: a review of binding and entry. J Virol. 2013;87(11):6062‐6072. 23536685 10.1128/JVI.00330-13 PMC3648114

[29] Mikuličić S , Fritzen A , Scheffer K , et al. Tetraspanin CD9 affects HPV16 infection by modulating ADAM17 activity and the ERK signalling pathway. Med Microbiol Immunol. 2020;209(4):461‐471. 32385608 10.1007/s00430-020-00671-5 PMC7206579

[30] Xie J , Heim EN , Crite M , DiMaio D . TBC1D5‐catalyzed cycling of Rab7 is required for retromer‐mediated human papillomavirus trafficking during virus entry. Cell Rep. 2020;31(10):107750. 32521275 10.1016/j.celrep.2020.107750 PMC7339955

[31] Guion L , Bienkowska‐Haba M , DiGiuseppe S , Florin L , Sapp M . PML nuclear body‐residing proteins sequentially associate with HPV genome after infectious nuclear delivery. PLoS Pathog. 2019;15(2):e1007590. 30802273 10.1371/journal.ppat.1007590 PMC6405170

[32] Taylor JR , Fernandez DJ , Thornton SM , et al. Heterotetrameric annexin A2/S100A10 (A2t) is essential for oncogenic human papillomavirus trafficking and capsid disassembly, and protects virions from lysosomal degradation. Sci Rep. 2018;8(1):11642. 30076379 10.1038/s41598-018-30051-2 PMC6076308

[33] Breiner B , Preuss L , Roos N , et al. Refolding and in vitro characterization of human papillomavirus 16 minor capsid protein L2. Biol Chem. 2019;400(4):513‐522. 30375341 10.1515/hsz-2018-0311

[34] Broniarczyk J , Massimi P , Pim D , et al. Phosphorylation of human papillomavirus type 16 L2 contributes to efficient virus infectious entry. J Virol. 2019;93(13). 10.1128/JVI.00128-19 PMC6580975 30996086

[35] Day PM , Weisberg AS , Thompson CD , et al. Human papillomavirus 16 capsids mediate nuclear entry during infection. J Virol. 2019;93(15). 10.1128/JVI.00454-19 PMC6639283 31092566

[36] Lai KY , Rizzato M , Aydin I , Villalonga‐Planells R , Drexler HCA , Schelhaas M . A Ran‐binding protein facilitates nuclear import of human papillomavirus type 16. PLoS Pathog. 2021;17(5):e1009580. 33974675 10.1371/journal.ppat.1009580 PMC8139508

[37] Pim D , Broniarczyk J , Siddiqa A , Massimi P , Banks L . Human papillomavirus 16 L2 recruits both retromer and retriever complexes during retrograde trafficking of the viral genome to the cell nucleus. J Virol. 2021;95(3). 10.1128/JVI.02068-20 PMC7925090 33177206

[38] Zhang P , Monteiro da Silva G , Deatherage C , Burd C , DiMaio D . Cell‐penetrating peptide mediates intracellular membrane passage of human papillomavirus l2 protein to trigger retrograde trafficking. Cell. 2018;174(6):1465‐1476.e13. 30122350 10.1016/j.cell.2018.07.031 PMC6128760

[39] Harwood MC , Dupzyk AJ , Inoue T , DiMaio D , Tsai B . p120 catenin recruits HPV to γ‐secretase to promote virus infection. PLoS Pathog. 2020;16(10):e1008946. 33085724 10.1371/journal.ppat.1008946 PMC7577436

[40] Xie J , Zhang P , Crite M , Lindsay CV , DiMaio D . Retromer stabilizes transient membrane insertion of L2 capsid protein during retrograde entry of human papillomavirus. Sci Adv. 2021;7(27). 10.1126/sciadv.abh4276 PMC11057781 34193420

[41] Ishii Y , Yamaji T , Sekizuka T , et al. Folliculin prevents lysosomal degradation of human papillomavirus to support infectious cell entry. J Virol. 2023;97(5):e0005623. 37167561 10.1128/jvi.00056-23 PMC10231244

[42] Crite M , DiMaio D . Human papillomavirus L2 capsid protein stabilizes γ‐secretase during viral infection. Viruses. 2022;14(4). 10.3390/v14040804 PMC9027364 35458534

[43] Schweiger L , Lelieveld‐Fast LA , Mikuličić S , et al. HPV16 induces formation of Virus‐p62‐PML hybrid bodies to enable infection. Viruses. 2022;14(7). 10.3390/v14071478 PMC9315800 35891458

[44] Rizzato M , Mao F , Chardon F , et al. Master mitotic kinases regulate viral genome delivery during papillomavirus cell entry. Nat Commun. 2023;14(1):355. 36683055 10.1038/s41467-023-35874-w PMC9868124

[45] Zwolinska K , Bienkowska‐Haba M , Scott RS , Keiffer T , Sapp M . Experimental support for human papillomavirus genome amplification early after infectious delivery. J Virol. 2023;97(6):e0021423. 37223953 10.1128/jvi.00214-23 PMC10308938

[46] Orav M , Gagnon D , Archambault J . Interaction of the human papillomavirus E1 helicase with UAF1‐USP1 promotes unidirectional theta replication of viral genomes. mBio. 2019;10(2). 10.1128/mBio.00152-19 PMC6426595 30890612

[47] Laaneväli A , Ustav M , Ustav E , Piirsoo M . E2 protein is the major determinant of specificity at the human papillomavirus origin of replication. PLoS One. 2019;14(10):e0224334. 31644607 10.1371/journal.pone.0224334 PMC6808437

[48] Yilmaz G , Biswas‐Fiss EE , Biswas SB . Sequence‐dependent interaction of the human papillomavirus E2 protein with the DNA elements on its DNA replication origin. Int J Mol Sci. 2023;24(7):6555. 37047526 10.3390/ijms24076555 PMC10095481

[49] Chojnacki M , Melendy T . The HPV E2 transcriptional transactivation protein stimulates cellular DNA polymerase epsilon. Viruses. 2018;10(6). 10.3390/v10060321 PMC6024689 29895728

[50] Wang S , Gramm V , Laport E , Holland‐Letz T , Alonso A , Schenkel J . Transgenic HPV11‐E2 protein modulates URR activity in vivo. Transgenic Res. 2023;32(1‐2):67‐76. 36826606 10.1007/s11248-023-00336-y PMC10102070

[51] Piirsoo A , Kala M , Sankovski E , Ustav M , Piirsoo M . Uncovering the role of the E1 protein in different stages of human papillomavirus 18 genome replication. J Virol. 2020;94(20). 10.1128/JVI.00674-20 PMC7527042 32759324

[52] Lototskaja E , Sahharov O , Piirsoo M , Kala M , Ustav M , Piirsoo A . Cyclic AMP‐dependent protein kinase exhibits antagonistic effects on the replication efficiency of different human papillomavirus types. J Virol. 2021;95(13):e0025121. 33853963 10.1128/JVI.00251-21 PMC8316020

[53] Jose L , DeSmet M , Androphy EJ . Pyk2 regulates human papillomavirus replication by tyrosine phosphorylation of the E2 protein. J Virol. 2020;94(20). 10.1128/JVI.01110-20 PMC7527049 32727877

[54] Jose L , Androphy EJ , DeSmet M . Phosphorylation of the human papillomavirus E2 protein at tyrosine 138 regulates episomal replication. J Virol. 2020;94(14). 10.1128/JVI.00488-20 PMC7343196 32350070

[55] Piirsoo A , Piirsoo M , Kala M , et al. Activity of CK2α protein kinase is required for efficient replication of some HPV types. PLoS Pathog. 2019;15(5):e1007788. 31091289 10.1371/journal.ppat.1007788 PMC6538197

[56] DeSmet M , Jose L , Isaq N , Androphy EJ . Phosphorylation of a conserved tyrosine in the papillomavirus E2 protein regulates Brd4 binding and viral replication. J Virol. 2019;93(10). 10.1128/JVI.01801-18 PMC6498050 30842331

[57] Prabhakar AT , James CD , Das D , et al. CK2 phosphorylation of human papillomavirus 16 E2 on serine 23 promotes interaction with TopBP1 and is critical for e2 interaction with mitotic chromatin and the viral life cycle. mBio. 2021;12(5):e0116321. 34544280 10.1128/mBio.01163-21 PMC8546539

[58] Prabhakar AT , James CD , Fontan CT , et al. Human papillomavirus 16 E2 interaction with TopBP1 is required for E2 and viral genome stability during the viral life cycle. J Virol. 2023;97(3):e00063‐23. Banks L, ed. 36840558 10.1128/jvi.00063-23 PMC10062148

[59] Prabhakar AT , James CD , Das D , et al. Interaction with TopBP1 is required for human papillomavirus 16 E2 plasmid segregation/retention function during mitosis. J Virol. 2022;96(16):e0083022. 35880889 10.1128/jvi.00830-22 PMC9400484

[60] Ferguson J , Campos‐León K , Pentland I , et al. The chromatin insulator CTCF regulates HPV18 transcript splicing and differentiation‐dependent late gene expression. PLoS Pathog. 2021;17(11):e1010032. 34735550 10.1371/journal.ppat.1010032 PMC8594839

[61] Dreer M , Blondzik S , Straub E , Iftner T , Stubenrauch F . Contribution of HDAC3 to transcriptional repression by the human papillomavirus 31 E8^E2 protein. J Gen Virol. 2020;101(7):751‐759. 32421493 10.1099/jgv.0.001438

[62] Murakami I , Iwata T , Morisada T , Tanaka K , Aoki D . Nucleosome positioning on episomal human papillomavirus DNA in cultured cells. Pathogens. 2021;10(6). 10.3390/pathogens10060772 PMC8235217 34205361

[63] Das D , Bristol ML , Smith NW , et al. Werner helicase control of human papillomavirus 16 E1‐E2 DNA replication is regulated by SIRT1 deacetylation. mBio. 2019;10(2). 10.1128/mBio.00263-19 PMC6426601 30890607

[64] Liblekas L , Piirsoo A , Laanemets A , et al. Analysis of the replication mechanisms of the human papillomavirus genomes. Front Microbiol. 2021;12:738125. 34733254 10.3389/fmicb.2021.738125 PMC8558456

[65] Spriggs CC , Blanco LZ , Maniar KP , Laimins LA . Expression of HPV‐induced DNA damage repair factors correlates with CIN progression. Int J Gynecol Pathol. 2019;38(1):1‐10. 29995652 10.1097/PGP.0000000000000477 PMC6295252

[66] Khurana S , Markowitz TE , Kabat J , McBride AA . Spatial and functional organization of human papillomavirus replication foci in the productive stage of infection. mBio. 2021;12(6):e0268421. 34749533 10.1128/mBio.02684-21 PMC8576538

[67] Kaminski P , Hong S , Kono T , Hoover P , Laimins L . Topoisomerase 2β induces DNA breaks to regulate human papillomavirus replication. mBio. 2021;12(1). 10.1128/mBio.00005-21 PMC7885102 33563836

[68] Thomas Y , Androphy EJ . Acetylation of E2 by P300 mediates topoisomerase entry at the papillomavirus replicon. J Virol. 2019;93(7). 10.1128/JVI.02224-18 PMC6430547 30651357

[69] Hong S , Xu J , Li Y , et al. Topoisomerase IIβ‐binding protein 1 activates expression of E2F1 and p73 in HPV‐positive cells for genome amplification upon epithelial differentiation. Oncogene. 2019;38(17):3274‐3287. 30631149 10.1038/s41388-018-0633-1 PMC6486426

[70] Yu H , Wu C , Nilsson K , Kajitani N , Schwartz S . Adenosine causes read‐through into the late region of the HPV16 genome in a guanosine‐dependent manner. Virology. 2018;521:1‐19. 29864673 10.1016/j.virol.2018.05.019

[71] Xu X , Yuan S , Zhang X , Lou H . Immune response of plasmacytoid dendritic cells stimulated by human papillomavirus (HPV) E6 in an in vitro system. Med Sci Monit. 2020;26:e919770. 32089541 10.12659/MSM.919770 PMC7057736

[72] Guleria C , Suri V , Kapoor R , Minz RW , Aggarwal R . Human papillomavirus 16 infection alters the Toll‐like receptors and downstream signaling cascade: a plausible early event in cervical squamous cell carcinoma development. Gynecol Oncol. 2019;155(1):151‐160. 31375269 10.1016/j.ygyno.2019.07.023

[73] Uhlorn BL , Jackson R , Li S , Bratton SM , Van Doorslaer K , Campos SK . Vesicular trafficking permits evasion of cGAS/STING surveillance during initial human papillomavirus infection. PLoS Pathog. 2020;16(11):e1009028. 33253291 10.1371/journal.ppat.1009028 PMC7728285

[74] Chiang C , Pauli EK , Biryukov J , et al. The human papillomavirus E6 oncoprotein targets USP15 and TRIM25 to suppress RIG‐I‐mediated innate immune signaling. J Virol. 2018;92(6):e01737‐17. Jung JU, ed. 29263274 10.1128/JVI.01737-17 PMC5827370

[75] Miyauchi S , Kim SS , Jones RN , et al. Human papillomavirus E5 suppresses immunity via inhibition of the immunoproteasome and STING pathway. Cell Rep. 2023;42(5):112508. 37171962 10.1016/j.celrep.2023.112508 PMC10789500

[76] Ying Z , Li X , Dang H , Yin N , Gao C . Molecular immune mechanisms of HPV‐infected HaCaT cells in vitro based on toll‐like receptors signaling pathway. J Clin Lab Anal. 2020;34(3):e23101. 31785031 10.1002/jcla.23101 PMC7083446

[77] Rattay S , Hufbauer M , Hagen C , et al. Human beta papillomavirus type 8 E1 and E2 proteins suppress the activation of the RIG‐I‐Like receptor MDA5. Viruses. 2022;14(7). 10.3390/v14071361 PMC9317666 35891343

[78] Gusho E , Laimins LA . Human papillomaviruses sensitize cells to DNA damage induced apoptosis by targeting the innate immune sensor cGAS. PLoS Pathog. 2022;18(7):e1010725. 35877778 10.1371/journal.ppat.1010725 PMC9352202

[79] Luo X , Donnelly CR , Gong W , et al. HPV16 drives cancer immune escape via NLRX1‐mediated degradation of STING. J Clin Invest. 2020;130(4):1635‐1652. 31874109 10.1172/JCI129497 PMC7108911

[80] Castro‐Muñoz LJ , Manzo‐Merino J , Muñoz‐Bello JO , et al. The Human Papillomavirus (HPV) E1 protein regulates the expression of cellular genes involved in immune response. Sci Rep. 2019;9(1):13620. 31541186 10.1038/s41598-019-49886-4 PMC6754496

[81] James CD , Fontan CT , Otoa R , et al. Human papillomavirus 16 E6 and E7 synergistically repress innate immune gene transcription. mSphere. 2020;5(1). 10.1128/mSphere.00828-19 PMC6952203 31915229

[82] Rice S , Kim SM , Rodriguez C , et al. Suppression of a subset of interferon‐induced genes by human papillomavirus type 16 E7 via a cyclin dependent kinase 8‐dependent mechanism. Viruses. 2020;12(3). 10.3390/v12030311 PMC7150855 32183180

[83] Lo Cigno I , Calati F , Borgogna C , et al. Human papillomavirus E7 oncoprotein subverts host innate immunity via SUV39H1‐mediated epigenetic silencing of immune sensor genes. J Virol. 2020;94(4). 10.1128/JVI.01812-19 PMC6997746 31776268

[84] Poirson J , Suarez IP , Straub ML , et al. High‐risk mucosal human papillomavirus 16 (HPV16) E6 protein and cutaneous HPV5 and HPV8 E6 proteins employ distinct strategies to interfere with interferon regulatory factor 3‐mediated beta interferon expression. J Virol. 2022;96(10):e0187521. 35475668 10.1128/jvi.01875-21 PMC9131866

[85] Raikhy G , Woodby BL , Scott ML , et al. Suppression of stromal interferon signaling by human papillomavirus 16. J Virol. 2019;93(19). 10.1128/JVI.00458-19 PMC6744227 31292244

[86] Scott ML , Woodby BL , Ulicny J , et al. Human papillomavirus 16 E5 inhibits interferon signaling and supports episomal viral maintenance. J Virol. 2020;94(2). 10.1128/JVI.01582-19 PMC6955282 31666385

[87] Huang J , Diao G , Zhang Q , Chen Y , Han J , Guo J . E6‑regulated overproduction of prostaglandin E2 may inhibit migration of dendritic cells in human papillomavirus 16‑positive cervical lesions. Int J Oncol. 2020;56(4):921‐931. 32319556 10.3892/ijo.2020.4983 PMC7050979

[88] Manzo‐Merino J , Lagunas‐Martínez A , Contreras‐Ochoa CO , et al. The human papillomavirus (HPV) E6 oncoprotein regulates CD40 expression via the AT‐hook transcription factor AKNA. Cancers Basel. 2018;10(12). 10.3390/cancers10120521 PMC6316281 30562965

[89] Zhang J , Jin S , Li X , et al. Human papillomavirus type 16 disables the increased natural killer cells in early lesions of the cervix. J Immunol Res. 2019;2019:9182979. 31183395 10.1155/2019/9182979 PMC6512046

[90] Nie Y , Liu D , Yang W , et al. Increased expression of TIGIT and KLRG1 correlates with impaired CD56(bright) NK cell immunity in HPV16‐related cervical intraepithelial neoplasia. Virol J. 2022;19(1):68. 35413989 10.1186/s12985-022-01776-4 PMC9003970

[91] Zheng H , Zou Z , Wu X , et al. HPV11E7 inhibits IMQ‐induced chemokine and colony‐stimulating factor production in keratinocytes. Gene. 2020;760:145003. 32739587 10.1016/j.gene.2020.145003

[92] Ainouze M , Rochefort P , Parroche P , et al. Human papillomavirus type 16 antagonizes IRF6 regulation of IL‐1β. PLoS Pathog. 2018;14(8):e1007158. 30089163 10.1371/journal.ppat.1007158 PMC6124776

[93] Nw M , Ga A , Aa EF , Am H . Galectin‐3 and interleukin‐17: a potential role in the pathogenesis of human papilloma virus infection. J Cosmet Dermatol. 2022;21(6):2618‐2622. 34449950 10.1111/jocd.14430

[94] Zhu R , Wang W , Yang A , et al. Interactions between vaginal local cytokine IL‐2 and high‐risk human papillomavirus infection with cervical intraepithelial neoplasia in a Chinese population‐based study. Front Cell Infect Microbiol. 2023;13:1109741. 37256111 10.3389/fcimb.2023.1109741 PMC10225571

[95] Artaza‐Irigaray C , Molina‐Pineda A , Aguilar‐Lemarroy A , et al. E6/E7 and E6(*) from HPV16 and HPV18 upregulate IL‐6 expression independently of p53 in keratinocytes. Front Immunol. 2019;10:1676. 31396215 10.3389/fimmu.2019.01676 PMC6664019

[96] Lebeau A , Bruyere D , Roncarati P , et al. HPV infection alters vaginal microbiome through down‐regulating host mucosal innate peptides used by Lactobacilli as amino acid sources. Nat Commun. 2022;13(1):1076. 35228537 10.1038/s41467-022-28724-8 PMC8885657

[97] Aggarwal R , Sharma M , Mangat N , et al. Understanding HLA‐G driven journey from HPV infection to cancer cervix: adding missing pieces to the jigsaw puzzle. J Reprod Immunol. 2020;142:103205. 33099242 10.1016/j.jri.2020.103205

[98] Burassakarn A , Phusingha P , Yugawa T , et al. Human papillomavirus 16 E6 suppresses transporter associated with antigen‐processing complex in human tongue keratinocyte cells by activating lymphotoxin pathway. Cancers Basel. 2022;14(8). 10.3390/cancers14081944 PMC9028769 35454851

[99] Bashaw AA , Teoh SM , Tuong ZK , Leggatt GR , Frazer IH , Chandra J . HPV16 E7‐driven epithelial hyperplasia promotes impaired antigen presentation and regulatory T‐Cell development. J Invest Dermatol. 2019;139(12):2467‐2476. .e3. 31207230 10.1016/j.jid.2019.03.1162

[100] Bashaw AA , Zhou C , Yu M , et al. Regulatory T cells but not IL‐10 impair cell‐mediated immunity in human papillomavirus e7+ hyperplastic epithelium. J Invest Dermatol. 2021;141(5):1264‐1273. .e3. 33129828 10.1016/j.jid.2020.10.011

[101] Vattai A , Kremer N , Meister S , et al. Role of FoxP3‐positive regulatory T‐cells in regressive and progressive cervical dysplasia. J Cancer Res Clin Oncol. 2022;148(2):377‐386. 34739585 10.1007/s00432-021-03838-6 PMC11800877

[102] Cai H , Yan L , Liu N , Xu M , Cai H . IFI16 promotes cervical cancer progression by upregulating PD‐L1 in immunomicroenvironment through STING‐TBK1‐NF‐kB pathway. Biomed Pharmacother. 2020;123:109790. 31896065 10.1016/j.biopha.2019.109790

[103] Ling J , Sun Q , Tian Q , Shi H , Yang H , Ren J . Human papillomavirus 16 E6/E7 contributes to immune escape and progression of cervical cancer by regulating miR‐142‐5p/PD‐L1 axis. Arch Biochem Biophys. 2022;731:109449. 36288761 10.1016/j.abb.2022.109449

[104] Yu R , Wei Y , He C , et al. Integrative analyses of m6A regulators identify that METTL3 is associated with HPV status and immunosuppressive microenvironment in HPV‐related cancers. Int J Biol Sci. 2022;18(9):3874‐3887. 35813476 10.7150/ijbs.70674 PMC9254478

[105] Yigitliler A , Renner J , Simon C , Schneider M , Stubenrauch F , Iftner T . BRD4S interacts with viral E2 protein to limit human papillomavirus late transcription. J Virol. 2021;95(11). 10.1128/JVI.02032-20 PMC8139696 33731454

[106] Fu Y , Cao R , Schäfer M , et al. Expression of different L1 isoforms of Mastomys natalensis papillomavirus as mechanism to circumvent adaptive immunity. eLife. 2020;9:e57626. 32746966 10.7554/eLife.57626 PMC7402679

[107] Song Y , Wu X , Xu Y , et al. HPV E7 inhibits cell pyroptosis by promoting TRIM21‐mediated degradation and ubiquitination of the IFI16 inflammasome. Int J Biol Sci. 2020;16(15):2924‐2937. 33061806 10.7150/ijbs.50074 PMC7545706

[108] Metaxas G , Tsiambas E , Kavantzas N , Stavraka C , Lazaris EPAC , Thomopoulou GE . Impact of cyclin D1 deregulation in HPV‐mediated squamous intraepithelial lesions based on cell spots analysis. J Buon. 2020;25(2):792‐796. 32521869

[109] Li S , Yim MK , Yip KL , et al. E6‐encoded by cancer‐causing human papillomavirus interacts with aurora kinase a to promote HPV‐mediated carcinogenesis. J Virol. 2023;97(2):e0187222. 36715516 10.1128/jvi.01872-22 PMC9972942

[110] Kiran S , Dar A , Singh SK , Lee KY , Dutta A . The deubiquitinase USP46 is essential for proliferation and tumor growth of HPV‐transformed cancers. Mol Cell. 2018;72(5):823‐835. .e5. 30415951 10.1016/j.molcel.2018.09.019 PMC6294304

[111] Li M , Yang J , Liu K , et al. p16 promotes proliferation in cervical carcinoma cells through CDK6‐HuR‐IL1A axis. J Cancer. 2020;11(6):1457‐1467. 32047552 10.7150/jca.35479 PMC6995400

[112] Gandhi S , Nor Rashid N , Mohamad Razif MF , Othman S . Proteasomal degradation of p130 facilitate cell cycle deregulation and impairment of cellular differentiation in high‐risk Human Papillomavirus 16 and 18 E7 transfected cells. Mol Biol Rep. 2021;48(6):5121‐5133. 34169395 10.1007/s11033-021-06509-4

[113] James CD , Saini S , Sesay F , et al. Restoring the DREAM complex inhibits the proliferation of high‐risk HPV positive human cells. Cancers Basel. 2021;13(3). 10.3390/cancers13030489 PMC7866234 33513914

[114] Qiao L , Zhang Q , Zhang W , Chen JJ . The lysine acetyltransferase GCN5 contributes to human papillomavirus oncoprotein E7‐induced cell proliferation via up‐regulating E2F1. J Cell Mol Med. 2018;22(11):5333‐5345. 30079588 10.1111/jcmm.13806 PMC6201343

[115] Zhou Y , Pei F , Ji M , et al. WDHD1 facilitates G1 checkpoint abrogation in HPV E7 expressing cells by modulating GCN5. BMC Cancer. 2020;20(1):840. 32883234 10.1186/s12885-020-07287-1 PMC7469104

[116] Tian S , Zhang L , Li Y , et al. Human papillomavirus E7 oncoprotein promotes proliferation and migration through the transcription factor E2F1 in cervical cancer cells. Anticancer Agents Med Chem. 2021;21(13):1689‐1696. 33155930 10.2174/1871520620666201106085227

[117] Masuda Y , Saeki Y , Arai N , et al. Stepwise multipolyubiquitination of p53 by the E6AP‐E6 ubiquitin ligase complex. J Biol Chem. 2019;294(41):14860‐14875. 31492752 10.1074/jbc.RA119.008374 PMC6791319

[118] Conrady MC , Suarez I , Gogl G , et al. Structure of high‐risk papillomavirus 31 E6 oncogenic protein and characterization of E6/E6AP/p53 complex formation. J Virol. 2020;95(2). 10.1128/JVI.00730-20 PMC7944444 33115863

[119] Yi SA , Lee DH , Kim GW , et al. HPV‐mediated nuclear export of HP1γ drives cervical tumorigenesis by downregulation of p53. Cell Death Differ. 2020;27(9):2537‐2551. 32203172 10.1038/s41418-020-0520-5 PMC7429875

[120] Romero‐Medina MC , Venuti A , Melita G , et al. Human papillomavirus type 38 alters wild‐type p53 activity to pro type="Periodical"mote cell proliferation via the downregulation of integrin alpha 1 expression. PLoS Pathog. 2020;16(8):e1008792. 32813746 10.1371/journal.ppat.1008792 PMC7458291

[121] Jaiswal N , Nandi D , Cheema PS , Nag A . The anaphase‐promoting complex/cyclosome co‐activator, Cdh1, is a novel target of human papillomavirus 16 E7 oncoprotein in cervical oncogenesis. Carcinogenesis. 2022;43(10):988‐1001. 35738876 10.1093/carcin/bgac057

[122] Rangarajan A , Talora C , Okuyama R , et al. Notch signaling is a direct determinant of keratinocyte growth arrest and entry into differentiation. Embo J. 2001;20(13):3427‐3436. 11432830 10.1093/emboj/20.13.3427 PMC125257

[123] Khelil M , Griffin H , Bleeker MCG , et al. Delta‐like ligand‐notch1 signaling is selectively modulated by HPV16 E6 to promote squamous cell proliferation and correlates with cervical cancer prognosis. Cancer Res. 2021;81(7):1909‐1921. 33500246 10.1158/0008-5472.CAN-20-1996

[124] Lim J , Straub E , Stubenrauch F , Iftner T , Schindler M , Simon C . An enhanced triple fluorescence flow‐cytometry‐based assay shows differential activation of the Notch signaling pathway by human papillomavirus E6 proteins. Sci Rep. 2022;12(1):3000. 35194094 10.1038/s41598-022-06922-0 PMC8863805

[125] Yang Z , Liu H , Song R , et al. Reduced MAGI3 level by HPV18E6 contributes to Wnt/β‐catenin signaling activation and cervical cancer progression. FEBS Open Bio. 2021;11(11):3051‐3062. 10.1002/2211-5463.13298 PMC8564337 34510826

[126] Drews CM , Case S , Vande Pol SB . E6 proteins from high‐risk HPV, low‐risk HPV, and animal papillomaviruses activate the Wnt/β‐catenin pathway through E6AP‐dependent degradation of NHERF1. PLoS Pathog. 2019;15(4):e1007575. 31002735 10.1371/journal.ppat.1007575 PMC6493770

[127] Muñoz‐Bello JO , Olmedo‐Nieva L , Castro‐Muñoz LJ , et al. HPV‐18 E6 oncoprotein and its spliced isoform E6*I regulate the Wnt/β‐catenin cell signaling pathway through the TCF‐4 transcriptional factor. Int J Mol Sci. 2018;19(10). 10.3390/ijms19103153 PMC6214013 30322153

[128] Zhao L , Wang L , Zhang C , et al. E6‐induced selective translation of WNT4 and JIP2 promotes the progression of cervical cancer via a noncanonical WNT signaling pathway. Signal Transduct Target Ther. 2019;4:32. 31637011 10.1038/s41392-019-0060-y PMC6799841

[129] Yun HY , Kim MW , Lee HS , et al. Structural basis for recognition of the tumor suppressor protein PTPN14 by the oncoprotein E7 of human papillomavirus. PLoS Biol. 2019;17(7):e3000367. 31323018 10.1371/journal.pbio.3000367 PMC6668832

[130] Nishio M , To Y , Maehama T , et al. Endogenous YAP1 activation drives immediate onset of cervical carcinoma in situ in mice. Cancer Sci. 2020;111(10):3576‐3587. 32716083 10.1111/cas.14581 PMC7541006

[131] Hatterschide J , Castagnino P , Kim HW , et al. YAP1 activation by human papillomavirus E7 promotes basal cell identity in squamous epithelia. eLife. 2022:11. 10.7554/eLife.75466 PMC8959598 35170430

[132] Huang C , Lv X , Chen P , et al. Human papillomavirus targets the YAP1‐LATS2 feedback loop to drive cervical cancer development. Oncogene. 2022;41(30):3761‐3777. 35761037 10.1038/s41388-022-02390-y PMC10399300

[133] Messa L , Celegato M , Bertagnin C , et al. The dimeric form of HPV16 E6 is crucial to drive YAP/TAZ upregulation through the targeting of hScrib. Cancers Basel. 2021;13(16). 10.3390/cancers13164083 PMC8393709 34439242

[134] Chiang AJ , Li CJ , Tsui KH , et al. UBE2C drives human cervical cancer progression and is positively modulated by mTOR. Biomolecules. 2020;11(1). 10.3390/biom11010037 PMC7823929 33396624

[135] Wan PK , Leung TH , Siu MK , et al. HPV‐induced Nurr1 promotes cancer aggressiveness, self‐renewal, and radioresistance via ERK and AKT signaling in cervical cancer. Cancer Lett. 2021;497:14‐27. 33010383 10.1016/j.canlet.2020.09.025

[136] Wang M , Qiao X , Cooper T , et al. HPV E7‐mediated NCAPH ectopic expression regulates the carcinogenesis of cervical carcinoma via PI3K/AKT/SGK pathway. Cell Death Dis. 2020;11(12):1049. 33311486 10.1038/s41419-020-03244-9 PMC7732835

[137] Hou X , Qiao L , Liu R , Han X , Zhang W . Phosphorylation of RCC1 on serine 11 facilitates G1/S transition in HPV E7‐expressing cells. Biomolecules. 2021;11(7). 10.3390/biom11070995 PMC8301946 34356619

[138] Lin Z , Zhao Y , Li Q , et al. Sustained expression of HPV16 E7 oncoprotein promotes p‐AKT(Ser473)/p‐Src(Tyr527) signaling to drive precancerous lesions to invasive cervical cancer. Carcinogenesis. 2022;43(5):479‐493. 35134836 10.1093/carcin/bgac010

[139] Taute S , Böhnke P , Sprissler J , et al. The protein tyrosine phosphatase H1 PTPH1 supports proliferation of keratinocytes and is a target of the human papillomavirus type 8 E6 oncogene. Cells. 2019;8(3). 10.3390/cells8030244 PMC6468676 30875834

[140] Aggarwal D , Wadhwa N , Arora T , et al. Human telomerase RNA component (hTERC) gene expression and chromosome 7 ploidy correlate positively with histological grade of cervical intraepithelial neoplasia. Cytopathology. 2021;32(5):631‐639. 33848025 10.1111/cyt.12978

[141] Vliet‐Gregg PA , Robinson KL , Levan J , Matsumoto LR , Katzenellenbogen RA . NFX1‐123 is highly expressed in cervical cancer and increases growth and telomerase activity in HPV 16E6 expressing cells. Cancer Lett. 2019;449:106‐113. 30776478 10.1016/j.canlet.2019.02.024 PMC6433130

[142] Quist KM , Solorzano I , Wendel SO , et al. Cervical cancer development: implications of HPV16 E6E7‐NFX1‐123 regulated genes. Cancers Basel. 2021;13(24). 10.3390/cancers13246182 PMC8699269 34944802

[143] Tiwari D , Ray Das C , Sultana R , et al. Impact of modulation of telomerase and cancer stem‐cell marker OCT4 axis in cervical cancer pathogenesis with underlying HPV16 infection. J Cell Biochem. 2020;121(4):2782‐2791. 31692038 10.1002/jcb.29501

[144] Boon SS , Lee YC , Yip KL , et al. Interaction between human papillomavirus‐encoded E6 protein and AurB induces cell immortalization and proliferation—a potential target of intervention. Cancers Basel. 2023;15(9). 10.3390/cancers15092465 PMC10177266 37173932

[145] Liu Z , Teng L , Gao L , Wang H , Su Y , Li J . The role of eukaryotic translation initiation factor 5A‐1 (eIF5A‐1) gene in HPV 16 E6 induces cell growth in human cervical squamous carcinoma cells. Biochem Biophys Res Commun. 2018;504(1):6‐12. 30170728 10.1016/j.bbrc.2018.08.018

[146] Morales‐Garcia V , Contreras‐Paredes A , Martinez‐Abundis E , et al. The high‐risk HPV E6 proteins modify the activity of the eIF4E protein via the MEK/ERK and AKT/PKB pathways. FEBS Open Bio. 2020;10(12):2541‐2552. 10.1002/2211-5463.12987 PMC7714072 32981220

[147] Lee SA , Ho C , Troxler M , Lin CY , Chung SH . Non‐metabolic functions of PKM2 contribute to cervical cancer cell proliferation induced by the HPV16 E7 oncoprotein. Viruses. 2021;13(3). 10.3390/v13030433 PMC8001101 33800513

[148] Go SH , Rho SB , Yang DW , Kim BR , Lee CH , Lee SH . HPV‐18 E7 interacts with Elk‐1 leading to elevation of the transcriptional activity of Elk‐1 in cervical cancer. Biomol Ther Seoul. 2022;30(6):593‐602. 36305294 10.4062/biomolther.2022.108 PMC9622318

[149] Wang Y , Wang J , Liu C , Li M . Silent information regulator 1 promotes proliferation, migration, and invasion of cervical cancer cells and is upregulated by human papillomavirus 16 E7 oncoprotein. Gynecol Obstet Invest. 2022;87(1):22‐29. 34808628 10.1159/000520642

[150] Miller J , Dakic A , Spurgeon M , et al. AIB1 is a novel target of the high‐risk HPV E6 protein and a biomarker of cervical cancer progression. J Med Virol. 2022;94(8):3962‐3977. 35437795 10.1002/jmv.27795 PMC9199254

[151] Wongjampa W , Ekalaksananan T , Chopjitt P , et al. Suppression of miR‐22, a tumor suppressor in cervical cancer, by human papillomavirus 16 E6 via a p53/miR‐22/HDAC6 pathway. PLoS One. 2018;13(10):e0206644. 30379969 10.1371/journal.pone.0206644 PMC6209303

[152] Jayamohan S , Kannan M , Moorthy RK , et al. Dysregulation of miR‐375/AEG‐1 axis by human papillomavirus 16/18‐E6/E7 promotes cellular proliferation, migration, and invasion in cervical cancer. Front Oncol. 2019;9:847. 31552174 10.3389/fonc.2019.00847 PMC6746205

[153] Cui X , Wang X , Zhou X , Jia J , Chen H , Zhao W . miR‐106a regulates cell proliferation and autophagy by targeting LKB1 in HPV‐16‐associated cervical cancer. Mol Cancer Res. 2020;18(8):1129‐1141. 32345599 10.1158/1541-7786.MCR-19-1114

[154] Li B , Guo X , Li N , et al. WNT1, a target of miR‐34a, promotes cervical squamous cell carcinoma proliferation and invasion by induction of an E‐P cadherin switch via the WNT/β‐catenin pathway. Cell Oncol Dordr. 2020;43(3):489‐503. 32301035 10.1007/s13402-020-00506-8 PMC7214512

[155] Morgan EL , Patterson MR , Ryder EL , et al. MicroRNA‐18a targeting of the STK4/MST1 tumour suppressor is necessary for transformation in HPV positive cervical cancer. PLoS Pathog. 2020;16(6):e1008624. 32555725 10.1371/journal.ppat.1008624 PMC7326282

[156] Huang J , Liu R , Zhang Y , Sheng X . Mechanistic analysis of endothelial lipase G promotion of the occurrence and development of cervical carcinoma by activating the phosphatidylinositol‐4,5‐bisphosphate 3‐kinase/protein kinase B/mechanistic target of rapamycin kinase signalling pathway. J Obstet Gynaecol. 2023;43(1):2151353. 36606668 10.1080/01443615.2022.2151353

[157] Wang H , Hu H , Luo Z , et al. miR‐4454 up‐regulated by HPV16 E6/E7 promotes invasion and migration by targeting ABHD2/NUDT21 in cervical cancer. Biosci Rep. 2020;40(9). 10.1042/BSR20200796 PMC7468098 32816024

[158] Liu Z , Mao L , Wang L , Zhang H , Hu X . miR‑218 functions as a tumor suppressor gene in cervical cancer. Mol Med Rep. 2020;21(1):209‐219. 31746391 10.3892/mmr.2019.10809 PMC6896272

[159] Qu X , Li Y , Wang L , Yuan N , Ma M , Chen Y . LncRNA SNHG8 accelerates proliferation and inhibits apoptosis in HPV‐induced cervical cancer through recruiting EZH2 to epigenetically silence RECK expression. J Cell Biochem. 2020;121(10):4120‐4129. 31961005 10.1002/jcb.29646

[160] Wang J , Xiang F , Liu X , et al. HPV E7 affects the function of cervical cancer cells via the TAL1/lnc‑EBIC/KLHDC7B axis. Oncol Rep. 2021;45(5). 10.3892/or.2021.8002 33760214

[161] Yan G , Wan Z , Zhong Y , Chen M . LncRNA MCM3AP‐AS1 regulates miR‐21/PTEN axis to affect cervical squamous cell carcinoma cell proliferation and apoptosis. Crit Rev Eukaryot Gene Expr. 2022;32(4):49‐56. 10.1615/CritRevEukaryotGeneExpr.2022041014 35695665

[162] Lai SY , Guan HM , Liu J , et al. Long noncoding RNA SNHG12 modulated by human papillomavirus 16 E6/E7 promotes cervical cancer progression via ERK/Slug pathway. J Cell Physiol. 2020;235(11):7911‐7922. 31943193 10.1002/jcp.29446

[163] Wang T , Zhang W , Huang W , Hua Z , Li S . LncRNA MALAT1 was regulated by HPV16 E7 independently of pRB in cervical cancer cells. J Cancer. 2021;12(21):6344‐6355. 34659524 10.7150/jca.61194 PMC8489136

[164] Hao Y , Yan Z , Zhang A , et al. IL‐6/STAT3 mediates the HPV18 E6/E7 stimulated upregulation of MALAT1 gene in cervical cancer HeLa cells. Virus Res. 2020;281:197907. 32113834 10.1016/j.virusres.2020.197907

[165] Ou R , Lv M , Liu X , et al. HPV16 E6 oncoprotein‐induced upregulation of lncRNA GABPB1‐AS1 facilitates cervical cancer progression by regulating miR‐519e‐5p/Notch2 axis. Faseb J. 2020;34(10):13211‐13223. 32844486 10.1096/fj.202000762R

[166] Barr JA , Hayes KE , Brownmiller T , et al. Long non‐coding RNA FAM83H‐AS1 is regulated by human papillomavirus 16 E6 independently of p53 in cervical cancer cells. Sci Rep. 2019;9(1):3662. 30842470 10.1038/s41598-019-40094-8 PMC6403315

[167] Fontan CT , Prabhakar AT , Wang X , et al. Human papillomavirus 16 E2 blocks cellular senescence in response to activation of the DNA damage response. Virology. 2022;575:54‐62. 36058086 10.1016/j.virol.2022.08.007 PMC10715573

[168] Villota C , Varas‐Godoy M , Jeldes E , et al. HPV‐18 E2 protein downregulates antisense noncoding mitochondrial RNA‐2, delaying replicative senescence of human keratinocytes. Aging. 2018;11(1):33‐47. 30595560 10.18632/aging.101711 PMC6339806

[169] Shimada M , Yamashita A , Saito M , et al. The human papillomavirus E6 protein targets apoptosis‐inducing factor (AIF) for degradation. Sci Rep. 2020;10(1):14195. 32848167 10.1038/s41598-020-71134-3 PMC7450093

[170] Tang S , Ding S , Yu L , Shen H , Wan Y , Wu Y . Effects of HPV16 E6 protein on Daxx‐induced apoptosis in C33A cells. Cell Mol Biol Lett. 2020;25:38. 32782452 10.1186/s11658-020-00230-z PMC7414724

[171] Wang Y , Liu R , Liao J , et al. Orthogonal ubiquitin transfer reveals human papillomavirus E6 downregulates nuclear transport to disarm interferon‐γ dependent apoptosis of cervical cancer cells. Faseb J. 2021;35(11):e21986. 34662469 10.1096/fj.202101232RR

[172] Lee HJ , Kim MJ , Kim YS , Choi MY , Cho GJ , Choi WS . UHRF1 silences gelsolin to inhibit cell death in early stage cervical cancer. Biochem Biophys Res Commun. 2020;526(4):1061‐1068. 32312517 10.1016/j.bbrc.2020.03.185

[173] Liu X , Ma H , Fei L , et al. HPV‐mediated down‐regulation of NOD1 inhibits apoptosis in cervical cancer. Infect Agent Cancer. 2020;15:6. 32021648 10.1186/s13027-020-0272-3 PMC6993450

[174] Guan HM , Li WQ , Liu J , Zhou JY . LncRNA HIF1A‐AS2 modulated by HPV16 E6 regulates apoptosis of cervical cancer cells via P53/caspase9/caspase3 axis. Cell Signal. 2022;97:110390. 35728704 10.1016/j.cellsig.2022.110390

[175] Cararo‐Lopes E , Dias MH , da Silva MS , et al. Autophagy buffers Ras‐induced genotoxic stress enabling malignant transformation in keratinocytes primed by human papillomavirus. Cell Death Dis. 2021;12(2):194. 33602932 10.1038/s41419-021-03476-3 PMC7892846

[176] Tingting C , Shizhou Y , Songfa Z , et al. Human papillomavirus 16E6/E7 activates autophagy via Atg9B and LAMP1 in cervical cancer cells. Cancer Med. 2019;8(9):4404‐4416. 31215164 10.1002/cam4.2351 PMC6675746

[177] Zhang B , Song Y , Sun S , et al. Human papillomavirus 11 early protein E6 activates autophagy by repressing AKT/mTOR and Erk/mTOR. J Virol. 2019;93(12). 10.1128/JVI.00172-19 PMC6613752 30971468

[178] Hua C , Zheng Q , Zhu J , et al. Human papillomavirus type 16 early protein E7 activates autophagy through inhibition of dual‐specificity phosphatase 5. Oxid Med Cell Longev. 2022;2022:1863098. 35368866 10.1155/2022/1863098 PMC8966754

[179] Bober P , Tkáčiková S , Talian I , Urdzík P , Toporcerová S , Sabo J . Differential urinary proteomic analysis of high‐risk cervical intraepithelial neoplasia. Int J Mol Sci. 2023;24(3). 10.3390/ijms24032531 PMC9916937 36768853

[180] Guo X , Dou Y , Liu S , et al. Elevated expression of ADAM10 induced by HPV E6 influences the prognosis of cervical cancer. Genet Test Mol Biomark. 2023;27(5):165‐171. 10.1089/gtmb.2022.0170 37257180

[181] Mendonça F , Teles AM , Nascimento MDDSB , et al. Human papillomavirus modulates matrix metalloproteinases during carcinogenesis: clinical significance and role of viral oncoproteins. In Vivo. 2022;36(6):2531‐2541. 36309355 10.21873/invivo.12990 PMC9677770

[182] Lu Y , Chen Y , Zhang Z , et al. HPV16 E6 promotes cell proliferation, migration, and invasion of human cervical cancer cells by elevating both EMT and stemness characteristics. Cell Biol Int. 2022;46(4):599‐610. 34957655 10.1002/cbin.11756

[183] Farzanehpour M , Faghihloo E , Salimi V , et al. Comparison of Snail1, ZEB1, E‐cadherin expression levels in HPV‐induced cervical cancer. Iran J Public Health. 2020;49(11):2179‐2188. 33708739 10.18502/ijph.v49i11.4736 PMC7917501

[184] Ranieri D , French D , Raffa S , Guttieri L , Torrisi MR , Belleudi F . Expression of the E5 oncoprotein of HPV16 impacts on the molecular profiles of EMT‐related and differentiation genes in ectocervical low‐grade lesions. Int J Mol Sci. 2021;22(12). 10.3390/ijms22126534 PMC8235634 34207106

[185] Rosendo‐Chalma P , Antonio‐Vejar V , Bigoni‐Ordóñez GD , Patiño‐Morales CC , Cano‐García A , García‐Carrancá A . CDH1 and SNAI1 are regulated by E7 from human papillomavirus types 16 and 18. Int J Oncol. 2020;57(1):301‐313. 32319591 10.3892/ijo.2020.5039

[186] Wang Q , Song R , Zhao C , et al. HPV16 E6 promotes cervical cancer cell migration and invasion by downregulation of NHERF1. Int J Cancer. 2019;144(7):1619‐1632. 30230542 10.1002/ijc.31876

[187] Matarrese P , Vona R , Ascione B , Paggi MG , Mileo AM . Physical interaction between HPV16E7 and the actin‐binding protein gelsolin regulates epithelial‐mesenchymal transition via HIPPO‐YAP axis. Cancers Basel. 2021;13(2). 10.3390/cancers13020353 PMC7836002 33477952

[188] Huang Y , Liu R , Han X , Hou X , Tian Y , Zhang W . Rab31 promotes the invasion and metastasis of cervical cancer cells by inhibiting MAPK6 degradation. Int J Biol Sci. 2022;18(1):112‐123. 34975321 10.7150/ijbs.63388 PMC8692139

[189] Wang A , Krawczyk E , et al. Overexpression of the telomerase holoenzyme induces EMT and tumorigenesis of HPV‐immortalized keratinocytes. J Med Virol. 2023;95(4):e28681. 36929719 10.1002/jmv.28681

[190] Hu J , Liao D , Sun Z , et al. The HPV16 E6, E7/miR‐23b‐3p/ICAT signaling axis promotes proliferation, migration, invasion and EMT of cervical cancer cells. Carcinogenesis. 2023;44(3):221‐231. 36847693 10.1093/carcin/bgad008

[191] Wang H , Wei M , Kang Y , Xing J , Zhao Y . Circular RNA circ_PVT1 induces epithelial‐mesenchymal transition to promote metastasis of cervical cancer. Aging. 2020;12(20):20139‐20151. 33109773 10.18632/aging.103679 PMC7655209

[192] Akgül B , Kirschberg M , Storey A , Hufbauer M . Human papillomavirus type 8 oncoproteins E6 and E7 cooperate in downregulation of the cellular checkpoint kinase‐1. Int J Cancer. 2019;145(3):797‐806. 30786016 10.1002/ijc.32223

[193] Alfaro‐Mora Y , Domínguez‐Gómez G , Cáceres‐Gutiérrez RE , et al. MPS1 is involved in the HPV16‐E7‐mediated centrosomes amplification. Cell Div. 2021;16(1):6. 34736484 10.1186/s13008-021-00074-9 PMC8567613

[194] Sund DT , Brouwer AF , Walline HM , et al. Understanding the mechanisms of HPV‐related carcinogenesis: implications for cell cycle dynamics. J Theor Biol. 2022;551‐552:111235. 10.1016/j.jtbi.2022.111235 PMC9838640 35973606

[195] Cosper PF , Hrycyniak LCF , Paracha M , et al. HPV16 E6 induces chromosomal instability due to polar chromosomes caused by E6AP‐dependent degradation of the mitotic kinesin CENP‐E. Proc Natl Acad Sci USA. 2023;120(14):e2216700120. 36989302 10.1073/pnas.2216700120 PMC10083562

[196] Mehta K , Laimins L . High‐risk human papillomaviruses and DNA repair. Recent Results Cancer Res. 2021;217:141‐155. 33200365 10.1007/978-3-030-57362-1_7

[197] Uehara K , Tanabe Y , Hirota S , et al. Co‐expression of low‐risk HPV E6/E7 and EBV LMP‐1 leads to precancerous lesions by DNA damage. BMC Cancer. 2021;21(1):688. 34112111 10.1186/s12885-021-08397-0 PMC8194219

[198] Porter VL , Marra MA . The drivers, mechanisms, and consequences of genome instability in HPV‐driven cancers. Cancers Basel. 2022;14(19). 10.3390/cancers14194623 PMC9564061 36230545

[199] Katerji M , Duerksen‐Hughes PJ . DNA damage in cancer development: special implications in viral oncogenesis. Am J Cancer Res. 2021;11(8):3956‐3979. 34522461 PMC8414375

[200] Li W , Tian S , Wang P , et al. The characteristics of HPV integration in cervical intraepithelial cells. J Cancer. 2019;10(12):2783‐2787. 31258786 10.7150/jca.31450 PMC6584928

[201] Nkili‐Meyong AA , Moussavou‐Boundzanga P , Labouba I , et al. Genome‐wide profiling of human papillomavirus DNA integration in liquid‐based cytology specimens from a Gabonese female population using HPV capture technology. Sci Rep. 2019;9(1):1504. 30728408 10.1038/s41598-018-37871-2 PMC6365579

[202] Bouchilloux S , Fer F , Lemée F , et al. Correlation between integration of high‐risk HPV genome into human DNA detected by molecular combing and the severity of cervical lesions: first results of the EXPL‐HPV‐002 study. Ceska Gynekol. 2019;84(2):84‐92. 31238677

[203] Hoyer H , Mehlhorn G , Scheungraber C , et al. Evaluation of integrated HPV DNA as individualized biomarkers for the detection of recurrent CIN2/3 during post‐treatment surveillance. Cancers Basel. 2021;13(13). 10.3390/cancers13133309 PMC8269020 34282754

[204] Li J , Zhang X , Wang P , Li W . The help of HPV integration testing to avoid the misdiagnosis of a patient with stage Ia1 cervical cancer: a case report and literature review. Pharmgenomics Med. 2021;14:1457‐1461. 10.2147/PGPM.S310345 PMC8608241 34819743

[205] Kiseleva VI , Mkrtchyan LS , Ivanov SA , et al. The presence of human papillomavirus DNA integration is associated with poor clinical results in patients with third‐stage cervical cancer. Bull Exp Biol Med. 2019;168(1):87‐91. 31768781 10.1007/s10517-019-04654-2

[206] Yang‐Chun F , Sen‐Yu W , Yuan Z , Yan‐Chun H . Genome‐wide profiling of human papillomavirus DNA integration into human genome and its influence on PD‐L1 expression in chinese uygur cervical cancer women. J Immunol Res. 2020;2020:6284960. 32411801 10.1155/2020/6284960 PMC7204091

[207] Garza‐Rodríguez ML , Oyervides‐Muñoz MA , Pérez‐Maya AA , et al. Analysis of HPV integrations in mexican pre‐tumoral cervical lesions reveal centromere‐enriched breakpoints and abundant unspecific HPV regions. Int J Mol Sci. 2021;22(6). 10.3390/ijms22063242 PMC8005155 33810183

[208] Hu Z , Zhu D , Wang W , et al. Genome‐wide profiling of HPV integration in cervical cancer identifies clustered genomic hot spots and a potential microhomology‐mediated integration mechanism. Nat Genet. 2015;47(2):158‐163. 25581428 10.1038/ng.3178

[209] Li W , Qi Y , Cui X , et al. Characteristic of HPV integration in the genome and transcriptome of cervical cancer tissues. Biomed Res Int. 2018;2018:6242173. 30018982 10.1155/2018/6242173 PMC6029443

[210] Cao C , Hong P , Huang X , et al. HPV‐CCDC106 integration alters local chromosome architecture and hijacks an enhancer by three‐dimensional genome structure remodeling in cervical cancer. J Genet Genomics. 2020;47(8):437‐450. 33023834 10.1016/j.jgg.2020.05.006

[211] Kamal M , Lameiras S , Deloger M , et al. Human papilloma virus (HPV) integration signature in cervical cancer: identification of MACROD2 gene as HPV hot spot integration site. Br J Cancer. 2021;124(4):777‐785. 33191407 10.1038/s41416-020-01153-4 PMC7884736

[212] Akagi K , Li J , Broutian TR , et al. Genome‐wide analysis of HPV integration in human cancers reveals recurrent, focal genomic instability. Genome Res. 2014;24(2):185‐199. 24201445 10.1101/gr.164806.113 PMC3912410

[213] Schrank TP , Kim S , Rehmani H , et al. Direct comparison of HPV16 viral genomic integration, copy loss, and structural variants in oropharyngeal and uterine cervical cancers reveal distinct relationships to E2 disruption and somatic alteration. Cancers Basel. 2022;14(18). 10.3390/cancers14184488 PMC9496734 36139648

[214] Fan J , Fu Y , Peng W , et al. Multi‐omics characterization of silent and productive HPV integration in cervical cancer. Cell Genom. 2023;3(1):100211. 36777180 10.1016/j.xgen.2022.100211 PMC9903858

[215] Zhi W , Wei Y , Lazare C , et al. HPV‐CCDC106 integration promotes cervical cancer progression by facilitating the high expression of CCDC106 after HPV E6 splicing. J Med Virol. 2023;95(1):e28009. 35854676 10.1002/jmv.28009 PMC9796641

[216] Liu M , Han Z , Zhi Y , et al. Long‐read sequencing reveals oncogenic mechanism of HPV‐human fusion transcripts in cervical cancer. Transl Res. 2023;253:80‐94. 36223881 10.1016/j.trsl.2022.09.004

[217] Warburton A , Markowitz TE , Katz JP , Pipas JM , McBride AA . Recurrent integration of human papillomavirus genomes at transcriptional regulatory hubs. NPJ Genom Med. 2021;6(1):101. 34848725 10.1038/s41525-021-00264-y PMC8632991

[218] Tian R , Huang Z , Li L , et al. HPV integration generates a cellular super‐enhancer which functions as ecDNA to regulate genome‐wide transcription. Nucleic Acids Res. 2023;51(9):4237‐4251. 36864748 10.1093/nar/gkad105 PMC10201430

[219] Brimer N , Vande Pol S . Human papillomavirus type 16 E6 induces cell competition. PLoS Pathog. 2022;18(3):e1010431. 35320322 10.1371/journal.ppat.1010431 PMC8979454

[220] Karimzadeh M , Arlidge C , Rostami A , Lupien M , Bratman SV , Hoffman MM . Human papillomavirus integration transforms chromatin to drive oncogenesis. Genome Biol. 2023;24(1):142. 37365652 10.1186/s13059-023-02926-9 PMC10294493

[221] Adeel MM , Jiang H , Arega Y , et al. Structural variations of the 3D genome architecture in cervical cancer development. Front Cell Dev Biol. 2021;9:706375. 34368157 10.3389/fcell.2021.706375 PMC8344058

[222] Wang X , Jia W , Wang M , et al. Human papillomavirus integration perspective in small cell cervical carcinoma. Nat Commun. 2022;13(1):5968. 36216793 10.1038/s41467-022-33359-w PMC9550834

[223] Li W , Lei W , Chao X , et al. Genomic alterations caused by HPV integration in a cohort of Chinese endocervical adenocarcinomas. Cancer Gene Ther. 2021;28(12):1353‐1364. 33398034 10.1038/s41417-020-00283-4 PMC8636260

[224] Groves IJ , Drane ELA , Michalski M , et al. Short‐ and long‐range cis interactions between integrated HPV genomes and cellular chromatin dysregulate host gene expression in early cervical carcinogenesis. PLoS Pathog. 2021;17(8):e1009875. 34432858 10.1371/journal.ppat.1009875 PMC8439666

[225] Zeng X , Wang Y , Liu B , et al. Multi‐omics data reveals novel impacts of human papillomavirus integration on the epigenomic and transcriptomic signatures of cervical tumorigenesis. J Med Virol. 2023;95(5):e28789. 37212325 10.1002/jmv.28789

[226] Brant AC , Menezes AN , Felix SP , de Almeida LM , Sammeth M , Moreira MAM . Characterization of HPV integration, viral gene expression and E6E7 alternative transcripts by RNA‐Seq: a descriptive study in invasive cervical cancer. Genomics. 2019;111(6):1853‐1861. 30552977 10.1016/j.ygeno.2018.12.008

[227] Iden M , Tsaih SW , Huang YW , et al. Multi‐omics mapping of human papillomavirus integration sites illuminates novel cervical cancer target genes. Br J Cancer. 2021;125(10):1408‐1419. 34526665 10.1038/s41416-021-01545-0 PMC8575955

[228] Xiong J , Cheng J , Shen H , et al. Detection of HPV and human chromosome sites by dual‐color fluorescence in situ hybridization reveals recurrent HPV integration sites and heterogeneity in cervical cancer. Front Oncol. 2021;11:734758. 34676167 10.3389/fonc.2021.734758 PMC8523950

[229] Zhou L , Qiu Q , Zhou Q , et al. Long‐read sequencing unveils high‐resolution HPV integration and its oncogenic progression in cervical cancer. Nat Commun. 2022;13(1):2563. 35538075 10.1038/s41467-022-30190-1 PMC9091225

[230] Li X , Ren C , Huang A , et al. PIBF1 regulates multiple gene expression via impeding long‐range chromatin interaction to drive the malignant transformation of HPV16 integration epithelial cells. J Adv Res. 2023;S2090‐1232(23)00121‐2.10.1016/j.jare.2023.04.01537182685

[231] Zhao J , Zheng W , Wang L , et al. Human papillomavirus (HPV) integration signature in cervical lesions: identification of MACROD2 gene as HPV hot spot integration site. Arch Gynecol Obstet. 2023;307(4):1115‐1123. 36008642 10.1007/s00404-022-06748-1

[232] Reyna‐Hernández MA , Alarcón‐Romero LDC , Ortiz‐Ortiz J , et al. GLUT1, LDHA, and MCT4 expression is deregulated in cervical cancer and precursor lesions. J Histochem Cytochem. 2022;70(6):437‐446. 35615882 10.1369/00221554221101662 PMC9169107

[233] Ma D , Huang Y , Song S . Inhibiting the HPV16 oncogene‐mediated glycolysis sensitizes human cervical carcinoma cells to 5‐fluorouracil. Onco Targets Ther. 2019;12:6711‐6720. 31695407 10.2147/OTT.S205334 PMC6707439

[234] Kirschberg M , Heuser S , Marcuzzi GP , et al. ATP synthase modulation leads to an increase of spare respiratory capacity in HPV associated cancers. Sci Rep. 2020;10(1):17339. 33060693 10.1038/s41598-020-74311-6 PMC7567072

[235] Gu Z , Zhang H , Guo X , Cao Y . Enhanced glycogen metabolism supports the survival and proliferation of HPV‐infected keratinocytes in condylomata acuminata. J Invest Dermatol. 2020;140(8):1513‐1523. .e5. 32004566 10.1016/j.jid.2020.01.010

[236] Sitarz K , Czamara K , Bialecka J , et al. HPV infection significantly accelerates glycogen metabolism in cervical cells with large nuclei: Raman microscopic study with subcellular resolution. Int J Mol Sci. 2020;21(8). 10.3390/ijms21082667 PMC7215571 32290479

[237] Hu C , Liu T , Han C , et al. HPV E6/E7 promotes aerobic glycolysis in cervical cancer by regulating IGF2BP2 to stabilize m(6)A‐MYC expression. Int J Biol Sci. 2022;18(2):507‐521. 35002506 10.7150/ijbs.67770 PMC8741847

[238] Prakasam G , Iqbal MA , Srivastava A , Bamezai RNK , Singh RK . HPV18 oncoproteins driven expression of PKM2 reprograms HeLa cell metabolism to maintain aerobic glycolysis and viability. Virusdisease. 2022;33(3):223‐235. 36277414 10.1007/s13337-022-00776-w PMC9481809

[239] Liu S , Song L , Yao H , Zhang L . HPV16 E6/E7 stabilize PGK1 protein by reducing its poly‐ubiquitination in cervical cancer. Cell Biol Int. 2022;46(3):370‐380. 34882921 10.1002/cbin.11744

[240] Ortiz‐Pedraza Y , Muñoz‐Bello JO , Ramos‐Chávez LA , et al. HPV16 E6 and E7 oncoproteins stimulate the glutamine pathway maintaining cell proliferation in a SNAT1‐dependent fashion. Viruses. 2023;15(2). 10.3390/v15020324 PMC9964736 36851539

[241] Ebrahimi S , Soltani A , Hashemy SI . Oxidative stress in cervical cancer pathogenesis and resistance to therapy. J Cell Biochem. 2019;120(5):6868‐6877. 30426530 10.1002/jcb.28007

[242] Hochmann J , Parietti F , Martínez J , et al. Human papillomavirus type 18 E5 oncoprotein cooperates with E6 and E7 in promoting cell viability and invasion and in modulating the cellular redox state. Mem Inst Oswaldo Cruz. 2020;115:e190405. 32187327 10.1590/0074-02760190405 PMC7066992

[243] Qiu JJ , Sun SG , Tang XY , Lin YY , Hua KQ . Extracellular vesicular Wnt7b mediates HPV E6‐induced cervical cancer angiogenesis by activating the β‐catenin signaling pathway. J Exp Clin Cancer Res. 2020;39(1):260. 33234148 10.1186/s13046-020-01745-1 PMC7687741

[244] Rho SB , Lee SH , Byun HJ , Kim BR , Lee CH . IRF‐1 inhibits angiogenic activity of HPV16 E6 oncoprotein in cervical cancer. Int J Mol Sci. 2020;21(20). 10.3390/ijms21207622 PMC7589982 33076322

[245] Mendling W. Vaginal microbiota. Adv Exp Med Biol. 2016;902:83‐93. 27161352 10.1007/978-3-319-31248-4_6

[246] Smith SB , Ravel J . The vaginal microbiota, host defence and reproductive physiology. J Physiol. 2017;595(2):451‐463. 27373840 10.1113/JP271694 PMC5233653

[247] Stapleton AE . The vaginal microbiota and urinary tract infection. Microbiol Spectr. 2016;4(6). 10.1128/microbiolspec.UTI-0025-2016 PMC5746606 28087949

[248] Xu X , Rao H , Fan X , et al. HPV‐related cervical diseases: alteration of vaginal microbiotas and promising potential for diagnosis. J Med Virol. 2023;95(1):e28351. 36437385 10.1002/jmv.28351

[249] Andrade Pessoa Morales J , Marconi C , El‐Zein M , et al. Vaginal microbiome components as correlates of cervical human papillomavirus infection. J Infect Dis. 2022;226(6):1084‐1097. 34718662 10.1093/infdis/jiab547

[250] Sasivimolrattana T , Chantratita W , Sensorn I , et al. Cervical microbiome in women infected with HPV16 and high‐risk HPVs. Int J Env Res Public Health. 2022;19(22). 10.3390/ijerph192214716 PMC9690271 36429432

[251] Usyk M , Zolnik CP , Castle PE , et al. Cervicovaginal microbiome and natural history of HPV in a longitudinal study. PLoS Pathog. 2020;16(3):e1008376. 32214382 10.1371/journal.ppat.1008376 PMC7098574

[252] Teka B , Yoshida‐Court K , Firdawoke E , et al. Cervicovaginal microbiota profiles in precancerous lesions and cervical cancer among Ethiopian women. Microorganisms. 2023;11(4). 10.3390/microorganisms11040833 PMC10144031 37110255

[253] Gomez Cherey JF , Payalef SN , Fleider L , et al. Microbiota unbalance in relation to high‐risk human papillomavirus cervical infection. Int J Gynecol Cancer. 2023;33(4):482‐488. 36604120 10.1136/ijgc-2022-003760

[254] Wei ZT , Chen HL , Wang CF , Yang GL , Han SM , Zhang SL . Depiction of vaginal microbiota in women with high‐risk human papillomavirus infection. Front Public Health. 2020;8:587298. 33490017 10.3389/fpubh.2020.587298 PMC7820762

[255] Cheng L , Norenhag J , Hu YOO , et al. Vaginal microbiota and human papillomavirus infection among young Swedish women. npj Biofilms Microbiomes. 2020;6(1):39. 33046723 10.1038/s41522-020-00146-8 PMC7552401

[256] Camargo M , Vega L , Muñoz M , et al. Changes in the cervical microbiota of women with different high‐risk human papillomavirus loads. Viruses. 2022;14(12). 10.3390/v14122674 PMC9781391 36560678

[257] Huang J , Yin C , Wang J . Relationship between vaginal microecological changes and oncogene E6/E7 and high‐risk human papillomavirus infection. J Obstet Gynaecol. 2023;43(1):2161349. 36645341 10.1080/01443615.2022.2161349

[258] Ferreira CST , Marconi C , Parada CMGL , Ravel J , da Silva MG . Sialidase activity in the cervicovaginal fluid is associated with changes in bacterial components of lactobacillus‐deprived microbiota. Front Cell Infect Microbiol. 2021;11:813520. 35096658 10.3389/fcimb.2021.813520 PMC8793624

[259] Ragaliauskas T , Plečkaitytė M , Jankunec M , Labanauskas L , Baranauskiene L , Valincius G . Inerolysin and vaginolysin, the cytolysins implicated in vaginal dysbiosis, differently impair molecular integrity of phospholipid membranes. Sci Rep. 2019;9(1):10606. 31337831 10.1038/s41598-019-47043-5 PMC6650466

[260] Borgogna JLC , Shardell MD , Grace SG , et al. Biogenic amines increase the odds of bacterial vaginosis and affect the growth of and lactic acid production by vaginal Lactobacillus spp. Appl Environ Microb. 2021;87(10):e03068‐20. 10.1128/AEM.03068-20 PMC8117770 33674429

[261] Chao X , Sun T , Wang S , et al. Research of the potential biomarkers in vaginal microbiome for persistent high‐risk human papillomavirus infection. Ann Transl Med. 2020;8(4):100. 32175393 10.21037/atm.2019.12.115 PMC7049000

[262] Ritu W , Enqi W , Zheng S , Wang J , Ling Y , Wang Y . Evaluation of the associations between cervical microbiota and HPV infection, clearance, and persistence in cytologically normal women. Cancer Prev Res Phila. 2019;12(1):43‐56. 30463989 10.1158/1940-6207.CAPR-18-0233

[263] Di Paola M , Sani C , Clemente AM , et al. Characterization of cervico‐vaginal microbiota in women developing persistent high‐risk Human Papillomavirus infection. Sci Rep. 2017;7(1):10200. 28860468 10.1038/s41598-017-09842-6 PMC5579045

[264] Usyk M , Schlecht NF , Pickering S , et al. molBV reveals immune landscape of bacterial vaginosis and predicts human papillomavirus infection natural history. Nat Commun. 2022;13(1):233. 35017496 10.1038/s41467-021-27628-3 PMC8752746

[265] Nicolò S , Tanturli M , Mattiuz G , et al. Vaginal Lactobacilli and vaginal dysbiosis‐associated bacteria differently affect cervical epithelial and immune homeostasis and anti‐viral defenses. Int J Mol Sci. 2021;22(12):6487. 34204294 10.3390/ijms22126487 PMC8234132

[266] Gosmann C , Mattarollo SR , Bridge JA , Frazer IH , Blumenthal A . IL‐17 suppresses immune effector functions in human papillomavirus‐associated epithelial hyperplasia. J Immunol. 2014;193(5):2248‐2257. 25063870 10.4049/jimmunol.1400216 PMC4135400

[267] Machado A , Jefferson KK , Cerca N . Interactions between Lactobacillus crispatus and bacterial vaginosis (BV)‐associated bacterial species in initial attachment and biofilm formation. Int J Mol Sci. 2013;14(6):12004‐12012. 23739678 10.3390/ijms140612004 PMC3709769

[268] Hardy L , Cerca N , Jespers V , Vaneechoutte M , Crucitti T . Bacterial biofilms in the vagina. Res Microbiol. 2017;168(9‐10):865‐874. 28232119 10.1016/j.resmic.2017.02.001

[269] Yang Z , Zhang Y , Stubbe‐Espejel A , et al. Vaginal microbiota and personal risk factors associated with HPV status conversion‐A new approach to reduce the risk of cervical cancer? PLoS One. 2022;17(8):e0270521. 35944043 10.1371/journal.pone.0270521 PMC9362946

[270] Dong B , Huang Y , Cai H , et al. Prevotella as the hub of the cervicovaginal microbiota affects the occurrence of persistent human papillomavirus infection and cervical lesions in women of childbearing age via host NF‐κB/C‐myc. J Med Virol. 2022;94(11):5519‐5534. 35835717 10.1002/jmv.28001

[271] Nicolò S , Antonelli A , Tanturli M , et al. Bacterial species from vaginal microbiota differently affect the production of the E6 and E7 oncoproteins and of p53 and p‐Rb oncosuppressors in HPV16‐infected cells. Int J Mol Sci. 2023;24(8). 10.3390/ijms24087173 PMC10138431 37108333

[272] Li C , Li Y , Sui L , Wang J , Li F . Phenyllactic acid promotes cell migration and invasion in cervical cancer via IKK/NF‐κB‐mediated MMP‐9 activation. Cancer Cell Int. 2019;19:241. 31572058 10.1186/s12935-019-0965-0 PMC6757389

[273] Challagundla N , Chrisophe‐Bourdon J , Agrawal‐Rajput R . Chlamydia trachomatis infection co‐operatively enhances HPV E6‐E7 oncogenes mediated tumorigenesis and immunosuppression. Microb Pathog. 2023;175:105929. 36565970 10.1016/j.micpath.2022.105929

[274] Khan AA , A. A Abuderman , Ashraf MT , Khan Z . Protein‐protein interactions of HPV‐Chlamydia trachomatis‐human and their potential in cervical cancer. Future Microbiol. 2020;15:509‐520. A. 32476479 10.2217/fmb-2019-0242

[275] A D , Bi H , Zhang D , Xiao B . Association between human papillomavirus infection and common sexually transmitted infections, and the clinical significance of different Mycoplasma subtypes. Front Cell Infect Microbiol. 2023;13:1145215. 37009504 10.3389/fcimb.2023.1145215 PMC10061082

[276] Liu H , Liang H , Li D , Wang M , Li Y . Association of cervical dysbacteriosis, HPV oncogene expression, and cervical lesion progression. Microbiol Spectr. 2022;10(5):e0015122. 36036584 10.1128/spectrum.00151-22 PMC9602310

[277] Mitra A , MacIntyre DA , Ntritsos G , et al. The vaginal microbiota associates with the regression of untreated cervical intraepithelial neoplasia 2 lesions. Nat Commun. 2020;11(1):1999. 32332850 10.1038/s41467-020-15856-y PMC7181700

[278] Tasic D , Lazarevic I , Knezevic A , et al. The impact of environmental and behavioural cofactors on the development of cervical disorders in HR‐HPV‐infected women in Serbia. Epidemiol Infect. 2018;146(13):1714‐1723. 29923470 10.1017/S0950268818001668 PMC9507945

[279] Kuguyo O , Tsikai N , Thomford NE , et al. Genetic susceptibility for cervical cancer in African populations: what are the host genetic drivers? OMICS. 2018;22(7):468‐483. 30004844 10.1089/omi.2018.0075

[280] Chagas BS , Lima RCP , Paiva Júnior SSL , et al. Significant association between IL10‐1082/‐819 and TNF‐308 haplotypes and the susceptibility to cervical carcinogenesis in women infected by Human papillomavirus. Cytokine. 2019;113:99‐104. 29935877 10.1016/j.cyto.2018.06.014

[281] Chauhan A , Pandey N , Desai A , et al. Association of TLR4 and TLR9 gene polymorphisms and haplotypes with cervicitis susceptibility. PLoS One. 2019;14(7):e0220330. 31365550 10.1371/journal.pone.0220330 PMC6668796

[282] Adebamowo SN , Adeyemo AA . Classical HLA alleles are associated with prevalent and persistent cervical high‐risk HPV infection in African women. Hum Immunol. 2019;80(9):723‐730. 31072753 10.1016/j.humimm.2019.04.011 PMC6773487

[283] Okuyama NCM , Cezar‐Dos‐Santos F , Pereira ÉR , et al. Genetic variant in CXCL12 gene raises susceptibility to HPV infection and squamous intraepithelial lesions development: a case‐control study. J Biomed Sci. 2018;25(1):69. 30227860 10.1186/s12929-018-0472-y PMC6145110

[284] Du GH , Wang JK , Richards JR , Wang JJ . Genetic polymorphisms in tumor necrosis factor alpha and interleukin‐10 are associated with an increased risk of cervical cancer. Int Immunopharmacol. 2019;66:154‐161. 30453149 10.1016/j.intimp.2018.11.015 PMC6348885

[285] Muñoz JP , Carrillo‐Beltrán D , Aedo‐Aguilera V , et al. Tobacco exposure enhances human papillomavirus 16 oncogene expression via EGFR/PI3K/Akt/c‐Jun signaling pathway in cervical cancer cells. Front Microbiol. 2018;9:3022. 30619121 10.3389/fmicb.2018.03022 PMC6304352

[286] Chen L , Wang H . eIF4E is a critical regulator of human papillomavirus (HPV)‐immortalized cervical epithelial (H8) cell growth induced by nicotine. Toxicology. 2019;419:1‐10. 30836163 10.1016/j.tox.2019.02.017

[287] Chen L , Wang H . Nicotine promotes human papillomavirus (HPV)‐immortalized cervical epithelial cells (H8) proliferation by activating RPS27a‐Mdm2‐P53 pathway in vitro. Toxicol Sci. 2019;167(2):408‐418. 30272249 10.1093/toxsci/kfy246

[288] Riera‐Leal A , Ramírez De Arellano A , Ramírez‐López IG , et al. Effects of 60 kDa prolactin and estradiol on metabolism and cell survival in cervical cancer: co‑expression of their hormonal receptors during cancer progression. Oncol Rep. 2018;40(6):3781‐3793. 30272319 10.3892/or.2018.6743

[289] Ramírez‐López IG , Ramírez de Arellano A , Jave‐Suárez LF , et al. Interaction between 17β‐estradiol, prolactin and human papillomavirus induce E6/E7 transcript and modulate the expression and localization of hormonal receptors. Cancer Cell Int. 2019;19:227. 31507337 10.1186/s12935-019-0935-6 PMC6720994

[290] Ogawa M , Hashimoto K , Kitano S , et al. Estrogen induces genomic instability in high‐risk HPV‐infected cervix and promotes the carcinogenesis of cervical adenocarcinoma. Biochem Biophys Res Commun. 2023;659:80‐90. 37054506 10.1016/j.bbrc.2023.04.009

[291] Hu J , Brendle SA , Li JJ , et al. Depo medroxyprogesterone (DMPA) promotes papillomavirus infections but does not accelerate disease progression in the anogenital tract of a mouse model. Viruses. 2022;14(5). 10.3390/v14050980 PMC9147738 35632722

[292] Sohn AH , Kerr SJ , Hansudewechakul R , et al. Risk factors for human papillomavirus infection and abnormal cervical cytology among perinatally human immunodeficiency virus‐infected and uninfected Asian youth. Clin Infect Dis. 2018;67(4):606‐613. 29617952 10.1093/cid/ciy144 PMC7190885

[293] Du P . Human papillomavirus infection and cervical cancer in HIV+ women. Cancer Treat Res. 2019;177:105‐129. 30523623 10.1007/978-3-030-03502-0_5

[294] Zhang C , Luo Y , Zhong R , et al. Role of polycyclic aromatic hydrocarbons as a co‐factor in human papillomavirus‐mediated carcinogenesis. BMC Cancer. 2019;19(1):138. 30744599 10.1186/s12885-019-5347-4 PMC6371473

[295] Kuebler U , Fischer S , Mernone L , Breymann C , Abbruzzese E , Ehlert U . Is stress related to the presence and persistence of oncogenic human papillomavirus infection in young women? BMC Cancer. 2021;21(1):419. 33863301 10.1186/s12885-021-08010-4 PMC8052668

[296] Lin HY , Fu Q , Kao YH , et al. Antioxidants associated with oncogenic human papillomavirus infection in women. J Infect Dis. 2021;224(9):1520‐1528. 33735375 10.1093/infdis/jiab148 PMC8599710

[297] Ocadiz‐Delgado R , Serafin‐Higuera N , Alvarez‐Rios E , et al. Vitamin A deficiency in K14E7HPV expressing transgenic mice facilitates the formation of malignant cervical lesions. APMIS Acta Pathol Microbiol Immunol Scand. 2021;129(8):512‐523. 10.1111/apm.13159 34046932

[298] Byun JM , Jeong DH , Kim YN , et al. Persistent HPV‐16 infection leads to recurrence of high‐grade cervical intraepithelial neoplasia. Med Baltim. 2018;97(51):e13606. 10.1097/MD.0000000000013606 PMC6320141 30572469

[299] Boilesen DR , Neckermann P , Willert T , et al. Efficacy and synergy with cisplatin of an adenovirus vectored therapeutic E1E2E6E7 vaccine against HPV genome‐positive C3 cancers in mice. Cancer Immunol Res. 2023;11(2):261‐275. 36534088 10.1158/2326-6066.CIR-22-0174

[300] Wan B , Qin L , Ma W , Wang H . Construction and immune effect of an HPV16/18/58 trivalent therapeutic adenovirus vector vaccine. Infect Agent Cancer. 2022;17(1):5. 35197089 10.1186/s13027-022-00417-3 PMC8867827

[301] Riepler L , Frommelt LS , Wilmschen‐Tober S , et al. Therapeutic efficacy of a VSV‐GP‐based human papilloma virus vaccine in a murine cancer model. J Mol Biol. 2023;435(13):168096. 37086948 10.1016/j.jmb.2023.168096

[302] Pellom ST , Smalley Rumfield C , Morillon YM , et al. Characterization of recombinant gorilla adenovirus HPV therapeutic vaccine PRGN‐2009. JCI Insight. 2021;6(7). 10.1172/jci.insight.141912 PMC8119209 33651712

[303] Jorritsma‐Smit A , van Zanten CJ , Schoemaker J , et al. GMP manufacturing of Vvax001, a therapeutic anti‐HPV vaccine based on recombinant viral particles. Eur J Pharm Sci. 2020;143:105096. 31669389 10.1016/j.ejps.2019.105096

[304] Komdeur FL , Singh A , van de Wall S , et al. First‐in‐human phase I clinical trial of an SFV‐based RNA replicon cancer vaccine against HPV‐induced cancers. Mol Ther. 2021;29(2):611‐625. 33160073 10.1016/j.ymthe.2020.11.002 PMC7854293

[305] Schmidt S , Bonilla WV , Reiter A , et al. Live‐attenuated lymphocytic choriomeningitis virus‐based vaccines for active immunotherapy of HPV16‐positive cancer. Oncoimmunology. 2020;9(1):1809960. 33457095 10.1080/2162402X.2020.1809960 PMC7781782

[306] Beyranvand Nejad E , Ratts RB , Panagioti E , et al. Demarcated thresholds of tumor‐specific CD8 T cells elicited by MCMV‐based vaccine vectors provide robust correlates of protection. J Immunother Cancer. 2019;7(1):25. 30704520 10.1186/s40425-019-0500-9 PMC6357411

[307] Abdelaziz MO , Ossmann S , Kaufmann AM , et al. Development of a human cytomegalovirus (HCMV)‐based therapeutic cancer vaccine uncovers a previously unsuspected viral block of MHC Class I antigen presentation. Front Immunol. 2019;10:1776. 31417555 10.3389/fimmu.2019.01776 PMC6682651

[308] Su L , Zhang Y , Zhang X , et al. Combination immunotherapy with two attenuated Listeria strains carrying shuffled HPV‐16 E6E7 protein causes tumor regression in a mouse tumor model. Sci Rep. 2021;11(1):13404. 34183739 10.1038/s41598-021-92875-9 PMC8238941

[309] Galicia‐Carmona T , Arango‐Bravo E , Serrano‐Olvera JA , et al. ADXS11‐001 LM‐LLO as specific immunotherapy in cervical cancer. Hum Vaccin Immunother. 2021;17(8):2617‐2625. 33793380 10.1080/21645515.2021.1893036 PMC8475562

[310] Taghinezhad SS , Mohseni AH , Keyvani H , Razavi MR . Phase 1 safety and immunogenicity trial of recombinant lactococcus lactis expressing human papillomavirus type 16 E6 oncoprotein vaccine. Mol Ther Methods Clin Dev. 2019;15:40‐51. 31649954 10.1016/j.omtm.2019.08.005 PMC6804834

[311] Ikeda Y , Adachi K , Tomio K , et al. A placebo‐controlled, double‐blind randomized (Phase IIB) trial of oral administration with HPV16 E7‐expressing Lactobacillus, GLBL101c, for the treatment of cervical intraepithelial neoplasia grade 2 (CIN2). Vaccines Basel. 2021;9(4). 10.3390/vaccines9040329 PMC8066592 33915901

[312] Rebucci‐Peixoto M , Vienot A , Adotevi O , et al. A phase II study evaluating the interest to combine UCPVax, a telomerase CD4 T(H)1‐inducer cancer vaccine, and atezolizumab for the treatment of HPV positive cancers: volATIL study. Front Oncol. 2022;12:957580. 35928870 10.3389/fonc.2022.957580 PMC9343837

[313] Wang H , Che Y , Yang Y , Suo J , Wang X . Inhibition of orthotopic genital cancer induced by subcutaneous administration of human papillomavirus peptide vaccine with CpG oligodeoxynucleotides as an adjuvant in mice. Cancer Manag Res. 2021;13:5559‐5572. 34285577 10.2147/CMAR.S309226 PMC8285235

[314] Maynard SK , Marshall JD , MacGill RS , et al. Vaccination with synthetic long peptide formulated with CpG in an oil‐in‐water emulsion induces robust E7‐specific CD8 T cell responses and TC‐1 tumor eradication. BMC Cancer. 2019;19(1):540. 31170937 10.1186/s12885-019-5725-y PMC6555006

[315] He X , Zhou S , Quinn B , et al. HPV‐associated tumor eradication by vaccination with synthetic short peptides and particle‐forming liposomes. Small. 2021;17(11):e2007165. 33605054 10.1002/smll.202007165 PMC8011812

[316] Chandra J , Teoh SM , Kuo P , et al. Manganese‐doped silica‐based nanoparticles promote the efficacy of antigen‐specific immunotherapy. J Immunol. 2021;206(5):987‐998. 33504616 10.4049/jimmunol.2000355

[317] Da Silva DM , Skeate JG , Chavez‐Juan E , et al. Therapeutic efficacy of a human papillomavirus type 16 E7 bacterial exotoxin fusion protein adjuvanted with CpG or GPI‐0100 in a preclinical mouse model for HPV‐associated disease. Vaccine. 2019;37(22):2915‐2924. 31010714 10.1016/j.vaccine.2019.04.043 PMC6586561

[318] Morris VK , Jazaeri A , Westin SN , et al. Phase II trial of MEDI0457 and durvalumab for patients with recurrent/metastatic human papillomavirus‐associated cancers. Oncologist. 2023;28(7):618‐623. 10.1093/oncolo/oyad085 PMC10322132 37104874

[319] Miri SM , Pourhossein B , Hosseini SY , et al. Enhanced synergistic antitumor effect of a DNA vaccine with anticancer cytokine, MDA‐7/IL‐24, and immune checkpoint blockade. Virol J. 2022;19(1):106. 35752792 10.1186/s12985-022-01842-x PMC9233788

[320] Han KH , Jang MS , Han HY , et al. Preclinical safety assessment of a therapeutic human papillomavirus DNA vaccine combined with intravaginal interleukin‐7 fused with hybrid Fc in female rats. Toxicol Appl Pharmacol. 2021;413:115406. 33434572 10.1016/j.taap.2021.115406

[321] Chen YP , Lin CC , Xie YX , Chen CY , Qiu JT . Enhancing immunogenicity of HPV16 E(7) DNA vaccine by conjugating codon‐optimized GM‐CSF to HPV16 E(7) DNA. Taiwan J Obstet Gynecol. 2021;60(4):700‐705. 34247810 10.1016/j.tjog.2021.05.020

[322] Tahamtan A , Barati M , Tabarraei A , et al. Antitumor immunity induced by genetic immunization with chitosan nanoparticle formulated adjuvanted for HPV‐16 E7 DNA vaccine. Iran J Immunol. 2018;15(4):269‐280. 30593741 10.22034/IJI.2018.39396

[323] Hillemanns P , Denecke A , Woelber L , et al. A therapeutic antigen‐presenting cell‐targeting DNA vaccine VB10.16 in HPV16‐positive high‐grade cervical intraepithelial neoplasia: results from a phase I/IIa Trial. Clin Cancer Res. 2022;28(22):4885‐4892. 36129459 10.1158/1078-0432.CCR-22-1927

[324] Choi CH , Choi HJ , Lee JW , et al. Phase I study of a B cell‐based and monocyte‐based immunotherapeutic vaccine, BVAC‐C in human papillomavirus type 16‐ or 18‐Positive recurrent cervical cancer. J Clin Med. 2020;9(1). 10.3390/jcm9010147 PMC7019768 31948126

[325] Zhang L , Zhao Y , Tu Q , Xue X , Zhu X , Zhao KN . The roles of programmed cell death ligand‐1/programmed cell death‐1 (PD‐L1/PD‐1) in HPV‐induced cervical cancer and potential for their use in blockade therapy. Curr Med Chem. 2021;28(5):893‐909. 32003657 10.2174/0929867327666200128105459

[326] Ma Z , Zou X , Yan Z , Chen C , Chen Y , Fu A . Preliminary analysis of cervical cancer immunotherapy. Am J Clin Oncol. 2022;45(11):486‐490. 36301242 10.1097/COC.0000000000000950 PMC9624377

[327] Ferrall L , Lin KY , Roden RBS , Hung CF , Wu TC . Cervical cancer immunotherapy: facts and hopes. Clin Cancer Res. 2021;27(18):4953‐4973. 33888488 10.1158/1078-0432.CCR-20-2833 PMC8448896

[328] Wang Y , Wang C , Qiu J , et al. Targeting CD96 overcomes PD‐1 blockade resistance by enhancing CD8+ TIL function in cervical cancer. J Immunother Cancer. 2022;10(3):e003667. 35288463 10.1136/jitc-2021-003667 PMC8921917

[329] Franks SE , Fabian KP , Santiago‐Sánchez G , Wolfson B , Hodge JW . Immune targeting of three independent suppressive pathways (TIGIT, PD‐L1, TGFβ) provides significant antitumor efficacy in immune checkpoint resistant models. Oncoimmunology. 2022;11(1):2124666. 36211806 10.1080/2162402X.2022.2124666 PMC9542338

[330] Zhao J , Feng H , Wang T , Pang X , Zhou Y , Cui Y . The safety and efficacy of a novel method for treatment of HSIL. Arch Gynecol Obstet. 2021;304(5):1291‐1298. 33813597 10.1007/s00404-021-06047-1

[331] Guo Q , Chen W , Sun J , et al. Nocardia rubra cell‐wall skeleton activates an immune response in cervical tissue via stimulating FPR3 to enhance dendritic cell‐mediated Th1 differentiation. Front Immunol. 2023;14:1117545. 36936958 10.3389/fimmu.2023.1117545 PMC10018199

[332] Chen W , Zhang Y , Zhao C , et al. Nocardia rubra cell wall skeleton up‐regulates T cell subsets and inhibits PD‐1/PD‐L1 pathway to promote local immune status of patients with high‐risk human papillomavirus infection and cervical intraepithelial neoplasia. Front Immunol. 2020;11:612547. 33552075 10.3389/fimmu.2020.612547 PMC7856144

[333] Fonseca BO , Possati‐Resende JC , Salcedo MP , et al. Topical imiquimod for the treatment of high‐grade squamous intraepithelial lesions of the cervix: a randomized controlled trial. Obstet Gynecol. 2021;137(6):1043‐1053. 33957649 10.1097/AOG.0000000000004384 PMC8132915

[334] Rohaan MW , Wilgenhof S , Haanen JBAG . Adoptive cellular therapies: the current landscape. Virchows Arch Int J Pathol. 2019;474(4):449‐461. 10.1007/s00428-018-2484-0 PMC6447513 30470934

[335] Zhao Y , Deng J , Rao S , et al. Tumor infiltrating lymphocyte (TIL) therapy for solid tumor treatment: progressions and challenges. Cancers. 2022;14(17):4160. 36077696 10.3390/cancers14174160 PMC9455018

[336] Jiang J , Xia M , Zhang L , et al. Rapid generation of genetically engineered T cells for the treatment of virus‐related cancers. Cancer Sci. 2022;113(11):3686‐3697. 35950597 10.1111/cas.15528 PMC9633297

[337] Wang X , Sandberg ML , Martin AD , et al. Potent, selective CARs as potential T‐cell therapeutics for HPV‐positive cancers. J Immunother. 2021;44(8):292‐306. 34432728 10.1097/CJI.0000000000000386 PMC8415731

[338] Doran SL , Stevanović S , Adhikary S , et al. T‐cell receptor gene therapy for human papillomavirus‐associated epithelial cancers: a first‐in‐human, phase I/II study. J Clin Oncol. 2019;37(30):2759‐2768. 31408414 10.1200/JCO.18.02424 PMC6800280

[339] Honda T , Ando M , Ando J , et al. Sustainable tumor‐suppressive effect of iPSC‐derived rejuvenated T cells targeting cervical cancers. Mol Ther. 2020;28(11):2394‐2405. 32710827 10.1016/j.ymthe.2020.07.004 PMC7646217

[340] Huh WK , Brady WE , Fracasso PM , et al. Phase II study of axalimogene filolisbac (ADXS‐HPV) for platinum‐refractory cervical carcinoma: an NRG oncology/gynecologic oncology group study. Gynecol Oncol. 2020;158(3):562‐569. 32641240 10.1016/j.ygyno.2020.06.493 PMC7487015

[341] Park YC , Ouh YT , Sung MH , et al. A phase 1/2a, dose‐escalation, safety and preliminary efficacy study of oral therapeutic vaccine in subjects with cervical intraepithelial neoplasia 3. J Gynecol Oncol. 2019;30(6):e88. 31576684 10.3802/jgo.2019.30.e88 PMC6779607

[342] Mohseni AH , Taghinezhad SS , Keyvani H . The first clinical use of a recombinant lactococcus lactis expressing human papillomavirus type 16 E7 oncogene oral vaccine: a phase I safety and immunogenicity trial in healthy women volunteers. Mol Cancer Ther. 2020;19(2):717‐727. 31645442 10.1158/1535-7163.MCT-19-0375

[343] Harper DM , Nieminen P , Donders G , et al. The efficacy and safety of Tipapkinogen Sovacivec therapeutic HPV vaccine in cervical intraepithelial neoplasia grades 2 and 3: randomized controlled phase II trial with 2.5 years of follow‐up. Gynecol Oncol. 2019;153(3):521‐529. 30955915 10.1016/j.ygyno.2019.03.250

[344] Reuschenbach M , Pauligk C , Karbach J , et al. A phase 1/2a study to test the safety and immunogenicity of a p16INK4a peptide vaccine in patients with advanced human papillomavirus‐associated cancers. Cancer. 2016;122(9):1425–1433. 26949913 10.1002/cncr.29925

[345] Sousa LG , Rajapakshe K , Rodriguez Canales J , et al. ISA101 and nivolumab for HPV‐16(+) cancer: updated clinical efficacy and immune correlates of response. J Immunother Cancer. 2022;10(2). 10.1136/jitc-2021-004232 PMC9066369 35193933

[346] Speetjens FM , Welters MJP , Slingerland M , et al. Intradermal vaccination of HPV‐16 E6 synthetic peptides conjugated to an optimized Toll‐like receptor 2 ligand shows safety and potent T cell immunogenicity in patients with HPV‐16 positive (pre‐)malignant lesions. J Immunother Cancer. 2022;10(10). 10.1136/jitc-2022-005016 PMC9582304 36261215

[347] Einstein MH , Roden RBS , Ferrall L , et al. Safety run‐in of intramuscular pNGVL4a‐Sig/E7(detox)/HSP70 DNA and TA‐CIN protein vaccination as treatment for HPV16+ ASC‐US, ASC‐H, or LSIL/CIN1. Cancer Prev Res Phila. 2023;16(4):219‐227. 36607735 10.1158/1940-6207.CAPR-22-0413 PMC10068439

[348] Choi YJ , Hur SY , Kim TJ , et al. A phase II, prospective, randomized, multicenter, open‐label study of GX‐188E, an HPV DNA vaccine, in patients with cervical intraepithelial neoplasia 3. Clin Cancer Res. 2020;26(7):1616‐1623. 31727676 10.1158/1078-0432.CCR-19-1513

[349] Youn JW , Hur SY , Woo JW , et al. Pembrolizumab plus GX‐188E therapeutic DNA vaccine in patients with HPV‐16‐positive or HPV‐18‐positive advanced cervical cancer: interim results of a single‐arm, phase 2 trial. Lancet Oncol. 2020;21(12):1653‐1660. 33271094 10.1016/S1470-2045(20)30486-1

[350] Frenel JS , Le Tourneau C , O'Neil B , et al. Safety and efficacy of pembrolizumab in advanced, programmed death ligand 1‐positive cervical cancer: results from the phase Ib KEYNOTE‐028 Trial. J Clin Oncol. 2017;35(36):4035‐4041. 29095678 10.1200/JCO.2017.74.5471

[351] Santin AD , Deng W , Frumovitz M , et al. Phase II evaluation of nivolumab in the treatment of persistent or recurrent cervical cancer (NCT02257528/NRG‐GY002). Gynecol Oncol. 2020;157(1):161‐166. 31924334 10.1016/j.ygyno.2019.12.034 PMC7127981

[352] Naumann RW , Hollebecque A , Meyer T , et al. Safety and efficacy of nivolumab monotherapy in recurrent or metastatic cervical, vaginal, or vulvar carcinoma: results from the phase I/II CheckMate 358 trial. J Clin Oncol. 2019;37(31):2825‐2834. 31487218 10.1200/JCO.19.00739 PMC6823884

[353] Friedman CF , Snyder Charen A , Zhou Q , et al. Phase II study of atezolizumab in combination with bevacizumab in patients with advanced cervical cancer. J Immunother Cancer. 2020;8(2):e001126. 33004542 10.1136/jitc-2020-001126 PMC7534695

[354] Strauss J , Gatti‐Mays ME , Cho BC , et al. Bintrafusp alfa, a bifunctional fusion protein targeting TGF‐β and PD‐L1, in patients with human papillomavirus‐associated malignancies. J Immunother Cancer. 2020;8(2). 10.1136/jitc-2020-001395 PMC7745517 33323462

[355] Stevanović S , Draper LM , Langhan MM , et al. Complete regression of metastatic cervical cancer after treatment with human papillomavirus‐targeted tumor‐infiltrating T cells. J Clin Oncol. 2015;33(14):1543‐1550. 25823737 10.1200/JCO.2014.58.9093 PMC4417725

[356] Cohen AC , Roane BM , Leath CA . Novel therapeutics for recurrent cervical cancer: moving towards personalized therapy. Drugs. 2020;80(3):217‐227. 31939072 10.1007/s40265-019-01249-z PMC7033025

[357] Minion LE , Tewari KS . The safety and efficacy of bevacizumab in the treatment of patients with recurrent or metastatic cervical cancer. Expert Rev Anticancer Ther. 2017;17(3):191‐198. 27748633 10.1080/14737140.2016.1246187

[358] Shi HJ , Song H , Zhao QY , Tao CX , Liu M , Zhu QQ . Efficacy and safety of combined high‐dose interferon and red light therapy for the treatment of human papillomavirus and associated vaginitis and cervicitis: a prospective and randomized clinical study. Medicine (Baltimore). 2018;97(37):e12398. 30213012 10.1097/MD.0000000000012398 PMC6156011

[359] Xiong Y , Cui L , Bian C , Zhao X , Wang X . Clearance of human papillomavirus infection in patients with cervical intraepithelial neoplasia: a systemic review and meta‐analysis. Medicine (Baltimore). 2020;99(46):e23155. 33181688 10.1097/MD.0000000000023155 PMC7668491

[360] Stellato G . Intralesional recombinant alpha 2B interferon in the treatment of human papillomavirus‐associated cervical intraepithelial neoplasia. Sex Transm Dis. 1992;19(3):124‐126. 1326126

[361] Yang Y , Meng YL , Duan SM , et al. REBACIN® as a noninvasive clinical intervention for high‐risk human papillomavirus persistent infection. Int J Cancer. 2019;145(10):2712‐2719. 30989655 10.1002/ijc.32344

[362] Yang Y , Hu T , Ming X , Yang E , Min W , Li Z . REBACIN® is an optional intervention for persistent high‐risk human papillomavirus infection: a retrospective analysis of 364 patients. Int J Gynaecol Obstet. 2021;152(1):82‐87. 32966600 10.1002/ijgo.13385

[363] Zhou SG , Wu DF , Yao H , et al. REBACIN(®) inhibits E6/E7 oncogenes in clearance of human papillomavirus infection. Front Oncol. 2022;12:1047222. 36561517 10.3389/fonc.2022.1047222 PMC9763439

[364] Mei Z , Li D . The role of probiotics in vaginal health. Front Cell Infect Microbiol. 2022;12:963868. 35967876 10.3389/fcimb.2022.963868 PMC9366906

[365] Palma E , Recine N , Domenici L , Giorgini M , Pierangeli A , Panici PB . Long‐term Lactobacillus rhamnosus BMX 54 application to restore a balanced vaginal ecosystem: a promising solution against HPV‐infection. BMC Infect Dis. 2018;18(1):13. 29304768 10.1186/s12879-017-2938-z PMC5756375

[366] Ou YC , Fu HC , Tseng CW , Wu CH , Tsai CC , Lin H . The influence of probiotics on genital high‐risk human papilloma virus clearance and quality of cervical smear: a randomized placebo‐controlled trial. BMC Womens Health. 2019;19(1):103. 31340789 10.1186/s12905-019-0798-y PMC6657108

[367] Hu S , Hao Y , Zhang X , et al. Lacticaseibacillus casei LH23 suppressed HPV gene expression and inhibited cervical cancer cells. Probiotics Antimicrob Proteins. 2023;15(3):443‐450. 34599740 10.1007/s12602-021-09848-7

[368] Hu Y , Lu Y , Qi X , et al. Clinical efficacy of paiteling in the treatment of condyloma acuminatum infected with different subtypes of HPV. Dermatol Ther. 2019;32(5):e13065. 31414707 10.1111/dth.13065

[369] Nikakhtar Z , Hasanzadeh M , Hamedi SS , et al. The efficacy of vaginal suppository based on myrtle in patients with cervicovaginal human papillomavirus infection: a randomized, double‐blind, placebo trial. Phytother Res. 2018;32(10):2002‐2008. 29943384 10.1002/ptr.6131

[370] Basu P , Dutta S , Begum R , et al. Clearance of cervical human papillomavirus infection by topical application of curcumin and curcumin containing polyherbal cream: a phase II randomized controlled study. Asian Pac J Cancer Prev. 2013;14(10):5753‐5759. 24289574 10.7314/apjcp.2013.14.10.5753

[371] Ding W , Ji T , Xiong W , Li T , Pu D , Liu R . Realgar, a traditional Chinese medicine, induces apoptosis of HPV16‐positive cervical cells through a HPV16 E7‐related pathway. Drug Devel Ther. 2018;12:3459‐3469. 10.2147/DDDT.S172525 PMC6197830 30410307

[372] Obasi TC , Braicu C , Iacob BC , et al. Securidaca‐saponins are natural inhibitors of AKT, MCL‐1, and BCL2L1 in cervical cancer cells. Cancer Manag Res. 2018;10:5709‐5724. 30532593 10.2147/CMAR.S163328 PMC6245348

[373] Ghanbari A , Le Gresley A , Naughton D , Kuhnert N , Sirbu D , Ashrafi GH . Biological activities of Ficus carica latex for potential therapeutics in human papillomavirus (HPV) related cervical cancers. Sci Rep. 2019;9(1):1013. 30705373 10.1038/s41598-018-37665-6 PMC6355798

[374] Zhao X , Song X , Zhao J , et al. Juglone inhibits proliferation of HPV‐positive cervical cancer cells specifically. Biol Pharm Bull. 2019;42(3):475‐480. 30606896 10.1248/bpb.b18-00845

[375] Kori M , Arga KY , Mardinoglu A , Turanli B . Repositioning of anti‐inflammatory drugs for the treatment of cervical cancer sub‐types. Front Pharmacol. 2022;13:884548. 35770086 10.3389/fphar.2022.884548 PMC9234276

[376] Hoppe‐Seyler K , Herrmann AL , Däschle A , et al. Effects of metformin on the virus/host cell crosstalk in human papillomavirus‐positive cancer cells. Int J Cancer. 2021;149(5):1137‐1149. 33844847 10.1002/ijc.33594

[377] Trimble CL , Levinson K , Maldonado L , et al. A first‐in‐human proof‐of‐concept trial of intravaginal artesunate to treat cervical intraepithelial neoplasia 2/3 (CIN2/3). Gynecol Oncol. 2020;157(1):188‐194. 32005582 10.1016/j.ygyno.2019.12.035

[378] Braun JA , Herrmann AL , Blase JI , et al. Effects of the antifungal agent ciclopirox in HPV‐positive cancer cells: repression of viral E6/E7 oncogene expression and induction of senescence and apoptosis. Int J Cancer. 2020;146(2):461‐474. 31603527 10.1002/ijc.32709

[379] Cui Z , Liu H , Zhang H , et al. The comparison of ZFNs, TALENs, and SpCas9 by GUIDE‐seq in HPV‐targeted gene therapy. Mol Ther Nucleic Acids. 2021;26:1466‐1478. 34938601 10.1016/j.omtn.2021.08.008 PMC8655392

[380] Noroozi Z , Shamsara M , Valipour E , et al. Antiproliferative effects of AAV‐delivered CRISPR/Cas9‐based degradation of the HPV18‐E6 gene in HeLa cells. Sci Rep. 2022;12(1):2224. 35140292 10.1038/s41598-022-06025-w PMC8828776

[381] Xiong J , Tan S , Yu L , et al. E7‐targeted nanotherapeutics for key HPV afflicted cervical lesions by employing CRISPR/Cas9 and Poly (Beta‐Amino Ester). Int J Nanomed. 2021;16:7609‐7622. 10.2147/IJN.S335277 PMC8606985 34819726

[382] Idres YM , Lai AJ , McMillan NAJ , Idris A . Hyperactivation of p53 using CRISPRa kills human papillomavirus‐driven cervical cancer cells. Virus Genes. 2023;59(2):312‐316. 36474086 10.1007/s11262-022-01960-2

[383] Gao C , Wu P , Yu L , et al. The application of CRISPR/Cas9 system in cervical carcinogenesis. Cancer Gene Ther. 2022;29(5):466‐474. 34349239 10.1038/s41417-021-00366-w PMC9113934

[384] Jubair L , Lam AK , Fallaha S , McMillan NAJ . CRISPR/Cas9‐loaded stealth liposomes effectively cleared established HPV16‐driven tumours in syngeneic mice. PLoS One. 2021;16(1):e0223288. 33411765 10.1371/journal.pone.0223288 PMC7790238

[385] Chen Y , Jiang H , Wang T , et al. In vitro and in vivo growth inhibition of human cervical cancer cells via human papillomavirus E6/E7 mRNAs’ cleavage by CRISPR/Cas13a system. Antivir Res. 2020;178:104794. 32298665 10.1016/j.antiviral.2020.104794

[386] Perkins RB , Guido RS , Castle PE , et al. 2019 ASCCP risk‐based management consensus guidelines for abnormal cervical cancer screening tests and cancer precursors. J Low Genit Tract Dis. 2020;24(2):102‐131. 32243307 10.1097/LGT.0000000000000525 PMC7147428

[387] Santesso N , Mustafa RA , Wiercioch W , et al. Systematic reviews and meta‐analyses of benefits and harms of cryotherapy, LEEP, and cold knife conization to treat cervical intraepithelial neoplasia. Int J Gynaecol Obstet. 2016;132(3):266‐271. 26643302 10.1016/j.ijgo.2015.07.026

[388] Kwiatkowski S , Knap B , Przystupski D , et al. Photodynamic therapy—mechanisms, photosensitizers and combinations. Biomed Pharmacother Biomed Pharmacother. 2018;106:1098‐1107. 30119176 10.1016/j.biopha.2018.07.049

[389] Unanyan A , Pivazyan L , Davydova J , et al. Efficacy of photodynamic therapy in women with HSIL, LSIL and early stage squamous cervical cancer: a systematic review and meta‐analysis. Photodiagnosis Photodyn Ther. 2021;36:102530. 34534688 10.1016/j.pdpdt.2021.102530

[390] Wang L , Liu X , Zhang J , et al. Evaluation of the efficacy and safety of 5‐aminolevulinic acid‐mediated photodynamic therapy in women with high‐risk HPV persistent infection after cervical conization. Photodiagnosis Photodyn Ther. 2022;40:103144. 36210038 10.1016/j.pdpdt.2022.103144

[391] Zhao XL , Liu ZH , Zhao S , et al. Efficacy of point‐of‐care thermal ablation among high‐risk human papillomavirus positive women in China. Int J Cancer. 2021;148(6):1419‐1427. 32895912 10.1002/ijc.33290

[392] Duan L , Du H , Belinson JL , et al. Thermocoagulation versus cryotherapy for the treatment of cervical precancers. J Obstet Gynaecol Res. 2021;47(1):279‐286. 33089619 10.1111/jog.14520 PMC7820992

[393] Wang W , Liu Y , Yang Y , Ren J , Zhou H . Changes in vaginal microbiome after focused ultrasound treatment of high‐risk human papillomavirus infection‐related low‐grade cervical lesions. BMC Infect Dis. 2023;23(1):3. 36604622 10.1186/s12879-022-07937-8 PMC9814320

[394] Wang W , Liu Y , Pu Y , Li C , Zhou H , Wang Z . Effectiveness of focused ultrasound for high risk human papillomavirus infection‐related cervical lesions. Int J Hyperth. 2021;38(2):96‐102. 10.1080/02656736.2021.1910736 34420437

